# Constraints on Biological Mechanism from Disease Comorbidity Using Electronic Medical Records and Database of Genetic Variants

**DOI:** 10.1371/journal.pcbi.1004885

**Published:** 2016-04-26

**Authors:** Steven C. Bagley, Marina Sirota, Richard Chen, Atul J. Butte, Russ B. Altman

**Affiliations:** 1 Department of Genetics, Stanford University, Stanford, California, United States of America; 2 UCSF Institute for Computational Health Sciences, San Francisco, California, United States of America; 3 Personalis, Inc., Menlo Park, California, United States of America; 4 Department of Bioengineering, Stanford University, Stanford, California, United States of America; University of Maryland Baltimore County, UNITED STATES

## Abstract

Patterns of disease co-occurrence that deviate from statistical independence may represent important constraints on biological mechanism, which sometimes can be explained by shared genetics. In this work we study the relationship between disease co-occurrence and commonly shared genetic architecture of disease. Records of pairs of diseases were combined from two different electronic medical systems (Columbia, Stanford), and compared to a large database of published disease-associated genetic variants (VARIMED); data on 35 disorders were available across all three sources, which include medical records for over 1.2 million patients and variants from over 17,000 publications. Based on the sources in which they appeared, disease pairs were categorized as having predominant clinical, genetic, or both kinds of manifestations. Confounding effects of age on disease incidence were controlled for by only comparing diseases when they fall in the same cluster of similarly shaped incidence patterns. We find that disease pairs that are overrepresented in both electronic medical record systems and in VARIMED come from two main disease classes, autoimmune and neuropsychiatric. We furthermore identify specific genes that are shared within these disease groups.

## Author Summary

Diseases do not always occur together at random, and patterns of disease association may reflect important biological constraints on disease mechanism. When a disease pair occurs more (or less) often than would be expected by chance given the frequencies of each disease separately, that may signal the presence of shared causal factors. These shared factors may be genetic, environmental, or interactions of the two. To characterize the kinds of possible disease causes, we compared data from electronic medical records, which record disease manifestations, with information about genetic variants of disease. In particular, we find pairs of diseases that occur more or less often than expected by chance for patients in two electronic medical record systems, and compare them to disease pairs sharing a statistically significant number of genes in a database of disease-associated genetic variants. Overrepresented pairs appearing in both source types come from two main disease classes, autoimmune and neuropsychiatric.

## Introduction

When two diseases occur together in the same individuals more or less often than would be expected by chance, this may signal the operation of important biological processes. Pairs of diseases occurring more than expected are called synergistic; such interactions are familiar from clinical practice when the occurrence of a disease may raise the risk of a second disease. Pairs occurring less than expected are called protective; these interactions, sometimes called “inverse comorbidities,” are less common, but intriguing. Disease pairs which consistently diverge from independence in either direction may provide clues towards identifying core genetic, pathway, physiological, or environmental constraints that alter disease risk and represent an important starting point for elaborating a mechanistic understanding of disease and for locating possible drug targets. Because discovery of disease patterns has been haphazard, it is attractive to systematically search for these patterns across a wide range of diseases, without adhering to prior conceptions of disease class, associated features, or expected comorbidities. In this work, we integrate clinical and genomic data across diseases to systematically assess their co-occurrence.

Consistent co-occurrence and conditional dependence in disease phenotypes arises from multiple, non-exclusive, factors: (1) shared genetics, including causal effects of single genes and effects of neighboring genes in linkage disequilibrium, (2) shared environmental exposures, (3) complex interactions in which a phenotype enhances or moderates the risk of another, (4) ascertainment, selection, or referral bias, (5) artifacts of the diagnostic system, where two putatively separate diseases are linked via large overlap of shared features, and (6) random variation. Untangling these factors requires use and integration of both phenotypic and genetic data.

Historically, non-independent phenotype associations are noticed in an opportunistic way when the effect size is large, and otherwise they are detected more accurately through observational studies and meta-analyses [1], or via comprehensive epidemiologic surveys. However, such studies and surveys are expensive to conduct and therefore often do not methodically examine disease combinations. In contrast, electronic medical records (EMR) represent a source of coded medical data that is typically large and, because these records are routinely collected to support clinical and administrative operations, the marginal cost to researchers is small; EMR data may therefore facilitate systematic comparison of disease co-occurrence [2].

Complementary information about disease relationships can be drawn from genomic studies. In particular, VARiants Informing MEDicine, or VARIMED [3], is a hand-curated database of published disease-associated (primarily common) genetic variants. Although it is limited to known genetic variants, it is large and provides an opportunity for detecting the overlapping and shared genetic bases of diseases.

We combine EMR data with genetic data to compare and contrast disease co-occurrence patterns, systematically comparing statistically significant disease comorbidity patterns in EMR data with disease pairs having statistically significant genetic overlap in VARIMED, and characterize the pairs by the predominant influence as (1) clinical and genetic if they both co-occur in the clinical data and share a significant genetic component, (2) clinical without genetic if they co-occur only in the clinical records, or (3) genetic without clinically observable effect if we find only a significant genetic overlap without a corresponding EMR result.

There are several important assumptions to consider here including the penetrance and causality of the genetic relationships that we examine as well as interactions between the genetics and the environment. Furthermore, EMR data are prone to selection and ascertainment bias, and errors from inaccuracies in chart coding. The lifetime of the EMR induces an observation window on the patients represented there, underrecording data from patients for disease pairs with widely separated ages of disease onset, and generating false inverse comorbidities. In order to avoid the confounding effect of age on the pair occurrence counts, we introduce a method for clustering diseases through similarity of their incidence pattern by age.

Other researchers have explored similar ideas. Patterns have been detected using linked administrative and clinical databases. Goldacre and colleagues [4] used data from the Oxford Record Linkage Study to find disease associations, such as an expected association between schizophrenia and lung cancer, and a protective association between schizophrenia and rheumatoid arthritis. A later study using similar data [5] found inverse associations between Parkinson’s disease and several kinds of cancer. Rzhetsky et al. [2] developed a mathematical model of ICD9-coded data from a single EMR to infer genetic overlap. Using genomics data from early GWAS studies, Sirota et al. [6] used summary data to define a signed genetic variation score and cluster autoimmune disorders. Jung et al. [7] applied a similar method to studying autoimmune disorders when paired with autism. Li et al. [8] used data from several EMRs and from VARIMED to identify the genetic architecture of novel risk factor-disease associations. Ibáñez et al. [9] compared gene expression profiles for previously identified inversely comorbid neuropsychiatric/cancer disease pairs, and found corresponding up- and down-regulation patterns. Melamed et al. [10] used data from a large database of insurance claims in combination with known genetic associations for Mendelian disorders to identify cancer driver genes. Glicksberg et al. [11] compared the overlap in disease pairs using EMR data and a database of genetic variants, retaining those pairs where both diseases appeared together in PubMed articles.

In this paper, we present a framework for integrating clinical and molecular data to study disease co-occurrence. Because disease risk varies with patient age, and because the co-occurrence of disease is therefore confounded by age, we introduce a method to define age-specific disease clusters and carry out pairwise comparisons of disease co-occurrence. We explicitly model disease pair under and overrepresentation. To reduce bias, we conduct the analysis in two independent clinical databases, and require statistically significant deviation from independence in both. We identify a highly significant group of autoimmune disorders, a set of diseases with known environmental triggers, and some results which question the clinical manifestation of previously described disease associations.

## Results

**Fig 1:**
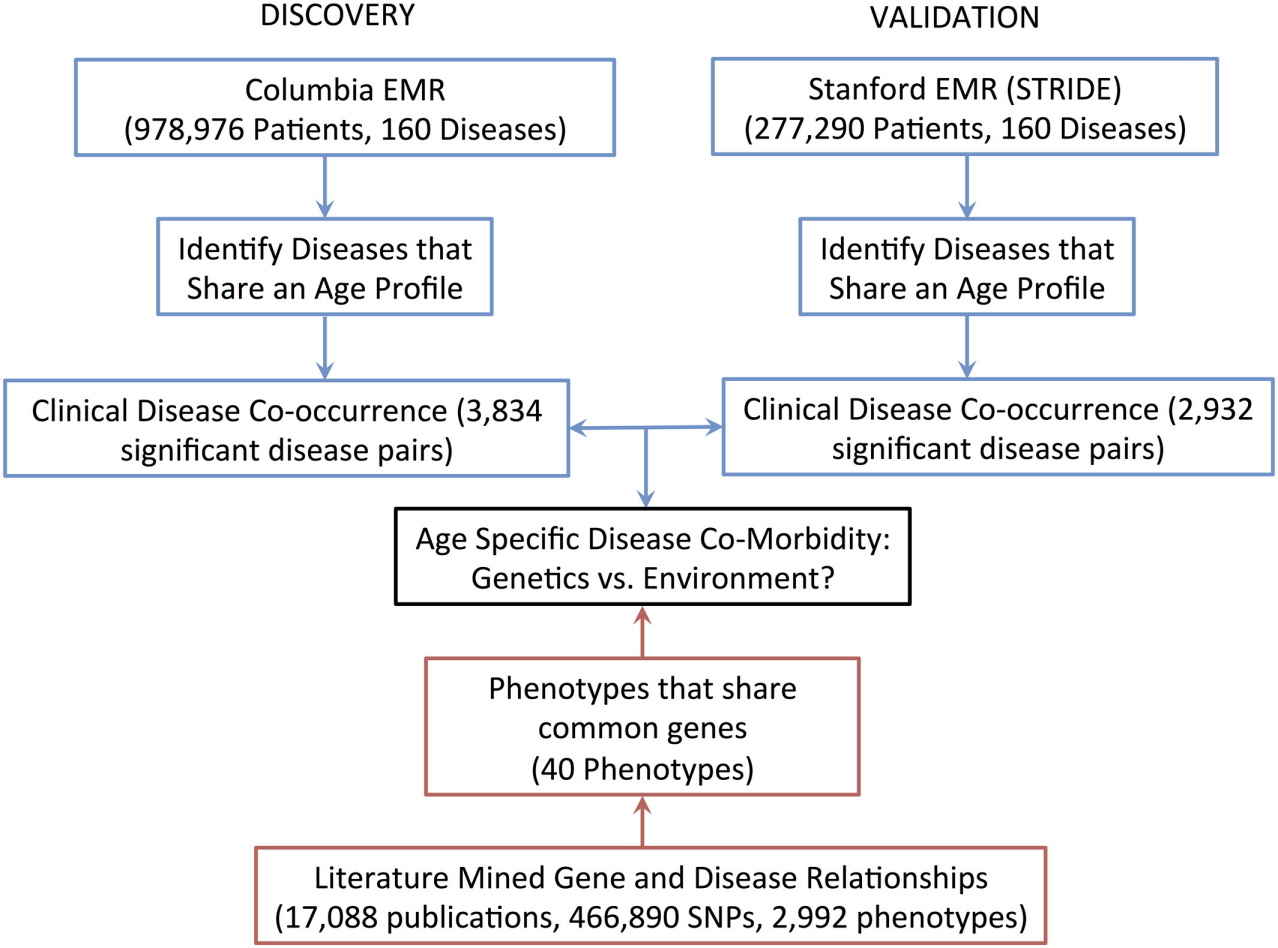
The overall information flow. Clinical data on disease co-occurrence from the Columbia and Stanford EMRs were compared to the literature-mined gene and disease relationships in the VARIMED database.

Data on disease pairs were drawn from Columbia and Stanford electronic medical record systems, and compared to data on disease pairs with genetic overlap from the VARIMED database. The overall information flow is shown in Fig 1.

Disease comorbidities were assessed for significance using a conventional 2 × 2 table recording the presence or absence of each disease; a patient contributes a count to one of the four cells (Table 1.) In aggregate, there are three possibilities: (1) independence, where the value of d follows proportionally from the marginal sums, (2) synergistic interactions, where d is larger than predicted from independence, and the pairs are overrepresented, or (3) protective interactions, where d is smaller than predicted, and the pairs are underrepresented.

**Table 1:** Conventional 2 × 2 table for counting the presence/absence of a disease pair.

	*not Disease* 2	*Disease 2*
*not Disease 1*	a	b
*Disease 1*	c	d

Disease pairs were collated by the sources in which they were found to be significant: (1) significant in both EMRs and significant in VARIMED, or “clinical and genetic,”, (2) in both EMRs but not in VARIMED, or “clinical without genetic,” and (3) in VARIMED but not in both EMRs, or “genetic without observed clinical effect.” (See Table 2.) Appearance in a clinical database represents the interaction between genetic predispositions, environmental exposures, and socioeconomic and phenotypic factors that lead to presentation for evaluation and treatment.

**Table 2:** Informal names for each combination of statistically significant results from EMRs and VARIMED.

*EMR*	*VARIMED*	*Interpretation*
+	+	“Clinical and genetic”
+	−	“Clinical without observed genetic effect”
−	+	“Genetic with no observed clinical effect”

+ = significant,

− = not significant.

We start with 161 disorders in the available EMR data, of which 35 disorders appear in both EMRs and also in VARIMED; these are listed in Table 3, along with disease counts and frequencies for each EMR, and the gene counts from VARIMED.

**Table 3:** Counts and frequencies (as percent) for diseases that occur in both EMR data sets and in VARIMED. Number of genes is from VARIMED. Cluster names were assigned by hand to facilitate comprehension, as described in the text.

		Columbia		Stanford			
	Disease name	Count	Percent	Count	Percent	Number of genes	Cluster name
1	Alcoholism	27638	2.82	11363	4.10	81	adulthood
2	Allergic rhinitis	19216	1.96	22523	8.12	5	other
3	Alopecia areata	821	0.08	632	0.23	75	other
4	Alzheimer’s	9073	0.93	2444	0.88	179	aged
5	Amyotrophic lateral sclerosis	2182	0.22	276	0.10	70	aged
6	Ankylosing spondylitis	510	0.05	532	0.19	38	adulthood
7	Aortic aneurysm	2990	0.31	5401	1.95	22	aged
8	Attention deficit	6964	0.71	5043	1.82	93	youth
9	Autism	481	0.05	2423	0.87	218	youth
10	Behcet’s s.	53	0.01	82	0.03	42	other
11	Bipolar disorder	12373	1.26	7179	2.59	185	adulthood
12	Cardiomyopathy	11457	1.17	8212	2.96	4	aged
13	Celiac sprue	1954	0.20	1267	0.46	45	other
14	Cholelithiasis	15353	1.57	8095	2.92	5	aged
15	Depression	27085	2.77	8283	2.99	155	adulthood
16	Diabetes type 1	19372	1.98	5116	1.84	323	other
17	Diabetes type 2	60815	6.21	40176	14.49	254	aged
18	Epilepsy	12099	1.24	12095	4.36	9	neonate
19	Goiter	10820	1.11	9201	3.32	5	adulthood
20	Gout	192	0.02	106	0.04	12	aged
21	HIV	6138	0.63	1073	0.39	92	adulthood
22	Hepatitis B	5757	0.59	3212	1.16	14	adulthood
23	Hepatitis C	18421	1.88	6583	2.37	40	aged
24	Hypertrophic cardiomyopathy	603	0.06	831	0.30	4	adulthood
25	Kawasaki’s d.	495	0.05	328	0.12	66	youth
26	Migraine	8049	0.82	12593	4.54	18	adulthood
27	Moyamoya	130	0.01	557	0.20	8	other
28	Multiple sclerosis	14979	1.53	1685	0.61	261	adulthood
29	Parkinson’s d.	6116	0.62	2839	1.02	151	aged
30	Psoriasis	4577	0.47	3249	1.17	104	adulthood
31	Rheumatoid arthritis	7333	0.75	4775	1.72	348	aged
32	Schizophrenia	11256	1.15	1935	0.70	208	adulthood
33	Sjogren’s s.	348	0.04	893	0.32	7	aged
34	Systemic lupus erythematosus	3194	0.33	2090	0.75	175	adulthood
35	Tuberculosis	66569	6.80	912	0.33	32	adulthood

**Fig 2:**
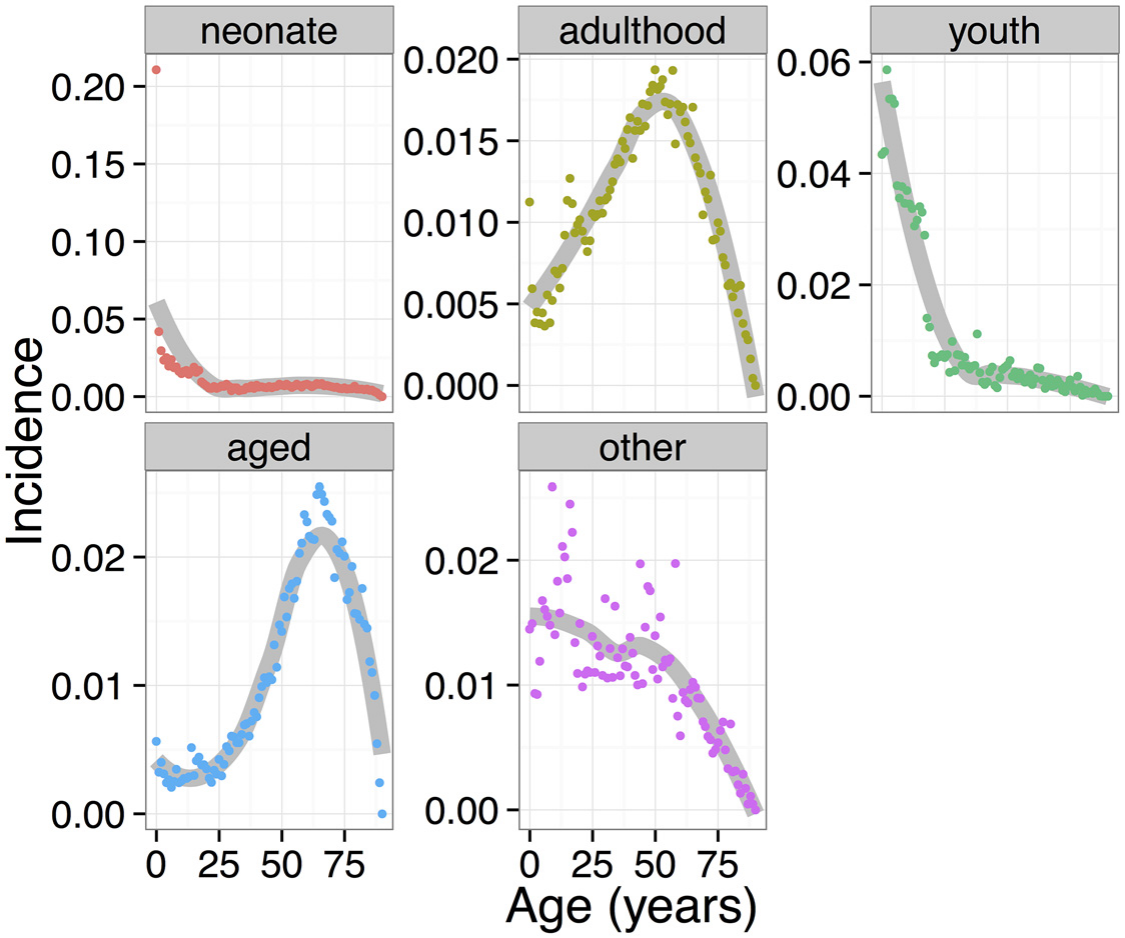
The incidence-by-age patterns of the five clusters identified. Using data from Stanford’s EMR, each graph shows the incidence at each age, averaged over all disorders in the cluster. The loess smoother marks the overall trend with a colored band. The same cluster colors are used throughout this paper. See the text for description of the cluster names.

To avoid the confounding effects of age on disease incidence, we form age-incidence clusters, where cluster members have similar age-incidence patterns, and only analyze disease pairs where both members of the pair fall in the same cluster. We use a data-driven method to compute cluster size, finding a locally optimum size of five. For visualization, each cluster is processed by forming the average of the incidence vectors in that cluster; these averages, along with a loess smoother, are shown in Fig 2. Plots of all the data points for all examined cluster sizes appear in the Appendix. Conveniently, four of the clusters correspond to different life-stages (neonate, youth, adulthood, and aged) and were assigned those names by hand for ease of reference and to aid interpretation; the names are also listed in the final column of Table 3. The fifth cluster contains data from predominantly younger patients, but is noisier and less consistent than the other clusters; it is labelled “other.” No significant results were found for diseases in the “neonate” cluster, so that cluster does not appear in the tables below. A complete list of all EMR disorders appearing in each cluster appears in the Supplement.

**Fig 3:**
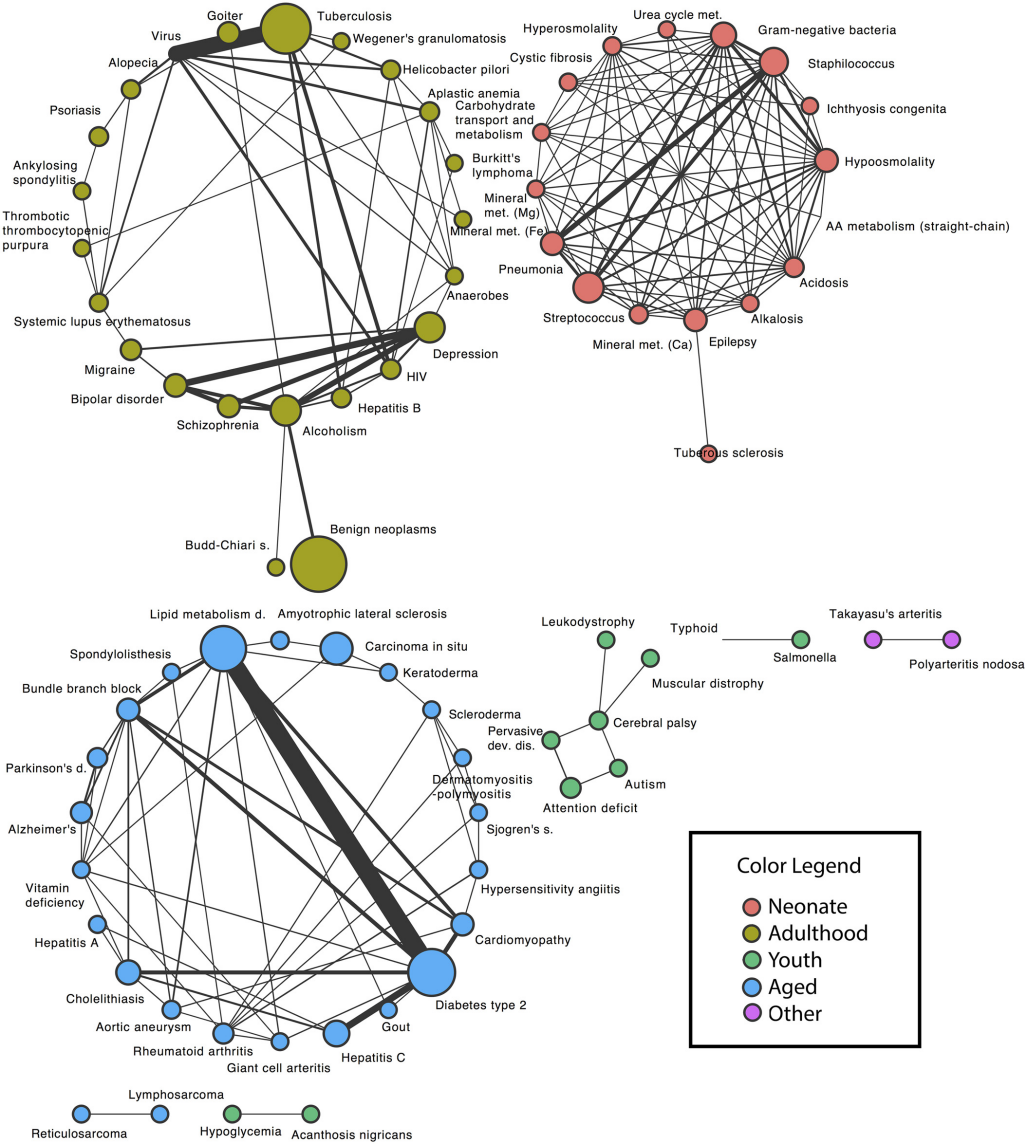
Network structure of the significant disease pairs that occur in both EMRs. Each node represents a disease, with the node size scaled to the disease frequency in the Columbia EMR. Each edge connects statistically significant pairs, with the edge width scaled to the effect size (observed number divided by expected number). Node color corresponds to the cluster colors in Fig 2.

Significant disease pairs are presented in overview here and described in detail in the following sections. For each of the two EMRs, a significant disease pair is either under or overrepresented. We consider only those results that show concordant results, either both underrepresented, or both overrepresented, in the two EMRs. The structure of the overrepresented disease pairs is seen in the network diagram in Fig 3; this figure uses data from the larger EMR (Columbia) to set the node size from disease frequency. There are three large components in the network, which have been coded by the color of the age-incidence cluster that forms each component; all have a compact, densely connected structure with only a few sparse ties, in spite of coming from an arbitrarily chosen list of common and rare diseases. The strongest effect is shown with thick link, marking the connection between lipid metabolism disorders and type 2 diabetes. The small light-green cluster (middle of lower row in figure) highlights the connections between autism, pervasive developmental disorder, attention deficit, and cerebral palsy.

**Fig 4:**
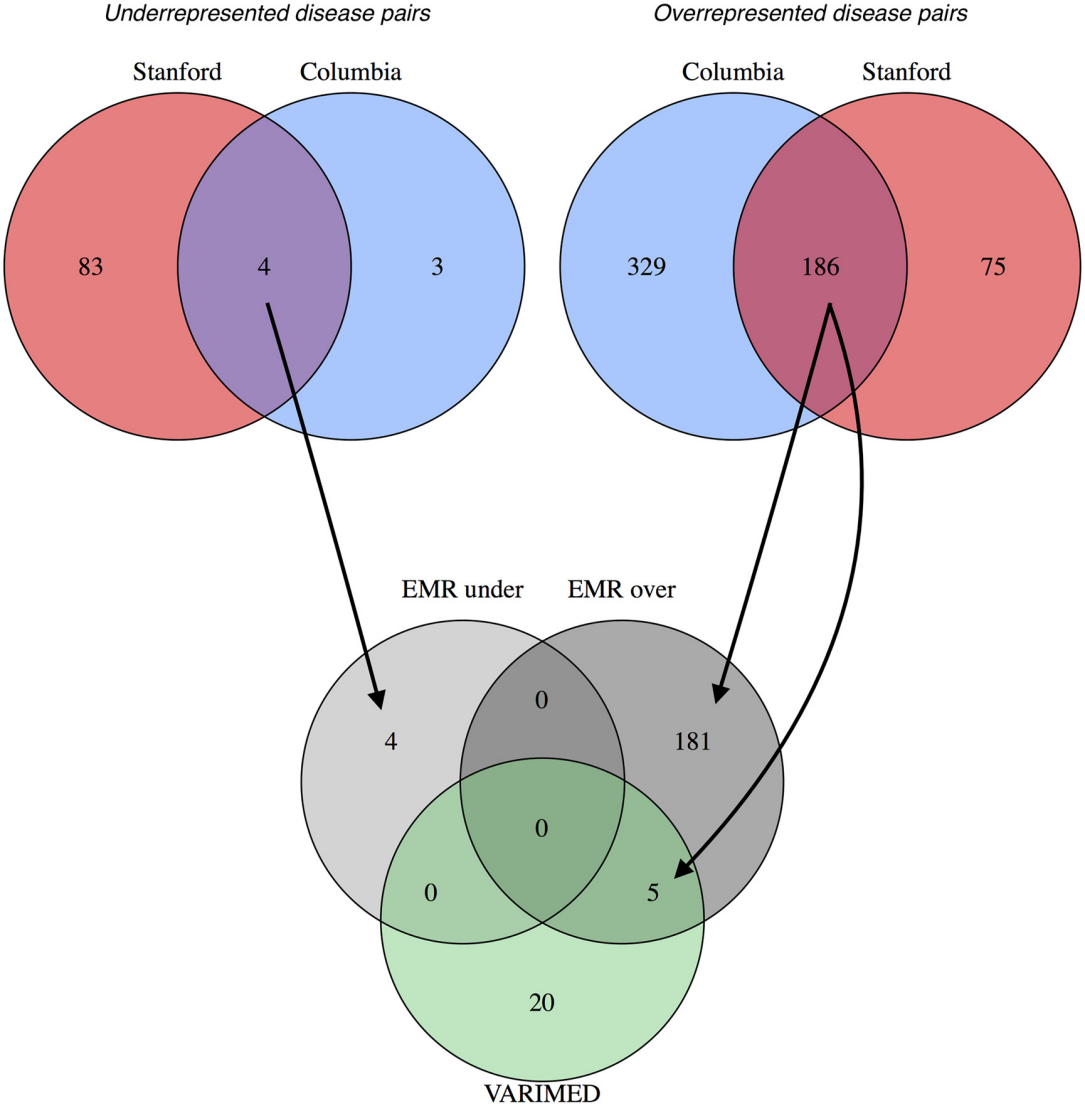
Venn diagrams showing the overlap of the disease pairs from the two electronic medical records and from VARIMED. At the top, the leftmost diagram shows the overlap of statistically significant disease pairs that are underrepresented in Columbia and in Stanford; the rightmost diagram is for overrepresented pairs. The bottom diagram shows the overlap between the conjunctions (overlapping regions) of the upper diagrams and the disease pairs in VARIMED. Arrows show how the results from the EMR sources were combined with the VARIMED results. The counts of disease pairs shown do not correspond exactly to those in Tables 6 and 7 because the VARIMED results here include discordant pairs, underrepresented in one EMR and overrepresented in the other.

The set relationship of disease-pairs from the EMR data compared to the genetic variants is shown in the Venn diagram (Fig 4). For the underrepresented disease pairs, four are shared between Columbia and Stanford, but none appear in the intersection with VARIMED. For the overrepresented disease pairs, 186 are shared between Columbia and Stanford, and five of those remain when intersected with VARIMED.

### “Clinical and genetic” results

In this section we report the results that are significant in both EMRs (Columbia and Stanford) and in VARIMED. We refer to these as “clinical and genetic.” For disease pairs significant in Columbia, Stanford, and in VARIMED, none were protective, and five were synergistic. The results, which fall into two classes, autoimmune and neuropsychiatric, are shown in Table 4. Information about the genes and gene overlap for the five overrepresented pairs appears in Table 5.

**Table 4:** Results for overrepresented (synergistic) disease pairs that are significant in Columbia and Stanford EMRs and in VARIMED. Results are sorted by cluster and by Obs/Exp within each cluster.

				Columbia		Stanford	
	Disease 1	Disease 2	Cluster name	Obs/Exp	P-value	Obs/Exp	P-value
1	Ankylosing spondylitis	Psoriasis	adulthood	7.13	6.22E-10	2.57	6.85E-04
2	Ankylosing spondylitis	Systemic lupus erythematosus	adulthood	46.88	3.13E-102	3.24	2.56E-04
3	Bipolar disorder	Depression	adulthood	16.27	0.00E+00	7.07	0.00E+00
4	Bipolar disorder	Schizophrenia	adulthood	22.34	0.00E+00	10.16	0.00E+00
5	Rheumatoid arthritis	Sjogren’s s.	aged	35.29	2.33E-111	10.92	1.51E-117

Obs/Exp = Observed/Expected.

**Table 5:** Results of overrepresented disease pairs that are significant in Columbia and Stanford EMRs and in VARIMED, showing the genetic information from VARIMED. disease1/2 genes = number of genes for each disease, gene overlap = number of shared genes, pvalue = pvalue from Fisher exact test, OR = odds ratio, gene names = the gene symbols for the shared genes. Colons connect groups of genes all mapped from the same variant.

	disease1	disease2	disease1 genes	disease2 genes	gene overlap	pvalue	OR	gene names
1	Ankylosing spondylitis	Psoriasis	38	104	9	0.00	55.60	CAST:ERAP1, ERAP1, HCP5, HLA-E, IL23R, MICA, MUC22, PSORS1C3, PTPN1
2	Ankylosing spondylitis	Systemic lupus erythematosus	38	175	9	0.00	32.96	ABCF1, BTNL2, GPSM3, HCG23, HCP5, IL23R, MSH5:MSH5-SAPCD1, MUC22, TRIM31
3	Bipolar disorder	Depression	185	155	40	0.00	33.11	ANK3, ANKS1B, BBS1, BCL11B, C11orf80, C15orf53, CACNA1C, CDH13, CNNM4, CNNM4:MIR3127, CNTNAP5, DDN, FER1L5, GLT8D1, GLT8D1:GNL3, GLT8D1:SPCS1, GNL3:PBRM1, GNL3:PBRM1:SNORD19, GNL3:SNORD69, ITIH1, ITIH3, ITIH4, KMT2D, LMAN2L, MACROD2, MAPK10, MUC22, NEK4, NFIX, PBRM1, PDE7B, PELI3, PRKAG1, REV1, SPCS1, SVEP1, SYNE1, TENM4, TMEM132D, ZNF804A
4	Bipolar disorder	Schizophrenia	185	208	10	0.00	5.10	ANK3, CACNA1C, CDH13, GPM6A, ITIH4, MAD1L1, MYO5B, PDE7B, PTPRG, ZNF804A
5	Rheumatoid arthritis	Sjogren’s s.	348	7	4	0.00	31.15	LOC100287329:LTA, LST1, LST1:NCR3, TNF

Prior work has found considerable genetic sharing between many autoimmune diseases [6], [12]; specific results include, ankylosing spondylitis and psoriasis [13]. The association between ankylosing spondylitis and lupus has been reported, but is extremely rare [14]. Rheumatoid arthritis and secondary Sjogren’s syndrome have a well-known association. The other two results are previously identified associations between neuropsychiatric disorders; bipolar disorder and schizophrenia [15], and bipolar disorder and depression, although there may also be diagnostic overlap, as depression and bipolar disorder can be confused clinically.

We furthermore identify specific genes which are common to these two groups (Table 5). In the autoimmune subgroup those include well known associations in the HLA region such as *HLA-DRA*, *HLA-E*[16], interleukin receptors (*IL13*, *IL23R* and *IL2RA*) [17, 18], [19], *BTNL2*[20] and *MICA*[21]. Interleukins are any of a class of glycoproteins produced by leukocytes for regulating immune responses. While these genes have been previously associated with autoimmune diseases, they provide an interesting opportunity to explore shared therapeutic targets and diagnostic markers across these phenotypes.

In the neuropsychiatric subgroup some genes that are of interest include *ANK3*, *CACNA1C*, *CDH13*, *ITIH4* and *PDE7B*. Ankyrins are a family of proteins that are believed to link the integral membrane proteins and play key roles in activities such as cell motility, activation, proliferation, contact, and the maintenance of specialized membrane domains. Ankyrin 3 is an immunologically distinct gene product from ankyrins 1 and 2, and was originally found at the axonal initial segment and nodes of Ranvier of neurons in the central and peripheral nervous systems. *CACNA1C* is a voltage-dependent calcium channel and has been previously linked to several neurodegenerative diseases [22], [23]. *CACNA1C* is also an associated gene of the one of the most highly significant SNPs for both bipolar disorder and schizophrenia in a cross-disorder genome wide analysis [15]. Cadhedrin, *CDH13*, is a known ADHD-susceptibility gene that has been investigated in other neuropsychiatric disorders [24], [25], [26]. Some of the other shared genes such as *ITIH4*, inter-alpha-trypsin inhibitor heavy chain family, member 4, and *PDE7B*, phosphodiesterase 7B do not have clearly known links to the neuropsychiatric phenotypes and might be interesting to explore further.

### “Clinical without observed genetic effect” results

In this section we report disease pairs that are significant in both EMRs, but not significant in VARIMED (“clinical without observed genetic effect”). One protective interaction was found: alcoholism and goiter. This pair in the Columbia dataset has an observed/expected ratio of 0.501 (*p* < 1.55 × 10^−22^); in the Stanford dataset, 0.297 (*p* < 6.16 × 10^−61^). Table 6 shows the 23 overrepresented interactions.

**Table 6:** Results for overrepresented disease pairs that are significant in Columbia and Stanford EMRs but not in VARIMED.

				Columbia		Stanford	
	Disease 1	Disease 2	Cluster name	Obs/Exp	P-value	Obs/Exp	P-value
1	Alcoholism	Bipolar disorder	adulthood	7.40	0.00E+00	3.32	1.55E-239
2	Alcoholism	Depression	adulthood	5.80	0.00E+00	2.80	1.28E-179
3	Alcoholism	HIV	adulthood	5.38	0.00E+00	1.89	6.96E-08
4	Alcoholism	Hepatitis B	adulthood	3.45	3.71E-138	1.56	9.53E-10
5	Alcoholism	Schizophrenia	adulthood	6.82	0.00E+00	3.93	1.74E-94
6	Alzheimer’s	Parkinson’s d.	aged	15.91	0.00E+00	6.95	8.46E-88
7	Aortic aneurysm	Cardiomyopathy	aged	4.54	3.14E-54	1.51	4.81E-10
8	Aortic aneurysm	Cholelithiasis	aged	2.73	3.65E-23	1.44	1.02E-07
9	Attention deficit	Autism	youth	31.86	6.52E-126	7.35	2.03E-172
10	Bipolar disorder	Migraine	adulthood	3.46	3.56E-86	1.69	2.58E-32
11	Cardiomyopathy	Diabetes type 2	aged	4.61	0.00E+00	1.29	2.89E-26
12	Cholelithiasis	Diabetes type 2	aged	3.04	0.00E+00	1.31	1.23E-29
13	Cholelithiasis	Hepatitis C	aged	3.19	5.65E-203	2.81	3.47E-101
14	Depression	HIV	adulthood	6.57	0.00E+00	1.72	1.44E-04
15	Depression	Migraine	adulthood	4.14	9.28E-286	2.09	1.27E-83
16	Depression	Schizophrenia	adulthood	11.74	0.00E+00	3.89	1.89E-66
17	Diabetes type 2	Gout	aged	3.44	3.32E-12	2.80	5.46E-11
18	Diabetes type 2	Hepatitis C	aged	4.92	0.00E+00	1.46	2.92E-48
19	Goiter	Tuberculosis	adulthood	1.66	1.17E-65	1.78	5.86E-05
20	HIV	Hepatitis B	adulthood	9.56	1.46E-213	4.67	1.76E-21
21	HIV	Tuberculosis	adulthood	6.03	0.00E+00	3.12	1.03E-03
22	Hepatitis B	Tuberculosis	adulthood	4.28	0.00E+00	4.07	2.83E-14
23	Migraine	Systemic lupus erythematosus	adulthood	3.96	5.45E-31	1.75	9.76E-12

Obs/Exp = Observed/Expected.

As expected, disorders with clear environmental triggers are apparent in both lists: alcohol, injection drug use (HIV, hepatitis B and C), and diet (diabetes type 2, and gout). Of the protective pairs, alcoholism and goiter have been previously noted to be underrepresented [27].

Most of the detected synergistic interactions are well-known. These include: alcoholism and bipolar disorder, alcoholism and depression, alcoholism and schizophrenia, depression and schizophrenia, and the alcoholism and injection drug-associated pairs. Of those less familiar, references are provided here: depression and migraine [28], migraine and lupus [29], cardiomyopathy and diabetes [30], aortic aneurysm and cardiomyopathy [31], diabetes type 2 and gout [32], attention deficit and autism [33]. The association between alzheimer’s and parkinsonism is very likely due to diagnostic overlap, given known differences in mechanism but difficulties in the clinical diagnosis of dementia subtypes. Lack of clear genetic signal for all these pairs does not completely rule out any genetic connection, as some disorders may have not yet been subject to scrutiny through GWAS studies, or have only modest effect sizes not reaching statistical significance.

### “Genetic without observed clinical effect” results

In this section we report disease pairs that have significant overlap of genetic variants in VARIMED, but are not significant in both EMRs. There are 17 such pairs, shown in Table 7; when the disease pair was significant in one EMR, that was recorded in the “EMR” column.

**Table 7:** Results for disease pairs that are significant in VARIMED after removing pairs that are significant in both Columbia and Stanford EMRs.

	Disease 1	Disease 2	Cluster name	Disease 1 genes	Disease 2 genes	Gene overlap	P-value	OR	EMR
1	Alopecia areata	Behcet’s s.	other	75	42	6	3.47E-08	38.73	
2	Alopecia areata	Celiac sprue	other	75	45	7	1.37E-09	42.79	
3	Alopecia areata	Diabetes type 1	other	75	323	40	4.36E-49	65.18	
4	Alzheimer’s	Diabetes type 2	aged	179	254	12	8.84E-06	5.24	C
5	Ankylosing spondylitis	HIV	adulthood	38	92	7	9.93E-10	45.80	
6	Ankylosing spondylitis	Multiple sclerosis	adulthood	38	261	10	1.08E-10	25.32	C
7	Behcet’s s.	Diabetes type 1	other	42	323	18	6.74E-21	42.80	
8	Celiac sprue	Diabetes type 1	other	45	323	16	3.46E-17	31.49	S
9	HIV	Multiple sclerosis	adulthood	92	261	26	4.00E-26	27.90	
10	HIV	Psoriasis	adulthood	92	104	15	1.74E-17	34.94	C
11	HIV	Systemic lupus erythematosus	adulthood	92	175	27	7.99E-32	44.09	
12	Multiple sclerosis	Psoriasis	adulthood	261	104	28	1.60E-25	21.56	C
13	Multiple sclerosis	Schizophrenia	adulthood	261	208	14	2.47E-06	5.06	C
14	Multiple sclerosis	Systemic lupus erythematosus	adulthood	261	175	47	1.92E-42	23.34	C
15	Parkinson’s d.	Rheumatoid arthritis	aged	151	348	15	2.22E-07	5.84	C
16	Psoriasis	Systemic lupus erythematosus	adulthood	104	175	26	1.06E-28	35.40	C
17	Schizophrenia	Systemic lupus erythematosus	adulthood	208	175	10	3.84E-05	5.37	

Disease 1/2 genes = number of genes for each disease in VARIMED,

Gene overlap = number of shared genes,

OR = Odds ratio,

EMR = which EMR had result,

C = Columbia,

S = Stanford.

The group of “genetic without observed clinical effect” are those which have significant genetic overlap in VARIMED, but not in both EMRs. Nearly all are autoimmune disorders, and may represent pairs with sharing detected at the level of genes that do not produce pathway interactions leading to disease phenotypes, or rare interactions that do not achieve statistical significance.

## Discussion

In this paper we present a method to identify statistically significant disease pairs which display significant comorbidity in two EMRs and share common genetic background in a large database of disease-associated variants; we explicitly model the under and overrepresentation of disease pairs, and control for the confounding effects of age on disease incidence by only comparing diseases when they fall in the same cluster of similarly-shaped incidence patterns. The method is fast, easy to interpret, and can be extended in a straightforward manner to other EMRs, data from national health systems, and large insurance databases.

Our primary aim is to identify disease pairs which might share a common mechanism or treatment option for further exploration and research. We link disease pairs that are under or overrepresented in EMR data to statistically significant overlapping genes sets for the same pairs. The genetic variants are known to have phenotypic effects, while EMRs capture a broad collection of diseases states that are severe enough to require diagnosis and treatment, and represent a constellation of genetic predispositions, environmental influences, and social and economic factors that affect when diseases are detected. Many of the predisposing factors in EMRs are not measured, but we can find pairs that have known genetic associations and also find pairs that do not. As always for candidate generation or prioritization methods, the question arises of how to validate novel results, given that validating experiments have not yet been conducted. By contrasting results in two EMRs and in a database of genetic variants, we have reduced the chance that the same biases are operating across all data sources.

There are several limitations of our approach which should be recognized. Our method only compares diseases when they fall in the same cluster. This is a simple, but conservative, match on age patterns, and should enrich results for true positives at the expense of missing other true positives that would only be found in cross-cluster comparisons. For example, because autism and Alzheimer’s disease would fall in different age-incidence clusters and would not be compared, possible interactions between those disorders would not be detected. VARIMED, while large, contains only published results, reflecting investigators’ choices of important areas of study, including, as we found, autoimmune disorders and neuropsychiatric disorders. Also, VARIMED focuses primarily on common variation as most genetic association has been based on genotype-based GWAS. VARIMED (and other databases) are not randomly sampled from the space of biological phenomena, and the absence of a genetic variant may only mean that such have not yet been investigated. It is likely that our method will fail in such circumstances to identify comorbid pairs using the conjunction of data from EMRs and from VARIMED, which is a source of bias. In addition, we link to EMR records on the basis of a straightforward, but necessarily imprecise, mapping through a disease name. We restrict the genetic analysis to the genes and do not consider the allele-specific relationships (risk-enhancing or risk-moderating). Although both gender and ethnicity are known to be important covariates for the prevalence of disease, because the available Columbia data were not stratified by gender or ethnicity, neither were used in this study. This would be particularly important for autoimmune disorders with their known gender dependence; combining the genders for analysis, as we had to do, may have diluted statistical signal, and would explain the appearance of results in Table 7. Finally, the set of diseases examined was restricted to the 161 in the original Rzhetsky study, and further restricted by the limited overlap with VARIMED; although drawing from both common and rare diseases, the set is small compared to the full range coded by ICD9. Prior studies have found multiple protective interactions between CNS disorders and cancers [34] but, unfortunately, few cancers were in the list of disorders analyzed here. In spite of these limitations, we hope this study can serve as a proof of principle for integrating EMR and genetics data to uncover relationships between diseases. Using larger data sets, and incorporating important covariates and the direction of allele-specific risk would important validating extensions of the current work. Furthermore, text mining of EMR clinical notes and other databases of environmental exposures could represent an opportunity for identifying non-genetic causes of diseases.

In conclusion, we have presented a method integrating clinical EMR and genetics data in order to elucidate disease comorbidity. We identify a set of disease pairs which deviate from the independence assumption in their co-occurrence in two different EMR systems. By integrating the clinical observations with genetics, we are further able to categorize which of the disease pairs might be explained by the shared genetics and which might have more of an environmental component.

## Materials and Methods

### Ethics statement

Our validation data set used patient records from Stanford’s electronic medical record system, STRIDE (Stanford Translational Research Integrated Database Environment). The data request was judged to be exempt from human subject concerns by the Stanford Institutional Review Board, and was also approved by its Data Privacy Office. The Stanford data were retrieved June 6, 2013. Encounter records contained a masked patient identifier, current age, gender, ethnicity, icd9, and age at visit. (Gender was not used because gender information was not generally available for the Columbia data. Ethnicity was not used for the same reason.) Because of small numbers of very old patients, ages were censored at 90 years for privacy reasons by STRIDE staff prior to our use.

### Clinical data analysis

For the electronic medical record data, the discovery data set comes from the composite data for the Columbia EMR, published as an online appendix of [2], which lists counts of diseases and disease pairs for a total of 161 disorders. As described in the original article, “We selected disorders that represent a broad spectrum of maladies, from common to rare, affecting diverse physiological systems, yet we also placed special emphasis on neurological phenotypes.”

The total number of patients was 1,478,976, however because these records include data on healthy hospital employees, the total was lowered by 500,000 as described in their Appendix 2, p 19. Disease count data were extracted from their Appendix 3, and disease-pair count data were extracted from Supplemental Information Data Set 1. Separately, the mapping from ICD9 codes to disease names was taken from their Appendix 3; a small number of mapping errors were corrected by hand.

For validation, patient-level data were retrieved from STRIDE (Stanford Translational Research Integrated Database Environment) [35] for the same 161 diseases to allow for direct comparison with the Columbia data. The raw data contained 1,057,132 records for 397,474 patients. We focus our analysis on data starting in the year 2008, which was the year of comprehensive EMR rollout. When there were fewer than 50 patients with a disease, that disease was judged too rare to contribute to the incidence frequencies in a meaningful way, and was removed. This left data for 277,290 patients. Also, disease pairs were removed if any of the cells in the 2 × 2 table had observed or expected values less than 5.

The EMR records were aggregrated and processed to retain the earliest occurrence of each ICD9 code for each patient, which were then consolidated using the ICD9-to-disease mapping from [2] to produce a table of patient counts for each disease name. A similar procedure was used to count disease pairs.

### Correcting for age bias through clustering

**Fig 5:**
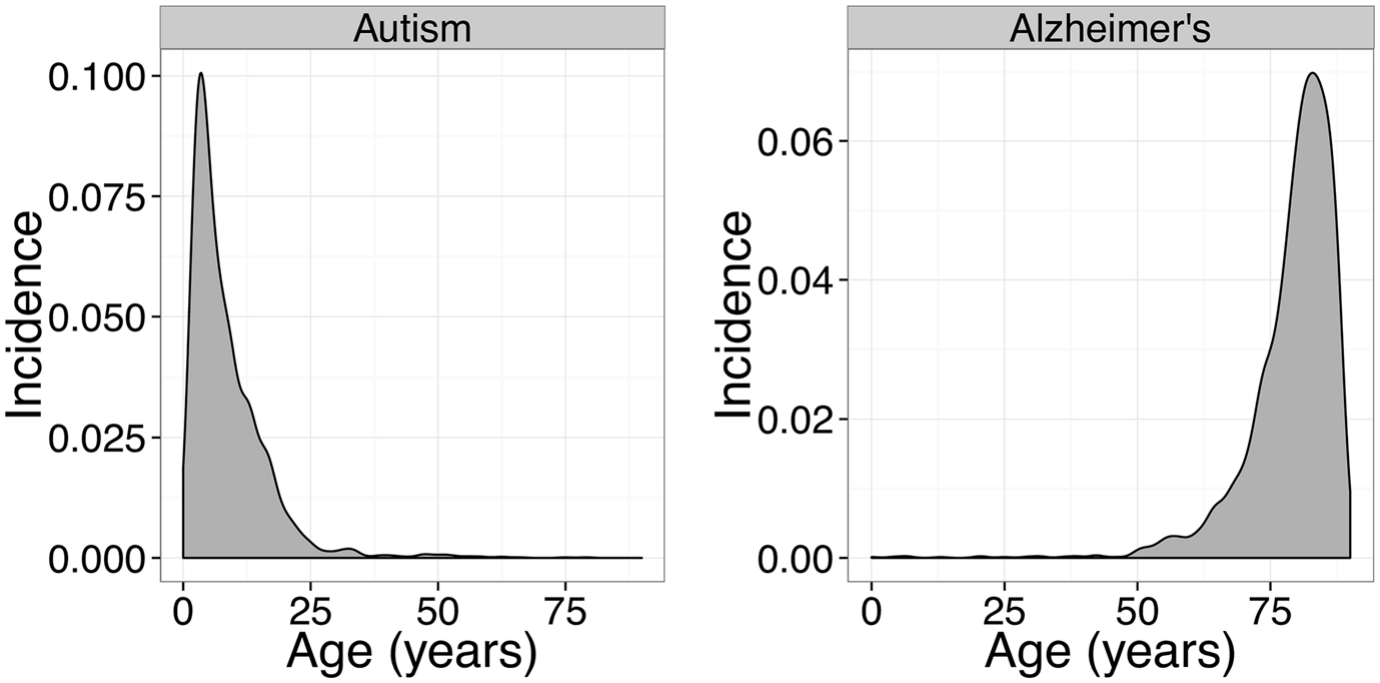
Incidence-by-age graphs for autism and Alzheimer’s disease. Because of the gross disparities in these patterns, patients at risk for one disorder would be a low risk for the second disorder at any given age, reducing the observed comorbidity.

Biases arise from EMR data not being a random sample of diseases in the population. For example, autism and Alzheimer’s disease have very different incidence patterns. (See Fig 5.) It would be unlikely for a patient to have this disease pair in their records, even if they were ultimately afflicted by both disorders, because young patients at risk for autism would not also be at risk for Alzheimer’s until many years in the future, and those at risk for Alzheimer’s would have been at risk for autism in an era when the EMR did not exist, even if autism had then been a clearly defined syndrome. This will lead to systematic undercounting of these and similar disorder pairs.

To control for the confounding effects of age, disease pairs were analyzed only when both diseases could be put in the same age-incidence cluster. Clusters were formed so as to be as large as possible (to maximize the number of subsequent disease comparisons), while simultaneously imposing within-cluster homogeneity, so that each cluster had similar age-incidence patterns. The age-incidence clustering used from patient-level data for Stanford; corresponding details for the Columbia data were not available.

Each disease was represented as a 91-dimensional vector containing counts of the number of patients whose earliest onset of that disease occurred for each of the ages 0 years through 90 years. For normalization, each vector was divided by its length to produce unit vectors. Hierarchical clustering with Ward’s method for linkage was chosen to produce clusters that were compact and of similar size.

Cluster size was determined in a data-driven manner by systemically searching through possible clustering methods and cluster scoring measures. The methods and measures were taken from those provided by the R package COMMUNAL and are listed in the Supplemental Material. The cluster measures (also known as cluster indices) provide scores for each method and each cluster size. The measures were combined into a composite score by standardizing each measure (zero mean, unit variance) and then averaging. All measures were converted to have the same sense, so that larger values were associated with more desirable clusters. Any measure with a monotonic function (either increasing or decreasing) of cluster size for all methods and measures was removed because such a measure would be minimized or maximized at the extremes of the search range for cluster size, and thus not be responsive to patterns in the data.

Pairs of diseases that showed significant comorbidity pairs were identified in the Columbia data and verified in the Stanford data, so all pairs reported here were statistically significant in both. In addition, only pairs that were underrepresented in both EMRs or overrepresented in both EMRs were retained, ensuring consistent directionality; discordant pairs were not analyzed. Statistical significance was computed using the Fisher exact test. Bonferroni correction was applied using the number of diseases in each cluster. The conventional level of significance, 0.05, was used for all tests.

### Genetic analysis

Genetic associations came from VARIMED, a hand-curated database of published phenotype-associated genetic variants [3]. As of May, 2015, this database contained variants from 17,088 publications, with 466,890 SNPs associated with 2,992 diseases or traits. SNPs were mapped to genes using the dbSNP annotation database. Sometimes, a variant maps to more than one gene. In such cases, we use colons to separate the genes in a single group; this notation is used in Table 5. Phenotype descriptions were mapped by hand to the set of 161 disease names used for the Columbia and Stanford data. There were 35 diseases that appeared in Columbia, Stanford, and in VARIMED (Table 3). Because VARIMED is proprietary, the relevant subset of 35 diseases, with associated genes, chromosome number, and PubMed ID of the source of each association were extracted and used for the analysis we report here. This dataset is included in the Supplement.

Genetic variants that were significantly associated with each phenotype of interest were obtained from VARIMED and mapped to gene names. In this study, we used significant disease-SNP associations (*p* < 10^−6^) with known risk alleles and published odds ratios. The number of genes associated with each of the 35 diseases of interest are shown in Table 3. We furthermore focus our analysis on the gene level, specifically calculating enrichment of the number of overlapping genes between two phenotypes of interest. We report the number of genes shared by the disease pair if the overlap was determined as significant by the Fisher exact test using Bonferroni correction for the number of tests.

A network diagram (Fig 3) showing the structure of the disease pairs and their clusterings was created using the Cytoscape software tool. In the network diagram, a node represents a disease. Two nodes are connected if that disease pair is statistically significant in the EMR data and appears in the same age-incidence cluster. The size of a node represents the frequency of that disease in the larger EMR (Columbia). The edge width represents the effect size for that pair (observed number divided by expected number). The node color indicates cluster membership, using the same colors as in Fig 2.

## Supporting Information

S1 Appendix Contains information about the Columbia and Stanford EMR data sets, and details on the age-incidence clustering method. (PDF)

S1 File Subset of VARIMED used in our analysis, with disease name, gene name, chromosome, and PubMed ID. (CSV)

S2 File Diseases and disease clusters. (CSV)

S3 File Disease pairs from Columbia EMR with statistically significant under or overrepresentation. (CSV)

S4 File Disease pairs from Stanford EMR with statistically significant under or overrepresentation. (CSV)

S5 File Disease pairs in intersection of Columbia and Stanford EMRs. (CSV)

S6 File Disease pairs from VARIMED with statistically significant gene overlap. (CSV)
