# Supplementary material for: Constraints on Biological Mechanism from Disease Comorbidity Using Electronic Medical Records and Database of Genetic Variants
Source: PLoS Comput Biol. 2016 Apr 26;12(4):e1004885. doi: 10.1371/journal.pcbi.1004885 (PMC4846031; doi:10.1371/journal.pcbi.1004885)
Supplement: S1 Appendix — (PDF) [file pcbi.1004885.s001.pdf]

Appendix for "Constraints on biological mechanism from disease comorbidity using electronic medical records and database of genetic variants"

Bagley, Sirota, Chen, Butte, and Altman

This appendix contains additional information about the Columbia and Stanford EMR data sets. It also contains details on the age-incidence clustering method, and extended results for the search over cluster size.

1 Columbia data set

All of the data were extracted from the supporting information available on-line at `http://www.pnas.org/content/104/28/11694/suppl/DC1`.

The paper refers to "1.5 million patient records" (which is ambiguous about whether it is 1.5M patients or 1.5M records from some smaller number of patients; it appears to be the former). The exact number used here can be computed from the counts in the NeuroSkin.txt file obtained from Andrey Rzhetsky. That count is 1,478,976 patients. According to Rzhetsky Appendix 2, p 19, this count included data on healthy hospital employees. The count was therefore corrected by subtracting their estimate of the number of healthy employees (500,000) to produce 978,976.

A disease pair was removed if any of the expected or actually cell counts were less than 5. This removed 8,886 pairs, leaving 3,834 disease pairs.

2 Stanford data set

The Stanford (STRIDE) data set contains 1,057,131 records. Records were removed if from a year before 2008, if the age was 90 or greater, if the record were internally inconsistent (e.g, if an age at diagnosis was greater than the age at time of data retrieval, or if the patient was born in a future year),

or if the number of patients with a given disease was less than 50. This left 777,679 records.

A disease pair was removed if any of the expected or actually cell counts were less than 5. This removed 1554 pairs, leaving 2932 disease pairs.

3 Main script (as pseudocode)

```
Read Columbia disease counts and disease pair counts
Compute disease frequencies
Compute measure of statistical significance (fisher exact) for each pair

Read STRIDE records
Convert ICD9 codes to disease names
Remove gender specific diagnoses (Breast cancer F/M)
Compute disease counts
Compute disease frequencies
Compute disease pair counts
Compute measure of statistical significance (fisher exact) for each pair

Remove small cells from pairs data
Record number of disease pairs for Columbia and STRIDE

Compute for each patient and each disease the year and age of disease onset

Compute the optimal cluster size (described in paper Methods)

For each possible cluster size, k:
    Compute the k clusters
    Write out the cluster pdfs

Set cluster size to 5 (based on above)

Read VARIMED disease pairs and gene lists

Make Bonferroni correction to VARIMED p value threshold

Remove all pairs when disease name not in cluster list, both diseases
are not in the same cluster, and pair is not statistically significant
```

```
Form set overlaps:
    Columbia + STRIDE
    Columbia + STRIDE + VARIMED (separately for over/under represented)
    VARIMED - Columbia - STRIDE

Create Venn diagram

Write out the overlap tables
```

4 Output log files

Two log files are produced by each run. They contain additional information about the results of stages in the analysis pipeline, especially the size of data objects produced.

```
/Users/sbagley/Sync/conte/emrvarimed/results/log-20150917-110029.txt

Log file: ../results/log-20150917-110029.txt




Reading Columbia data.

Columbia: starting with 161 diseases for 978976 patients

Columbia: starting with 12720 disease pairs for 978976 patients




Reading STRIDE data.

Removed 278271 old records, now have 778860 records


Removed 114 inconsistent records, now have 778746 records


Removed 1067 rare disease records, now have 777679 records
```

Rare diseases:

```
rare[order(-N)]=
                            disease_name  N
 1:                         Cervical rib 49
 2:                  Neuromyelitis optica 49
 3:                    E. coli intestinal 48
 4:       Primary cerebellar degeneration 48
 5:                         CNS viral d. 47
 6:                            Amebiasis 41
 7:                           Patau's s. 41
 8:                             Aciduria 37
 9:        Congenital absence of vertebra 37
10:                             Aniridia 36
11:                         Meningococcus 36
12:                   AA metabolism (Lowe) 32
13:                             Shigella 32
14:                      Goodpasture's s. 26
15:                      Renal glycosuria 25
16:                          Hepatitis D 23
17:          Acute promyelocytic leukemia 22
18: Polyostotic fibrous dysplasia of bone 22
19:  Enzyme-deficiency (hemolytic anemia) 21
20:                     Hodgkin's disease 21
21:                                Mumps 20
22:         Multiple epiphyseal dysplasia 19
23:                          Brucellosis 16
24:                Lown-Ganong-Levine s. 15
25:                                Prion 15
26:                    Myotonic disorders 14
27:                          Hepatitis E 12
28:                    Friedreich's ataxia 11
29:                         Schilder's s. 11
30:         Erythematosquamous dermatosis  8
31:                               Plague  8
32:                           Ornithosis  6
33:                            Tularemia  5
```

```
34:                        Acute leukemia  3
35:                                Ainhum  3
36:                               Anthrax  3
37:                               Leprosy  3
38:                  Plasma cell leukemia  2
39:                               Cholera  1
40:              Lethal midline granuloma  1
                             disease_name  N
Removing gender specific diagnoses (Breast cancer F and M)

STRIDE: starting with 120 diseases for 277290 patients

STRIDE: starting with 4486 disease pairs for 277290 patients

Removing all pairs with small counts (<5):

Removing 8886 rows from columbia_pairs

leaving 3834 disease pairs.

Removing 1554 rows from stride_pairs

leaving 2932 disease pairs

Computing optimal cluster size.


run_communal:

cluster_range 2 9

using clustering methods: hierarchical kmeans diana som model sota
pam clara agnes

aggl.method = ward

using 13 nonmonotonic measures: Connectivity avg.silwidth ch dunn
dunn2 g2 g3 max.diameter min.separation pearsongamma sindex wb.ratio
widestgap
```

```
communal avg:

avg=
   cluster composite_score
1:       2     0.570401978
2:       3    -0.207070445
3:       4    -0.134285533
4:       5     0.039038832
5:       6    -0.187851664
6:       7    -0.031883969
7:       8    -0.051998578
8:       9     0.003649379
optimum cluster size: 5


run_communal:

cluster_range 2 9

using clustering methods: hierarchical kmeans diana som model sota
pam clara agnes

aggl.method = complete

using 14 nonmonotonic measures: Connectivity average.between
avg.silwidth ch dunn dunn2 g2 g3 max.diameter min.separation
pearsongamma sindex wb.ratio widestgap

communal avg:

avg=
   cluster composite_score
1:       2     0.532244278
2:       3    -0.268871940
3:       4    -0.194610298
4:       5     0.053250091
5:       6     0.008185307
6:       7    -0.082756403
7:       8    -0.083063569
8:       9     0.035622533
```

```
optimum cluster size: 5


run_communal:

cluster_range 2 9

using clustering methods: hierarchical kmeans diana som model sota
pam clara agnes

aggl.method = average

using 13 nonmonotonic measures: Connectivity avg.silwidth ch dunn
dunn2 g2 g3 max.diameter min.separation pearsongamma sindex wb.ratio
widestgap

communal avg:

avg=
   cluster composite_score
1:       2      0.44562457
2:       3     -0.11411345
3:       4     -0.19756781
4:       5      0.11410463
5:       6     -0.06396651
6:       7     -0.05184507
7:       8     -0.05598991
8:       9     -0.07624644
optimum cluster size: 5


Plotting data for each cluster size.


/Users/sbagley/Sync/conte/emrvarimed/results/k5/log-20150917-110350.txt

Log file: ../results/k5/log-20150917-110350.txt
```

```
Using cluster size of 5

Cluster sizes:

unique(clusters[, .(cluster_name, cluster_n)])=
   cluster_name cluster_n
1:      neonate        31
2:    adulthood        29
3:        youth        14
4:         aged        31
5:        other        15
Reading VARIMED

VARIMED: starting with 1560 disease pairs

p values:

overall p value threshold: 0.05

VARIMED p value threshold: 6.41025641025641e-05

Set sizes:

cun: 7 con: 515 sun: 87 son: 261 csun: 4 cson: 186 csuvn: 0 csovn: 5


Writing Venn diagram.

Writing csv tables for paper.

Done.
```

5 Clustering

Disease pairs are only compared when both diseases fall in the same age-incidence cluster. These clusters are formed from the vector of patient counts

(the count at each age from 0 to 90 years), normalized to unit length. The question arises of how to pick the number of clusters.

The R package COMMUNAL[1] was used for this purpose. It provides a way to systematically explore the effect on cluster size of choices of clustering methods (e.g, k-means) and clustering measures (e.g., silhouette width). Following ideas in COMMUNAL, we computed an average measure by first scaling each measure across possible cluster sizes to have zero mean and unit variance, and then averaging all the standardized measures across clustering methods, producing a composite score for each cluster size. All measures were converted to have the same sense (large values for better clusters) before standardizing. Any measure that was consistently increasing or consistently decreasing across the range of cluster sizes was removed; such measures would be maximized at the extremes of the search range for the cluster size, and would therefore not be responsive to patterns in the data.

The list of clustering methods (by name of R function) is: "hierarchical," "kmeans," "diana," "som," "model," "sota," "pam," "clara," "agnes."

The list of clustering measures (by name of R function) is: "Connectivity," "Average.between," "average.within," "avg.silwidth," "ch," "dunn," "dunn2," "entropy," "g2," "g3," "max.diameter," "min.separation," "pearsongamma," "sindex," "wb.ratio," "widestgap," "within.cluster.ss."

Clusters sizes from 2 to 9 were examined, 2 representing the small possible number of clusters (above no clustering at all), and 9 was chosen as an upper limit because it would produce, on average, clusters of size $161/9 = 17.9$, which was judged to be too small to produce enough disease pair comparisons. This range was later found to include local maxima of meaningful size.

Graphs of the composite index vs cluster size are shown for (1) ward linkage, (2) average linkage, (3) complete linkage.

[1] `http://cran.r-project.org/web/packages/COMMUNAL/COMMUNAL.pdf`

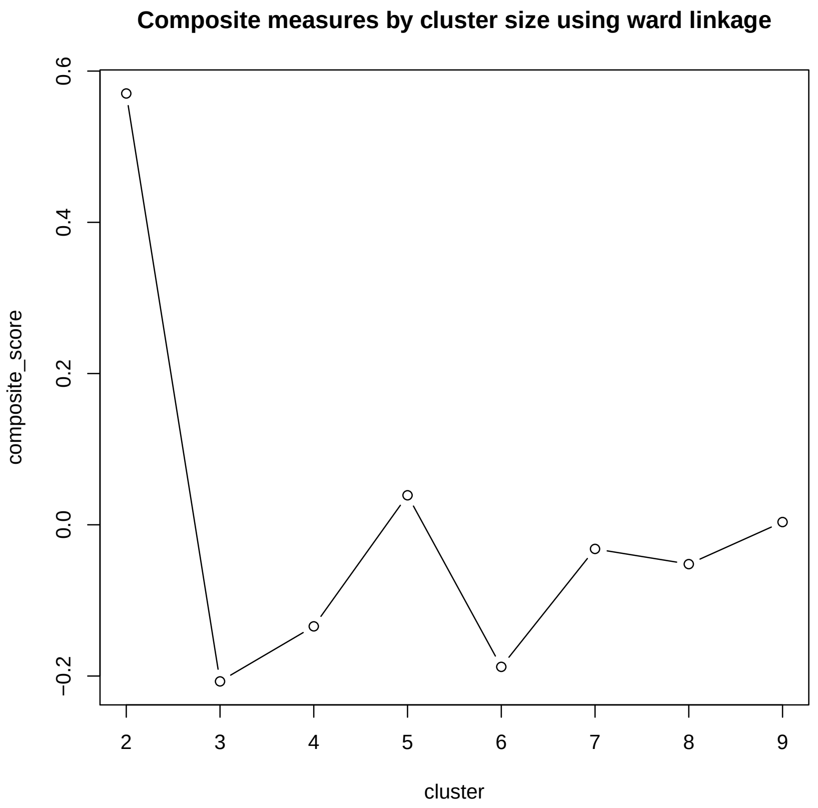
Composite measures by cluster size using ward linkage
composite_score
0.6
0.4
0.2
0.0
−0.2
2
3
4
5
6
7
8
9
cluster

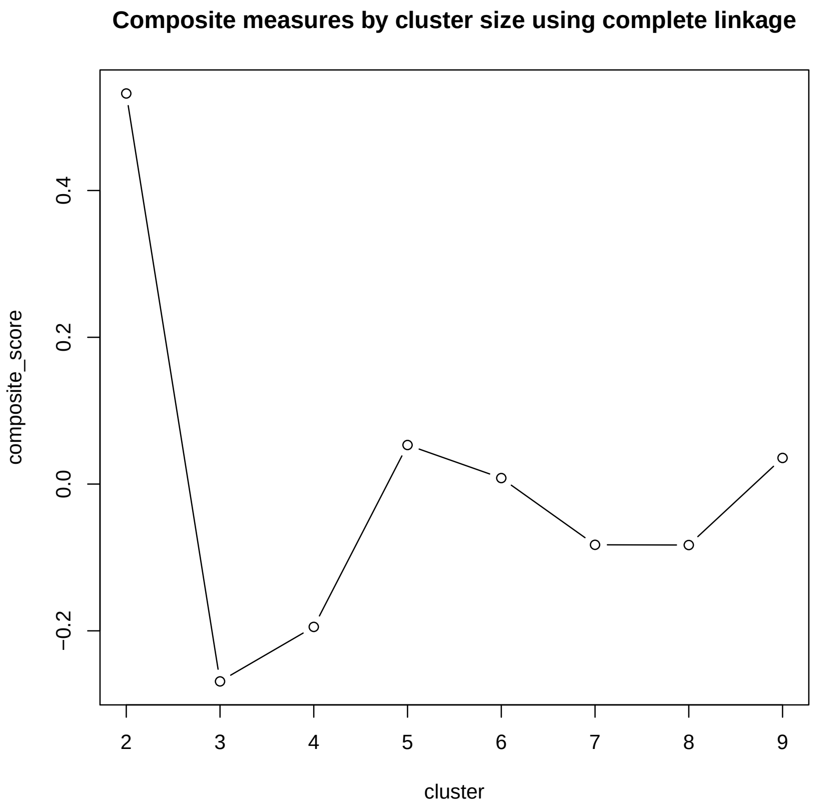
Composite measures by cluster size using complete linkage
composite_score
0.4
0.2
0.0
−0.2
2
3
4
5
6
7
8
9
cluster

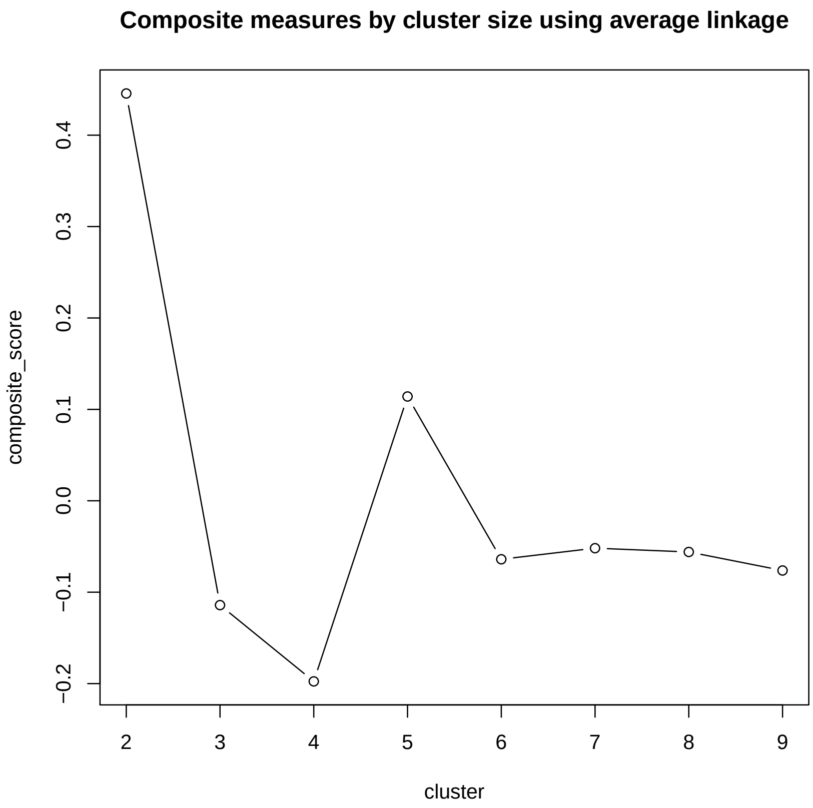

Ward, average, and complete linkage all have local maxima at cluster size 5, which was therefore used for our analysis.

Graphs for all clusters sizes in the range 2 to 9 are displayed here; the graphs show (1) all the points in each cluster, (2) the averages in each cluster, (3) density plots by disease for each disease in the cluster.

5.1 cluster size 2

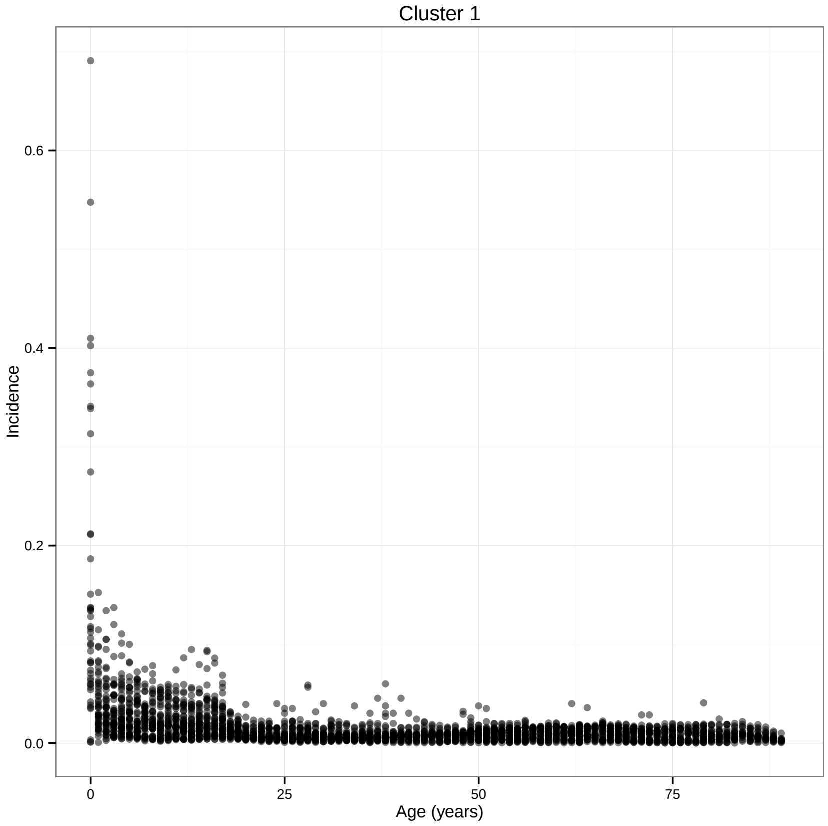

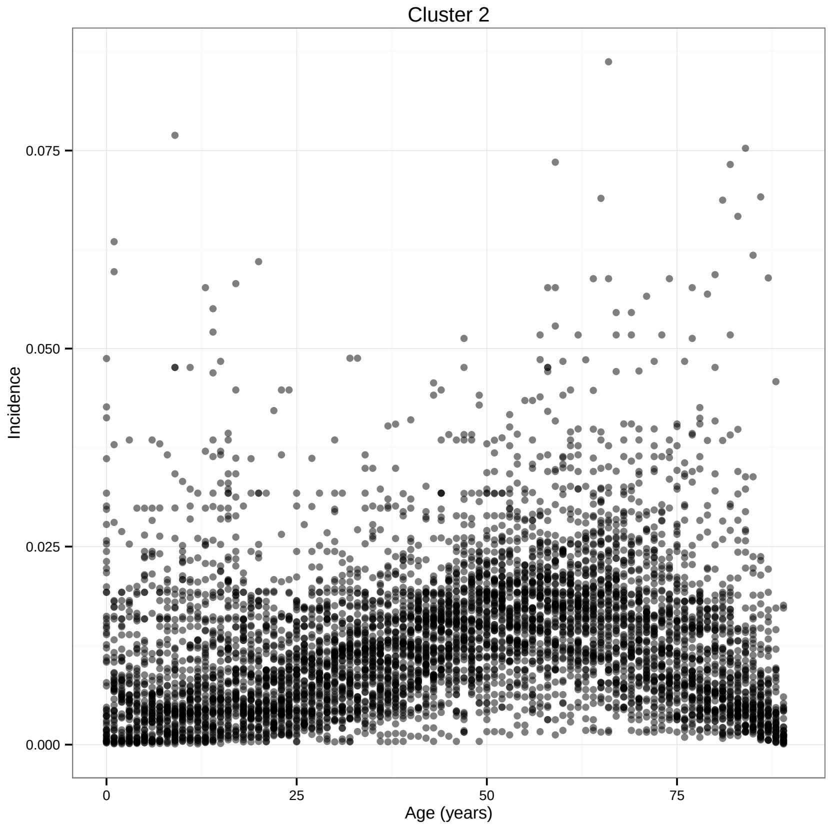
Cluster 2
Incidence
0.075
0.050
0.025
0.000
0
25
50
75
Age (years)

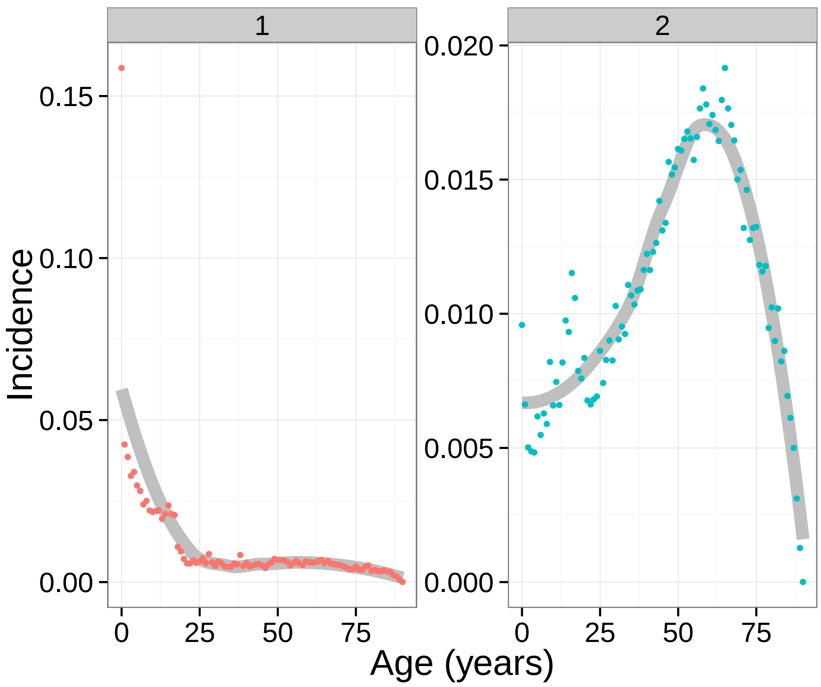
1
2
Incidence
0.15
0.10
0.05
0.00
0.020
0.015
0.010
0.005
0.000
0
25
50
75
Age (years)

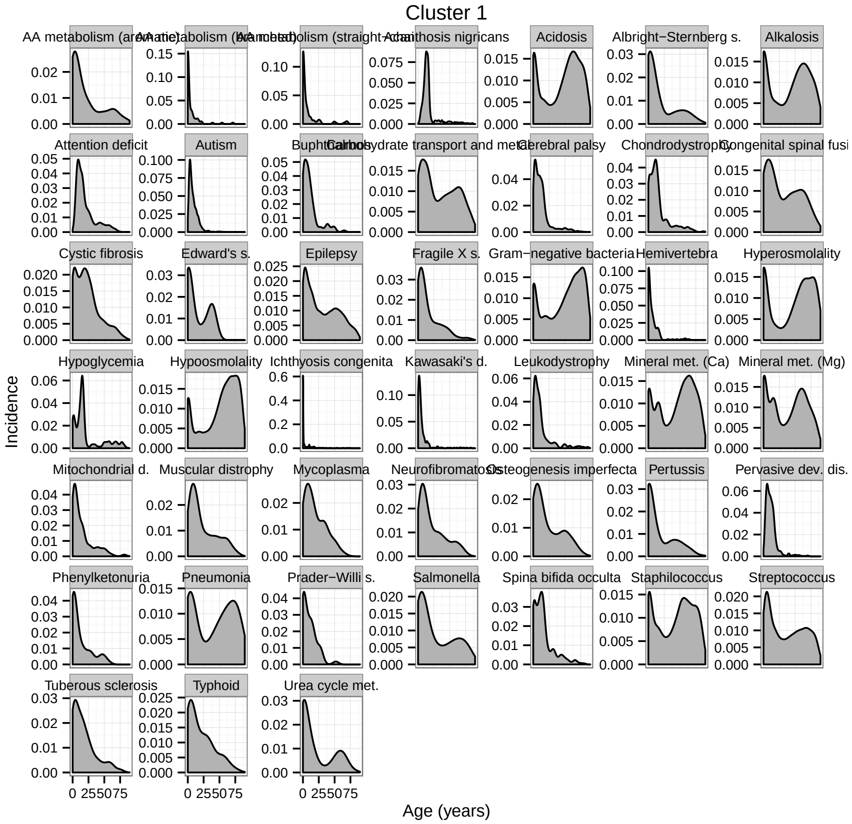
Cluster 1
AA metabolism (aromatic)
Acanthosis nigricans
Acidosis
Albright−Sternberg s.
Alkalosis
Attention deficit
Autism
Cerebral palsy
Chondrodystrophy
Cystic fibrosis
Edward's s.
Epilepsy
Fragile X s.
Gram−negative bacteria
Hemivertebra
Hyperosmolality
Hypoglycemia
Hypoosmolality
Ichthyosis congenita
Kawasaki's d.
Leukodystrophy
Mineral met. (Ca)
Mineral met. (Mg)
Mitochondrial d.
Muscular distrophy
Mycoplasma
Osteogenesis imperfecta
Pertussis
Pervasive dev. dis.
Phenylketonuria
Pneumonia
Prader−Willi s.
Salmonella
Spina bifida occulta
Staphilococcus
Streptococcus
Tuberous sclerosis
Typhoid
Urea cycle met.
Incidence
Age (years)

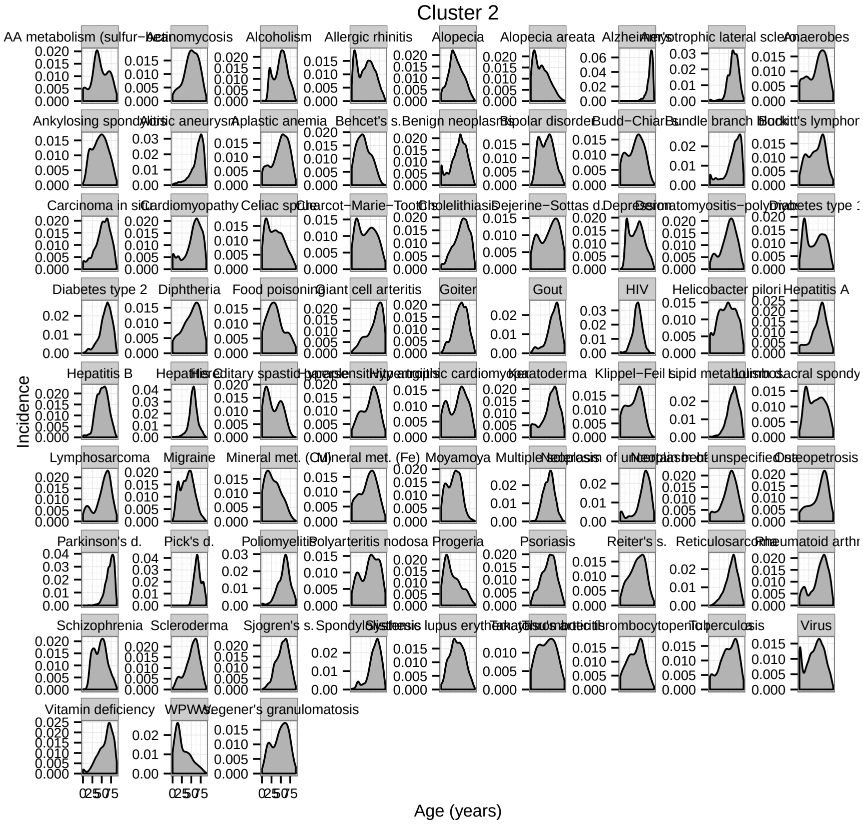
Cluster 2
Alcoholism
Allergic rhinitis
Alopecia
Alopecia areata
Anaerobes
Aplastic anemia
Bipolar disorder
Cardiomyopathy
Cholelithiasis
Diabetes type 2
Diphtheria
Goiter
Gout
HIV
Helicobacter pilori
Hepatitis A
Hepatitis B
Keratoderma
Lymphosarcoma
Migraine
Moyamoya
Osteopetrosis
Parkinson's d.
Pick's d.
Polyarteritis nodosa
Progeria
Psoriasis
Reiter's s.
Schizophrenia
Scleroderma
Sjogren's s.
Tuberculosis
Virus
Vitamin deficiency
WPW
Wegener's granulomatosis
Incidence
Age (years)

5.2 cluster size 3

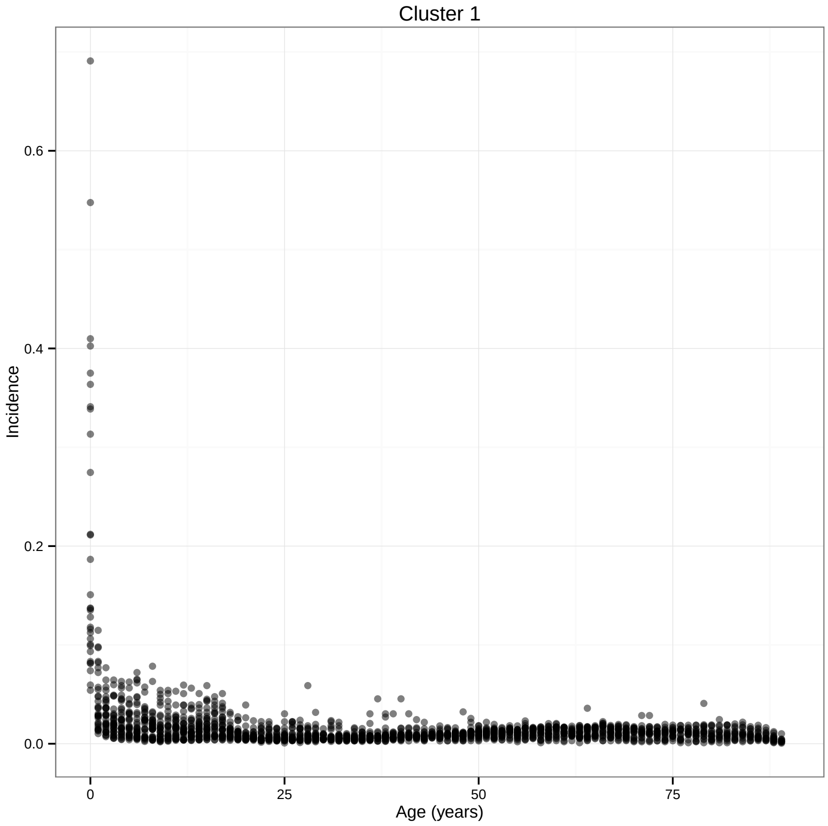

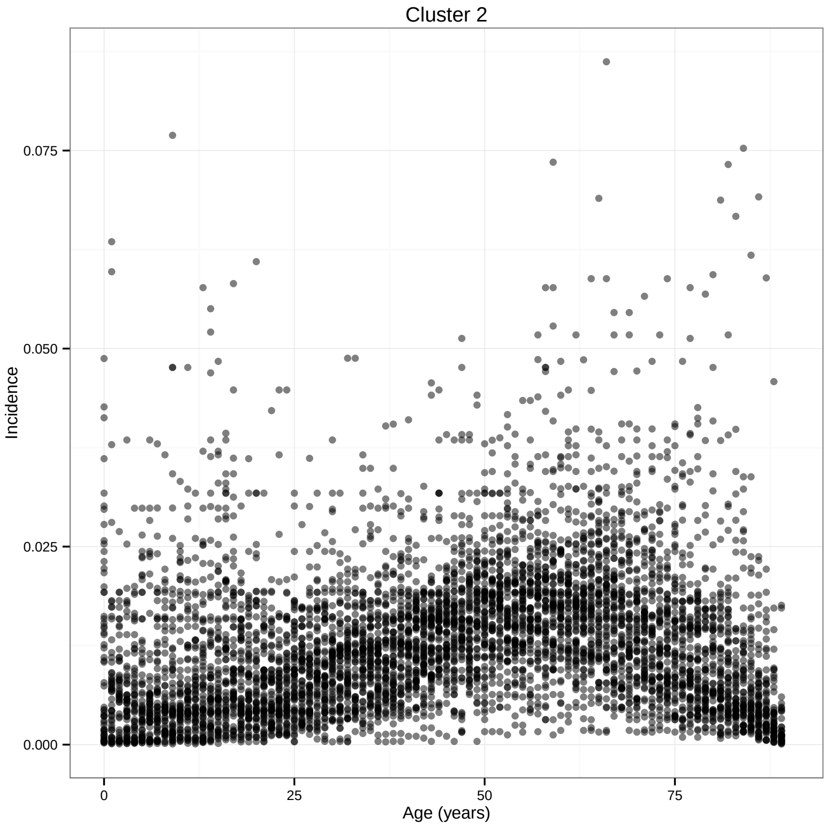

Cluster 2
Incidence
0.075
0.050
0.025
0.000
0
25
50
75
Age (years)

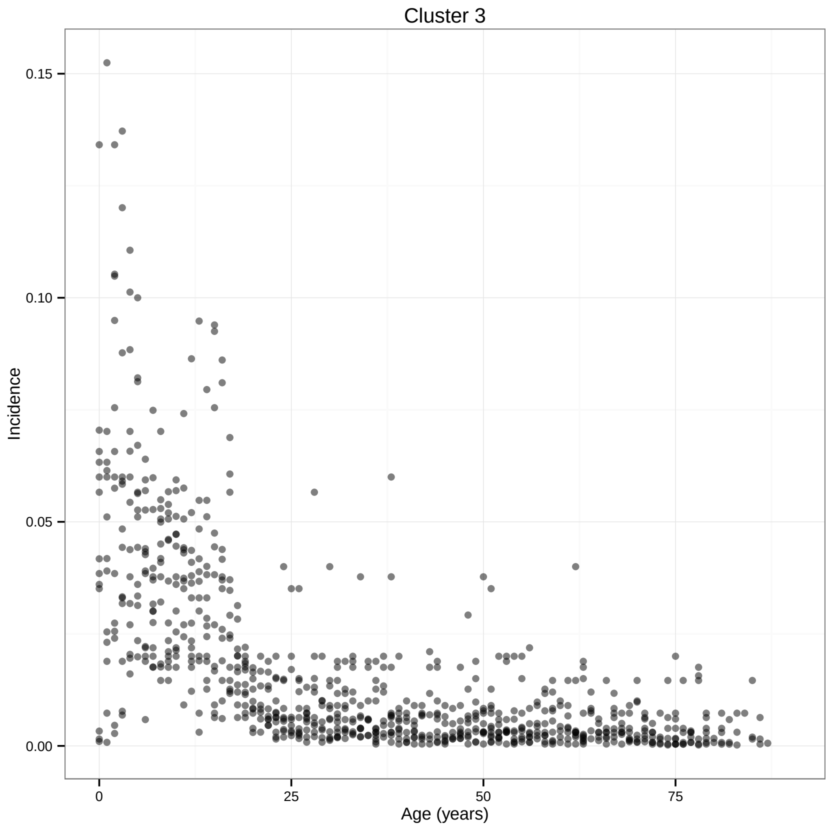
Cluster 3
Incidence
0.15
0.10
0.05
0.00
0
25
50
75
Age (years)

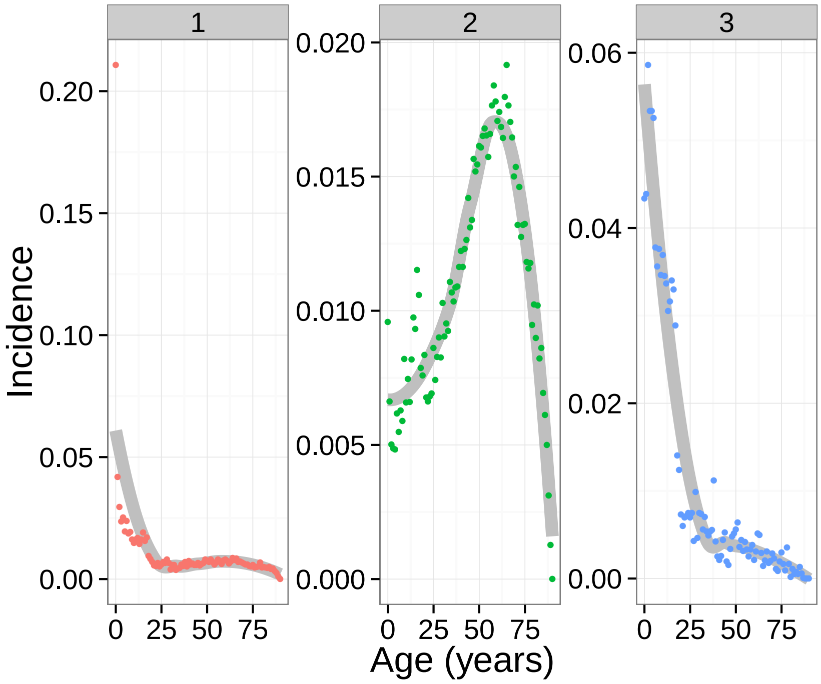
1
2
3
Incidence
0.00
0.05
0.10
0.15
0.20
0.000
0.005
0.010
0.015
0.020
0.00
0.02
0.04
0.06
0
25
50
75
Age (years)

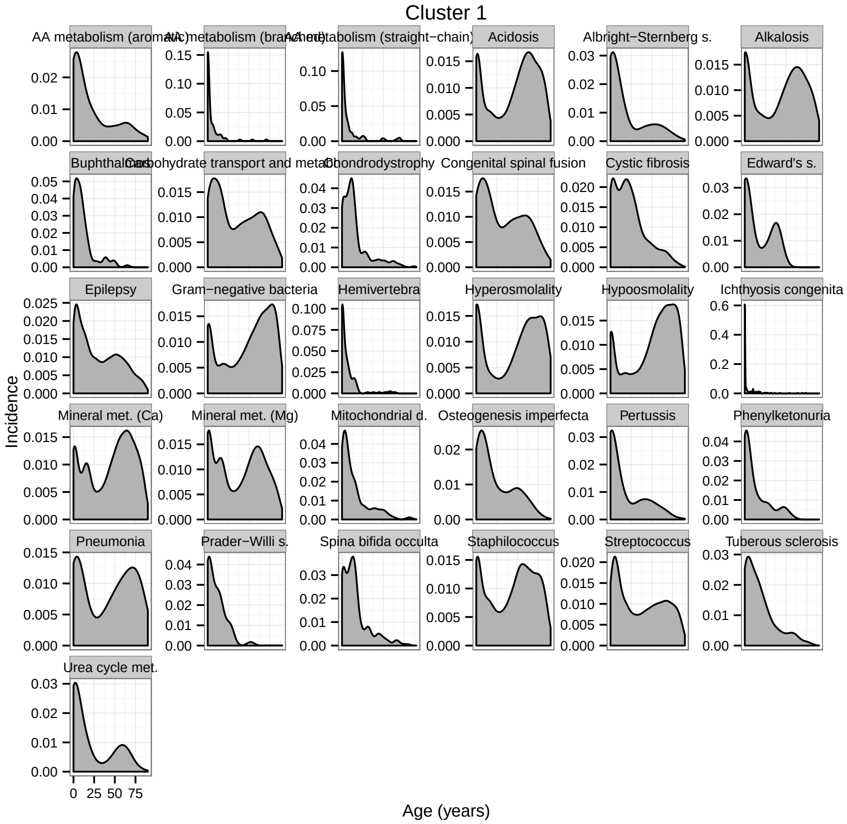
Cluster 1
AA metabolism (aromatic)
AA metabolism (branched)
AA metabolism (straight−chain)
Acidosis
Albright−Sternberg s.
Alkalosis
Buphthalmos
Carbohydrate transport and metab.
Chondrodystrophy
Congenital spinal fusion
Cystic fibrosis
Edward's s.
Epilepsy
Gram−negative bacteria
Hemivertebra
Hyperosmolality
Hypoosmolality
Ichthyosis congenita
Mineral met. (Ca)
Mineral met. (Mg)
Mitochondrial d.
Osteogenesis imperfecta
Pertussis
Phenylketonuria
Pneumonia
Prader−Willi s.
Spina bifida occulta
Staphilococcus
Streptococcus
Tuberous sclerosis
Urea cycle met.
Incidence
Age (years)
0 25 50 75

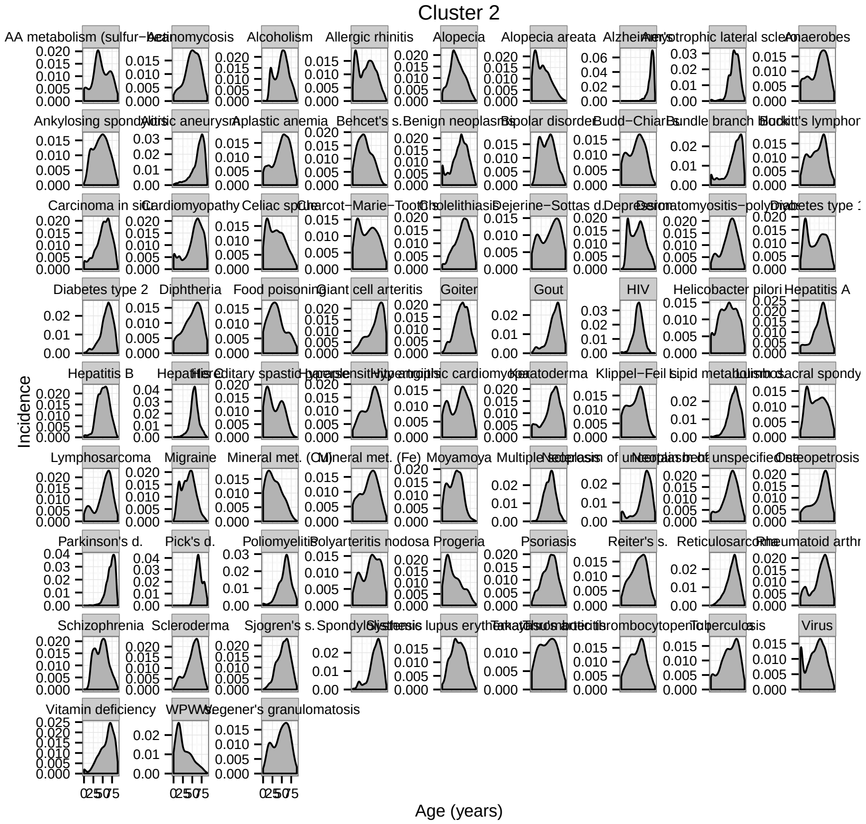

Cluster 2
Incidence
Age (years)

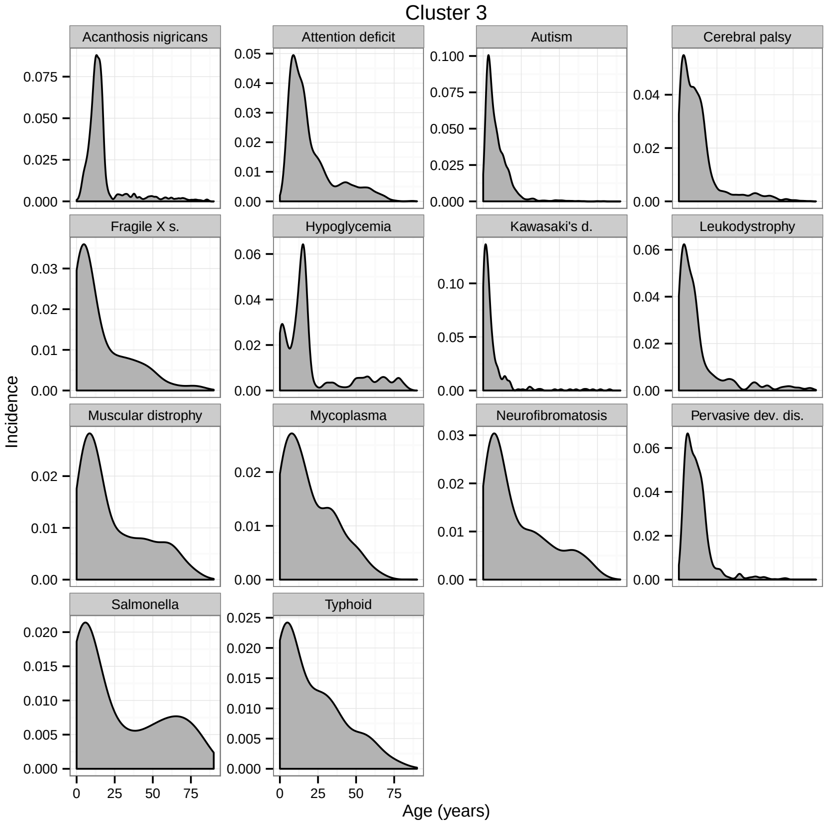
Cluster 3
Acanthosis nigricans
Attention deficit
Autism
Cerebral palsy
Fragile X s.
Hypoglycemia
Kawasaki's d.
Leukodystrophy
Muscular distrophy
Mycoplasma
Neurofibromatosis
Pervasive dev. dis.
Salmonella
Typhoid
Incidence
Age (years)

5.3 cluster size 4

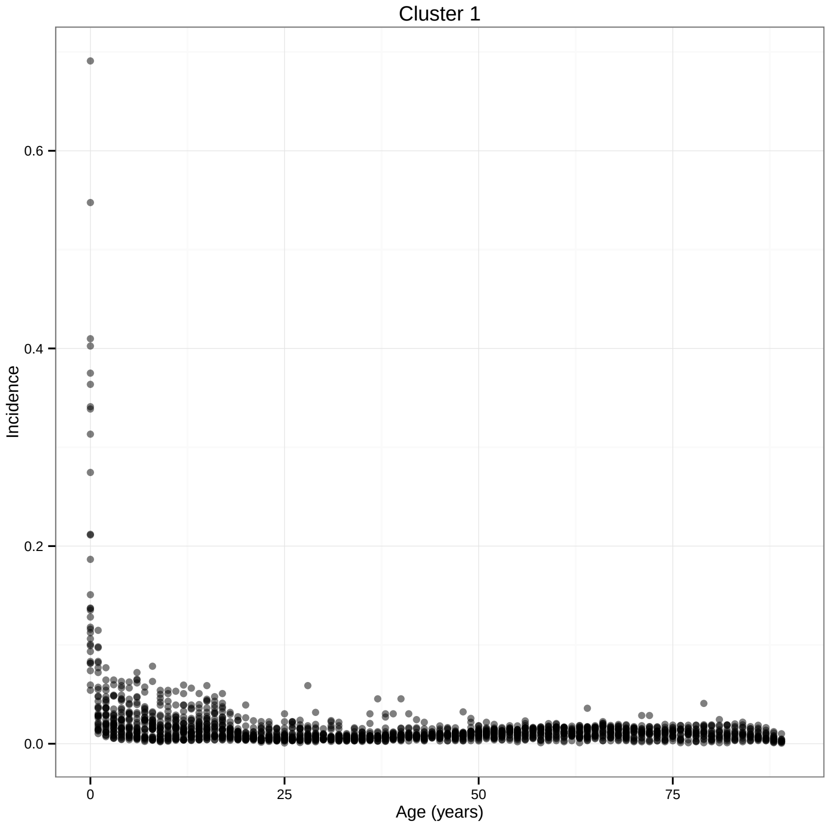

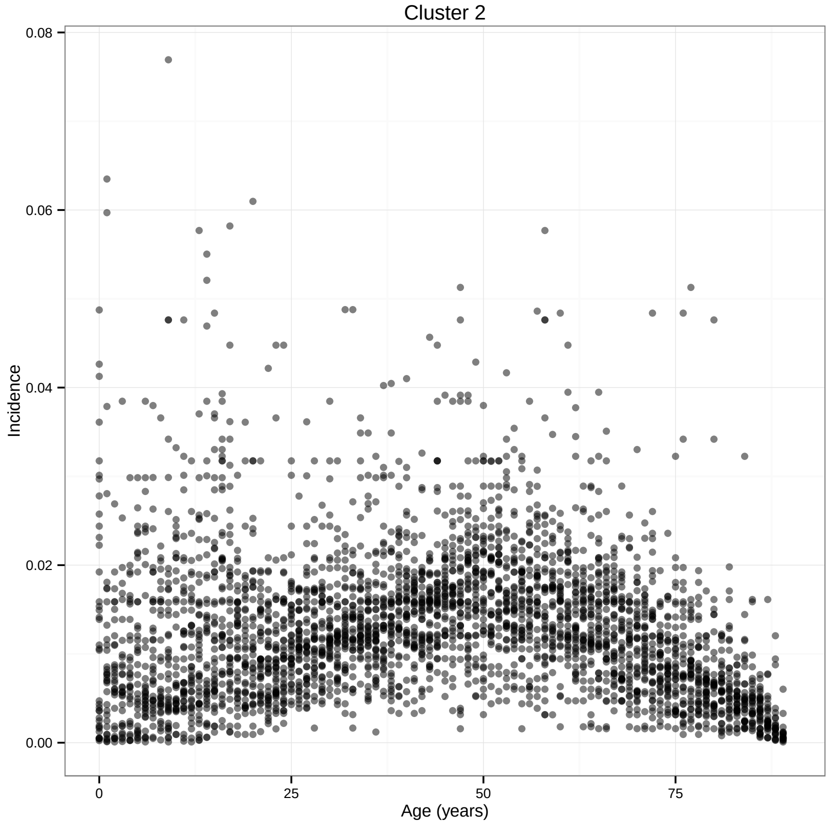
Cluster 2
Incidence
0.08
0.06
0.04
0.02
0.00
0
25
50
75
Age (years)

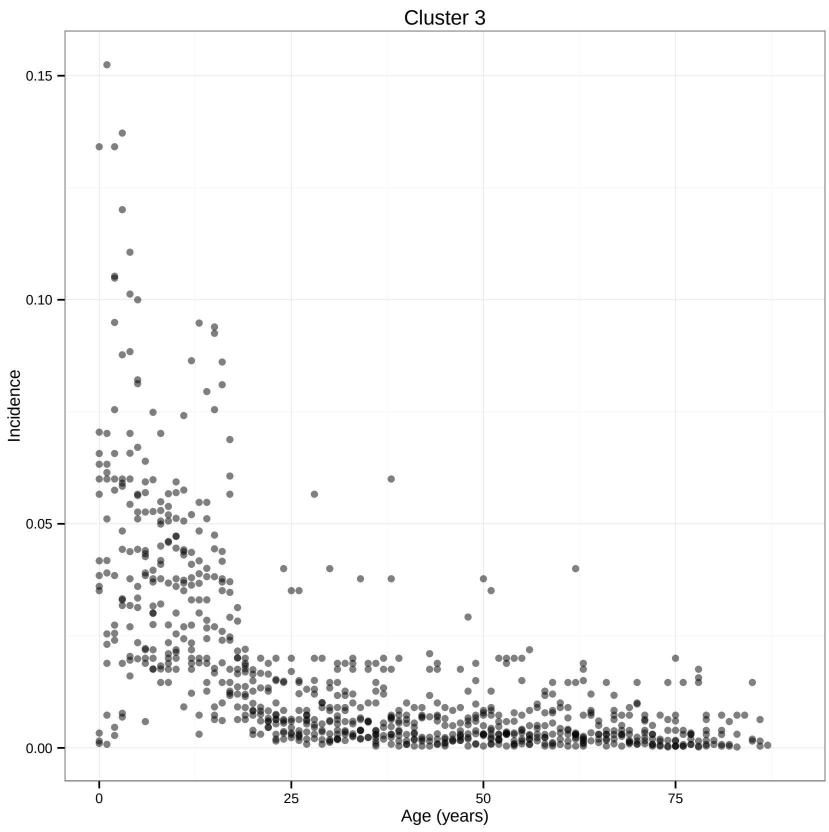
Cluster 3
Incidence
0.15
0.10
0.05
0.00
0
25
50
75
Age (years)

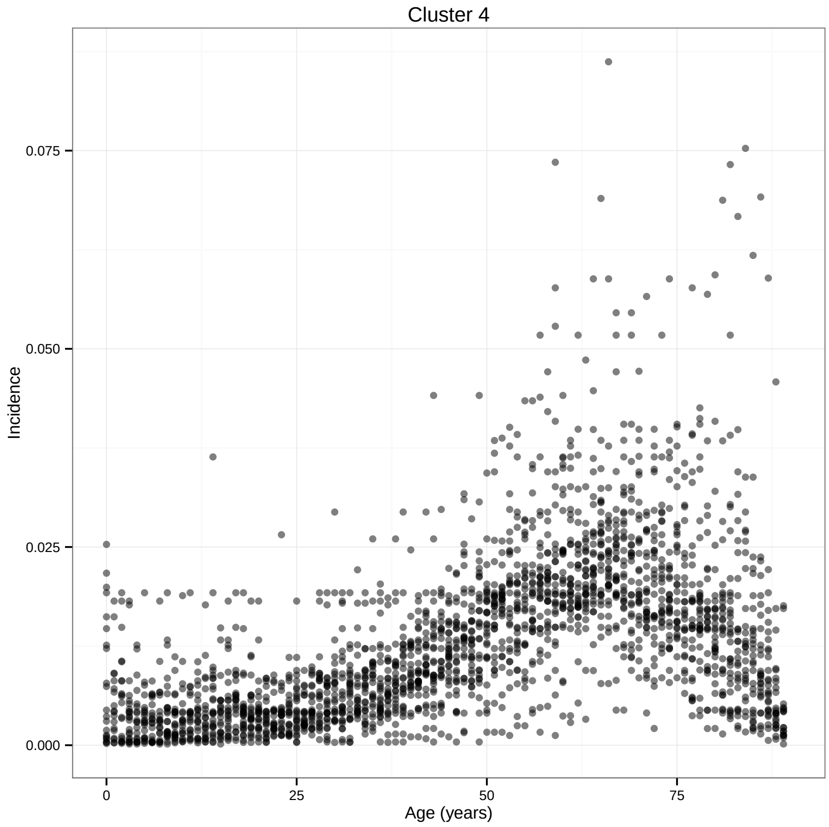
Cluster 4
Incidence
0.000
0.025
0.050
0.075
0
25
50
75
Age (years)

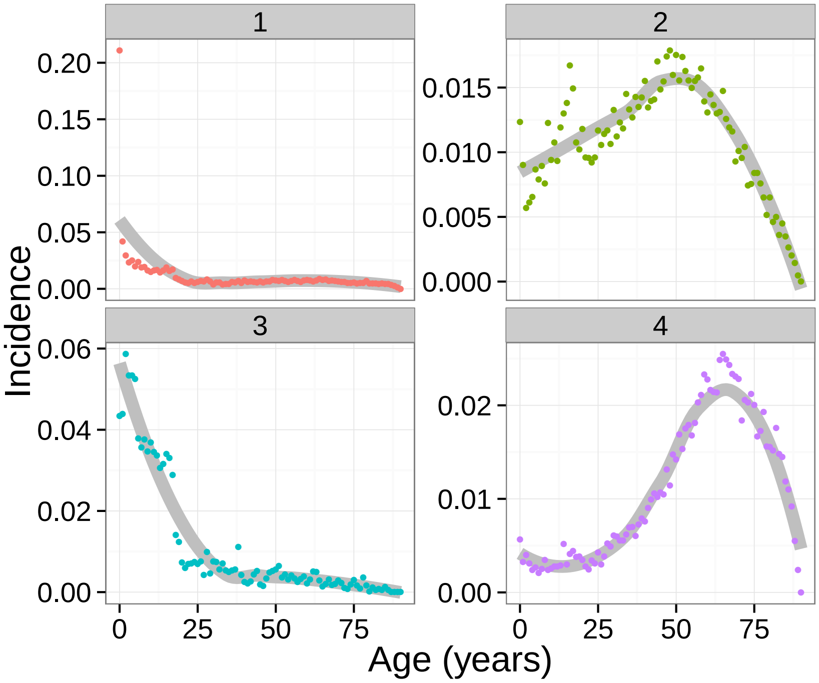

1
2
3
4
0.20
0.15
0.10
0.05
0.00
0.015
0.010
0.005
0.000
0.06
0.04
0.02
0.00
0.02
0.01
0.00
Incidence
0
25
50
75
Age (years)

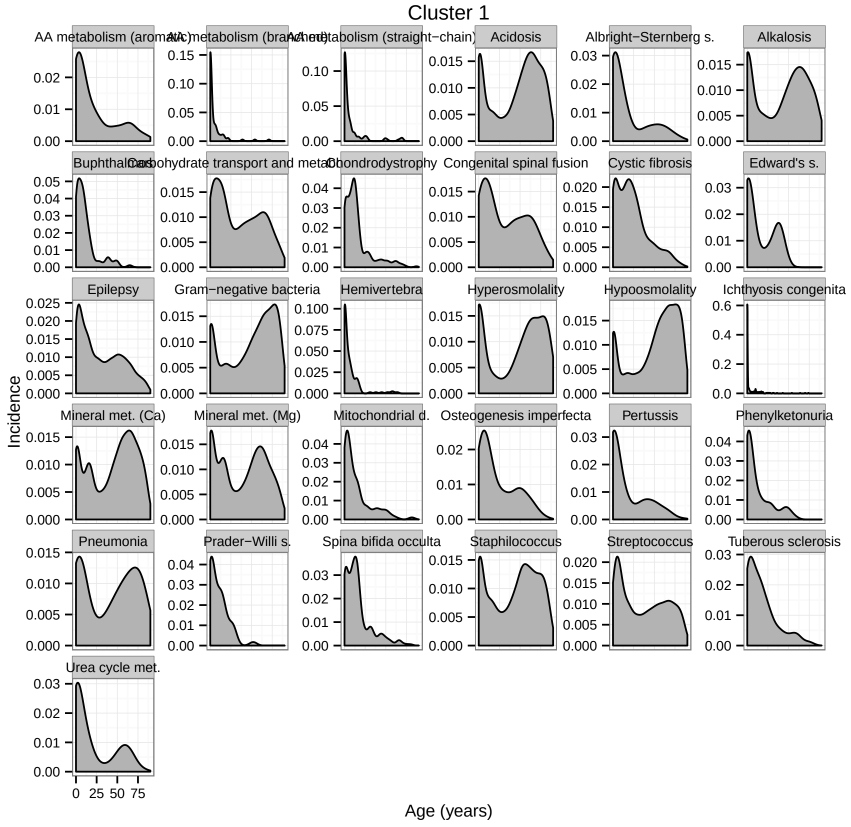
Cluster 1
AA metabolism (aromatic)
AA metabolism (branched)
AA metabolism (straight−chain)
Acidosis
Albright−Sternberg s.
Alkalosis
Buphthalmos
Carbohydrate transport and metab.
Chondrodystrophy
Congenital spinal fusion
Cystic fibrosis
Edward's s.
Epilepsy
Gram−negative bacteria
Hemivertebra
Hyperosmolality
Hypoosmolality
Ichthyosis congenita
Mineral met. (Ca)
Mineral met. (Mg)
Mitochondrial d.
Osteogenesis imperfecta
Pertussis
Phenylketonuria
Pneumonia
Prader−Willi s.
Spina bifida occulta
Staphilococcus
Streptococcus
Tuberous sclerosis
Urea cycle met.
Incidence
Age (years)

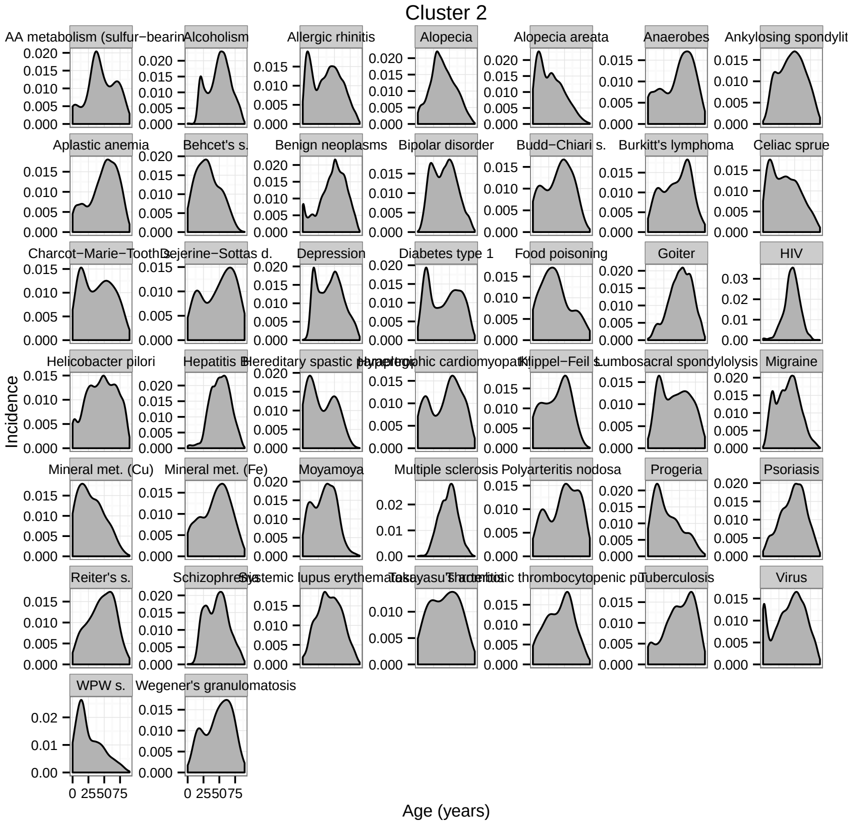
Cluster 2
Incidence
Age (years)
AA metabolism (sulfur−bearin
Alcoholism
Allergic rhinitis
Alopecia
Alopecia areata
Anaerobes
Ankylosing spondylit
Aplastic anemia
Behcet's s.
Benign neoplasms
Bipolar disorder
Budd−Chiari s.
Burkitt's lymphoma
Celiac sprue
Charcot−Marie−Tooth
Dejerine−Sottas d.
Depression
Diabetes type 1
Food poisoning
Goiter
HIV
Helicobacter pilori
Hepatitis B
Hereditary spastic
Hypertrophic cardiomyopath
Klippel−Feil s.
Lumbosacral spondylolysis
Migraine
Mineral met. (Cu)
Mineral met. (Fe)
Moyamoya
Multiple sclerosis
Polyarteritis nodosa
Progeria
Psoriasis
Reiter's s.
Schizophrenia
Systemic lupus erythematosus
Takayasu's arteritis
Thrombotic thrombocytopenic pu
Tuberculosis
Virus
WPW s.
Wegener's granulomatosis

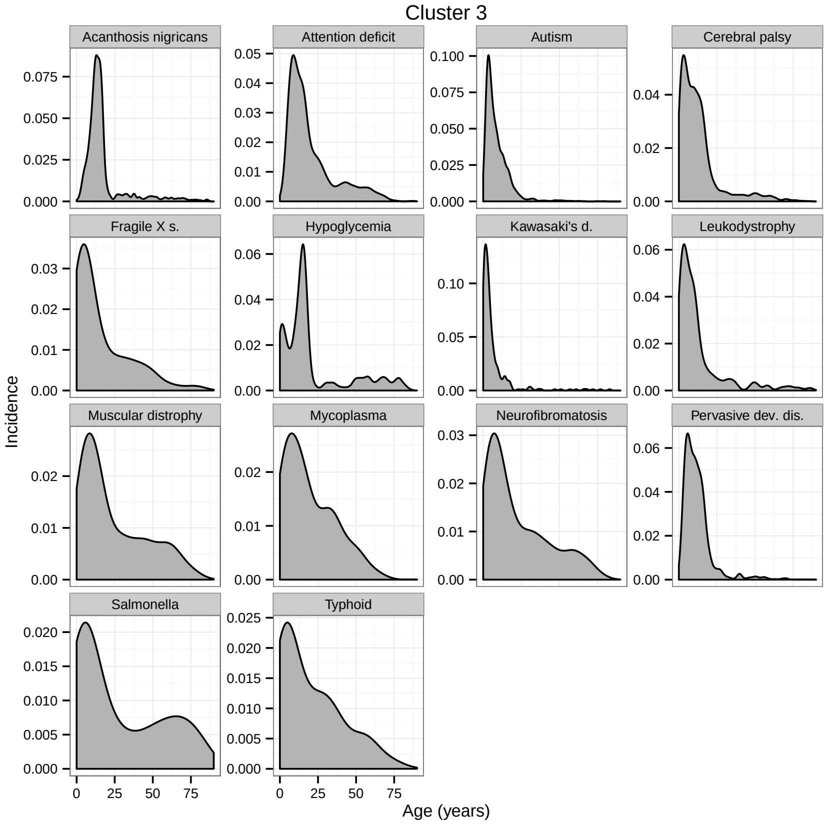
Cluster 3
Acanthosis nigricans
Attention deficit
Autism
Cerebral palsy
Fragile X s.
Hypoglycemia
Kawasaki's d.
Leukodystrophy
Muscular distrophy
Mycoplasma
Neurofibromatosis
Pervasive dev. dis.
Salmonella
Typhoid
Incidence
Age (years)
0
25
50
75

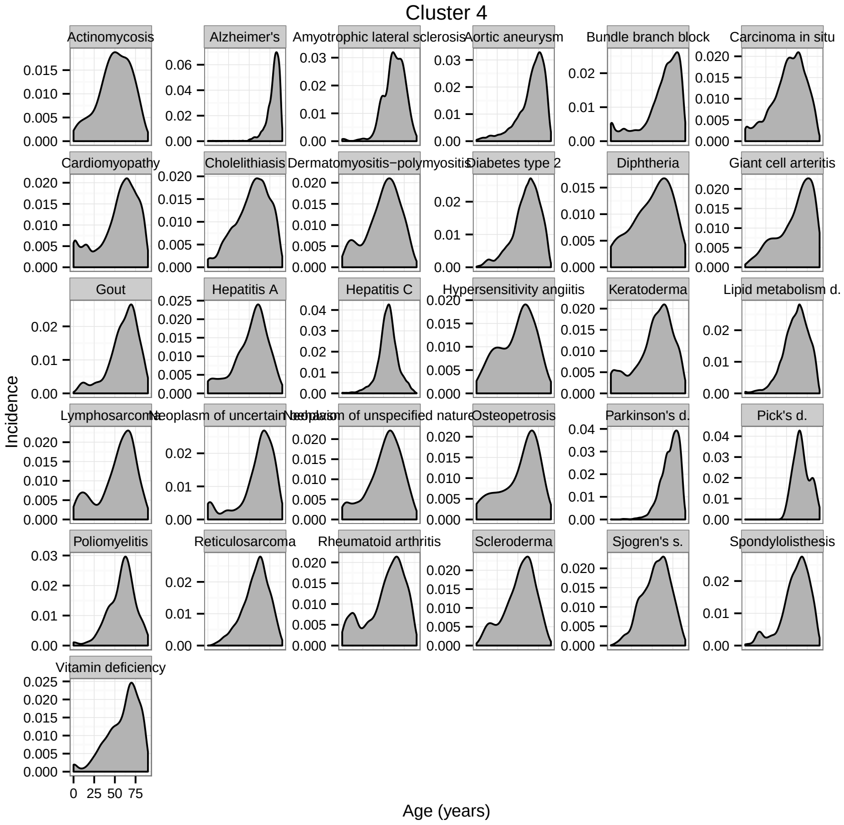
Cluster 4
Actinomycosis
Alzheimer's
Amyotrophic lateral sclerosis
Aortic aneurysm
Bundle branch block
Carcinoma in situ
Cardiomyopathy
Cholelithiasis
Dermatomyositis−polymyositis
Diabetes type 2
Diphtheria
Giant cell arteritis
Gout
Hepatitis A
Hepatitis C
Hypersensitivity angiitis
Keratoderma
Lipid metabolism d.
Lymphosarcoma
Neoplasm of uncertain behavior
Neoplasm of unspecified nature
Osteopetrosis
Parkinson's d.
Pick's d.
Poliomyelitis
Reticulosarcoma
Rheumatoid arthritis
Scleroderma
Sjogren's s.
Spondylolisthesis
Vitamin deficiency
Incidence
Age (years)

5.4 cluster size 5

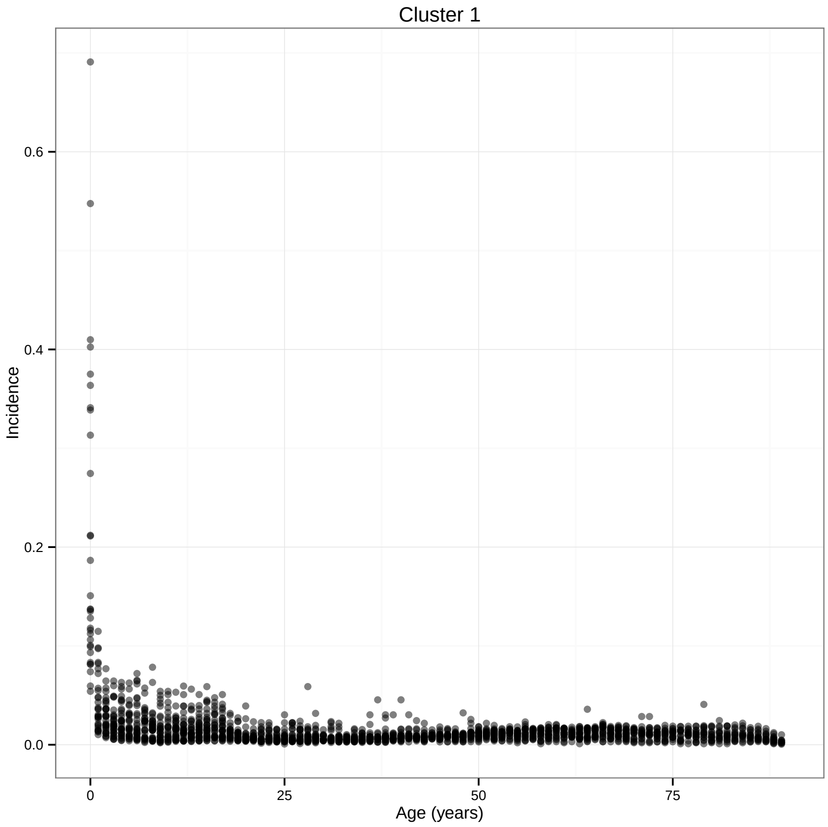

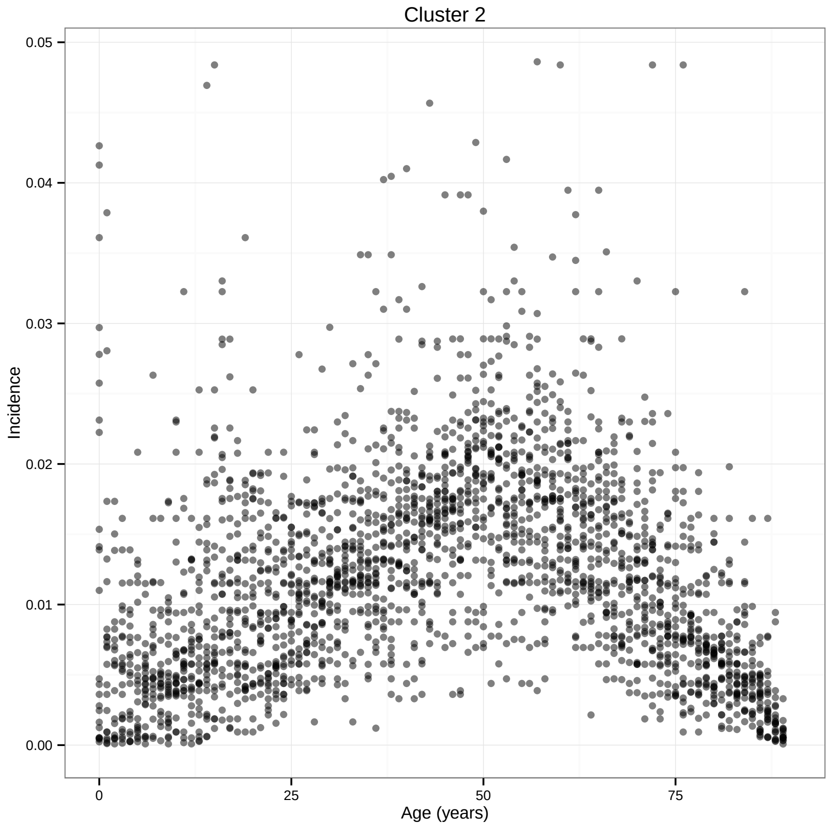
Cluster 2
Incidence
0.05
0.04
0.03
0.02
0.01
0.00
0
25
50
75
Age (years)

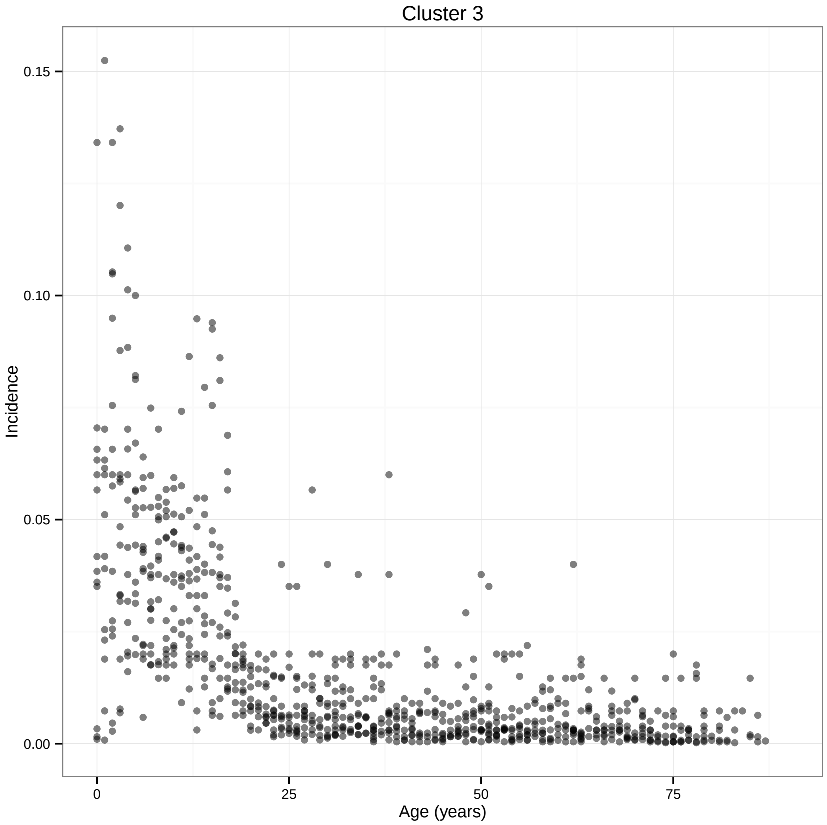
Cluster 3
Incidence
0.15
0.10
0.05
0.00
0
25
50
75
Age (years)

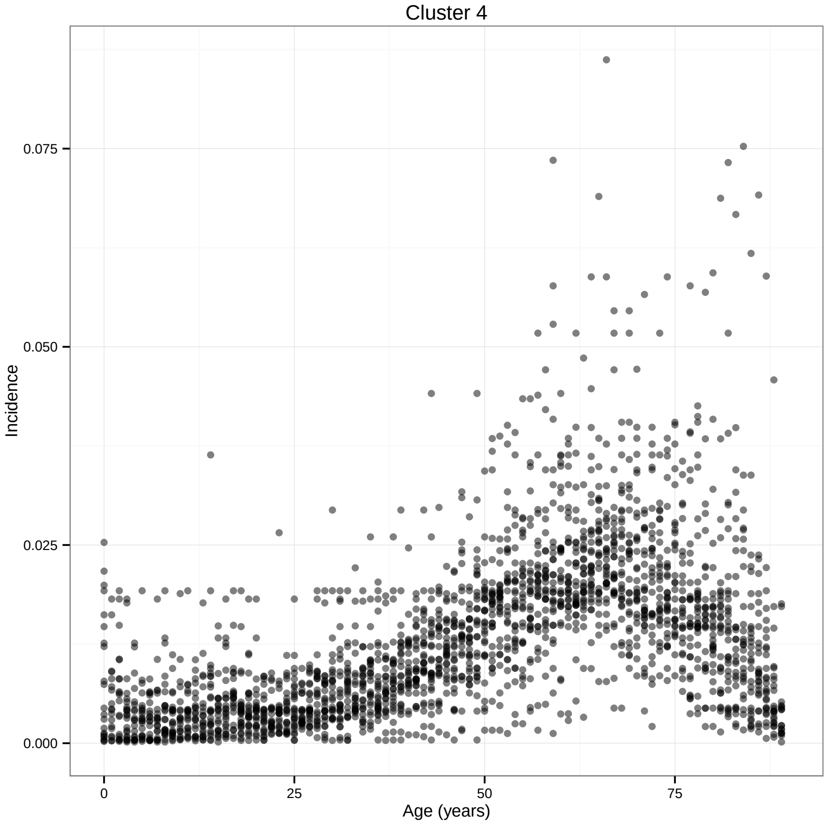
Cluster 4
Incidence
0.075
0.050
0.025
0.000
0
25
50
75
Age (years)

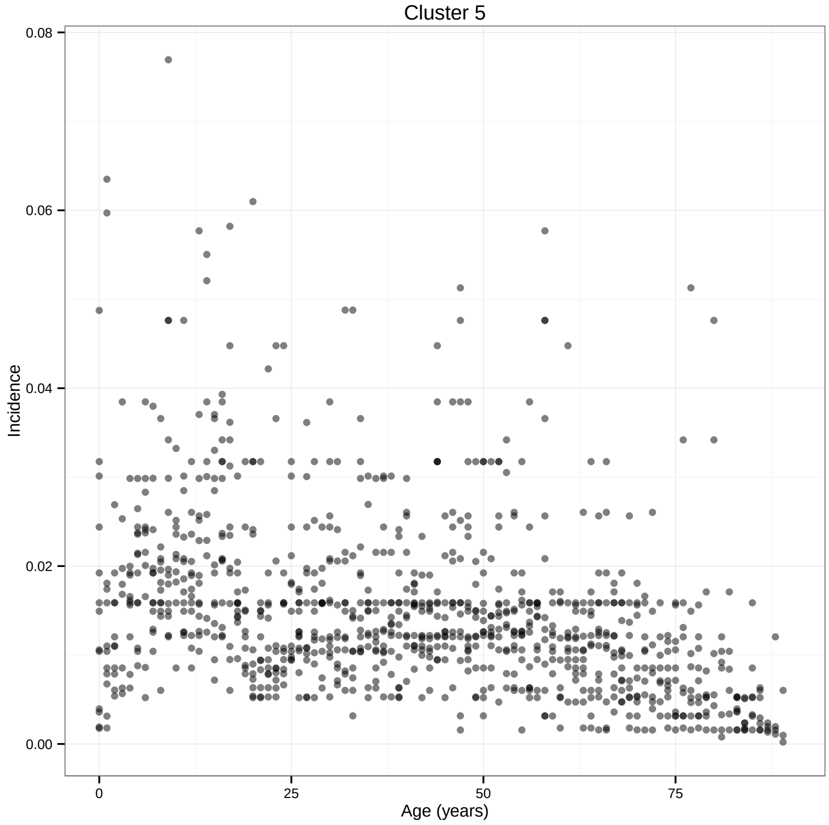
Cluster 5
0.08
0.06
0.04
0.02
0.00
Incidence
0
25
50
75
Age (years)

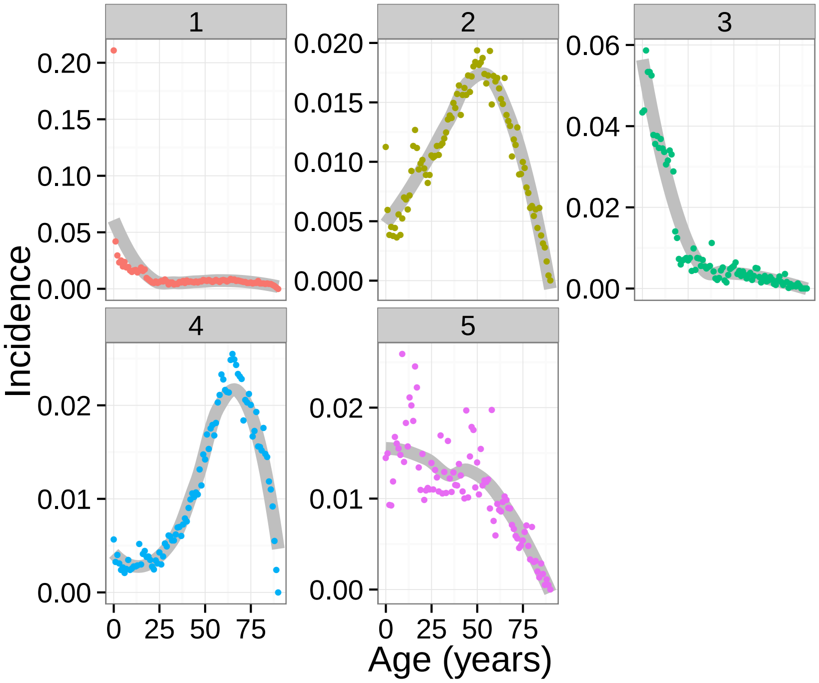
1
2
3
4
5
Incidence
Age (years)
0.20
0.15
0.10
0.05
0.00
0.020
0.015
0.010
0.005
0.000
0.06
0.04
0.02
0.00
0.02
0.01
0.00
0.02
0.01
0.00
0 25 50 75
0 25 50 75

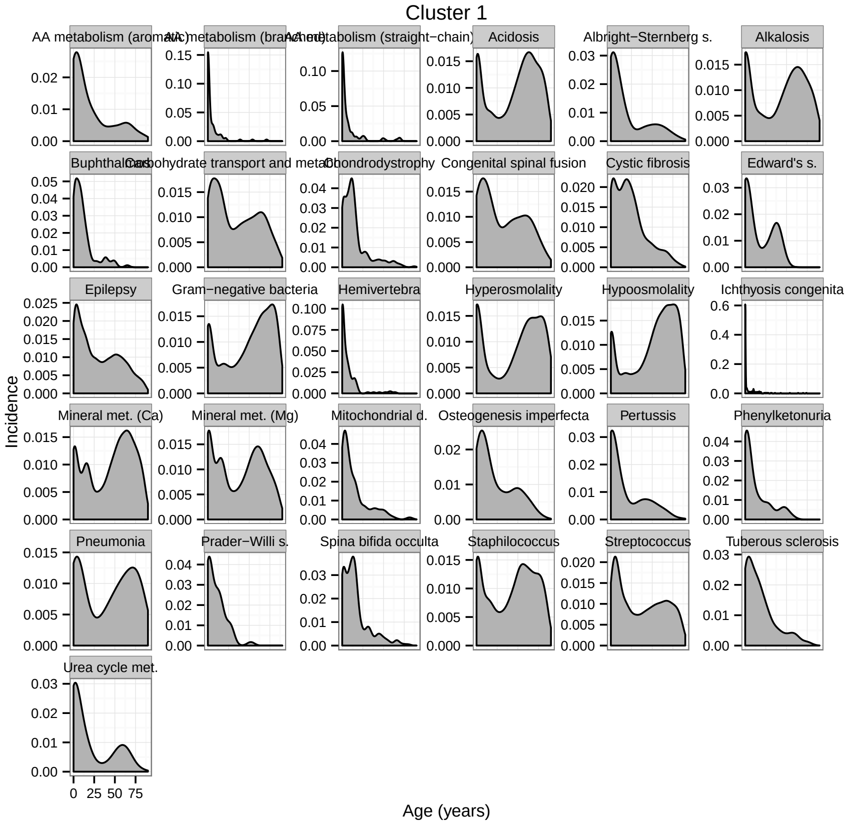
Cluster 1
AA metabolism (aromatic)
AA metabolism (branched)
AA metabolism (straight−chain)
Acidosis
Albright−Sternberg s.
Alkalosis
Buphthalmos
Carbohydrate transport and metab.
Chondrodystrophy
Congenital spinal fusion
Cystic fibrosis
Edward's s.
Epilepsy
Gram−negative bacteria
Hemivertebra
Hyperosmolality
Hypoosmolality
Ichthyosis congenita
Mineral met. (Ca)
Mineral met. (Mg)
Mitochondrial d.
Osteogenesis imperfecta
Pertussis
Phenylketonuria
Pneumonia
Prader−Willi s.
Spina bifida occulta
Staphilococcus
Streptococcus
Tuberous sclerosis
Urea cycle met.
Incidence
Age (years)
0 25 50 75

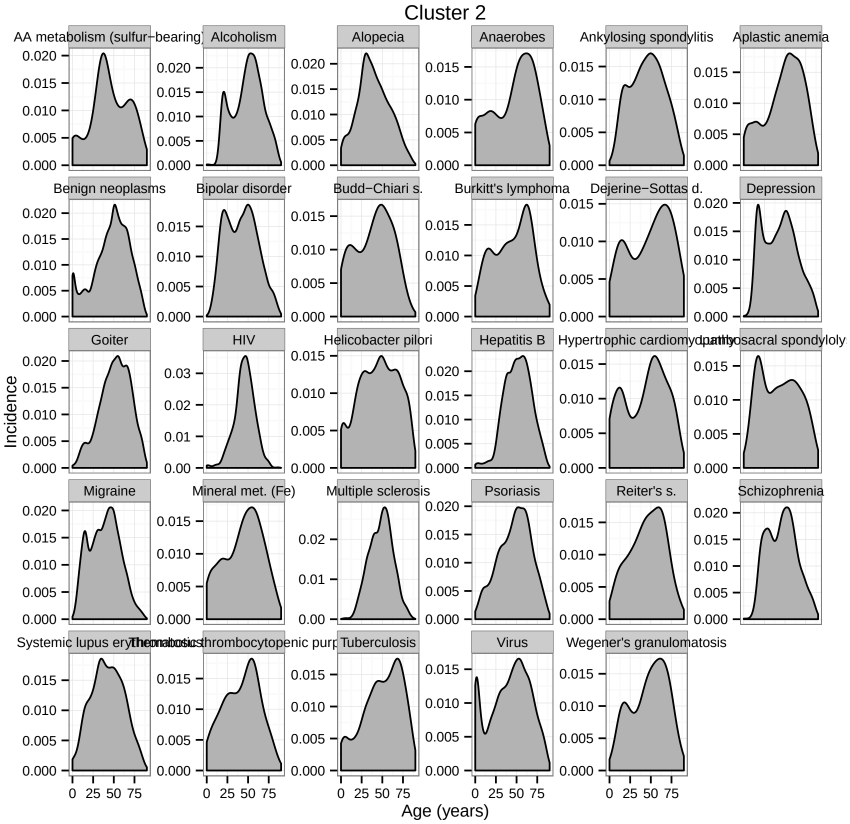

Cluster 2
AA metabolism (sulfur−bearing)
Alcoholism
Alopecia
Anaerobes
Ankylosing spondylitis
Aplastic anemia
Benign neoplasms
Bipolar disorder
Budd−Chiari s.
Burkitt's lymphoma
Dejerine−Sottas d.
Depression
Goiter
HIV
Helicobacter pilori
Hepatitis B
Hypertrophic cardiomyopathy
Lumbosacral spondyloly
Migraine
Mineral met. (Fe)
Multiple sclerosis
Psoriasis
Reiter's s.
Schizophrenia
Systemic lupus erythematosus
Thrombotic thrombocytopenic purp
Tuberculosis
Virus
Wegener's granulomatosis
Incidence
Age (years)

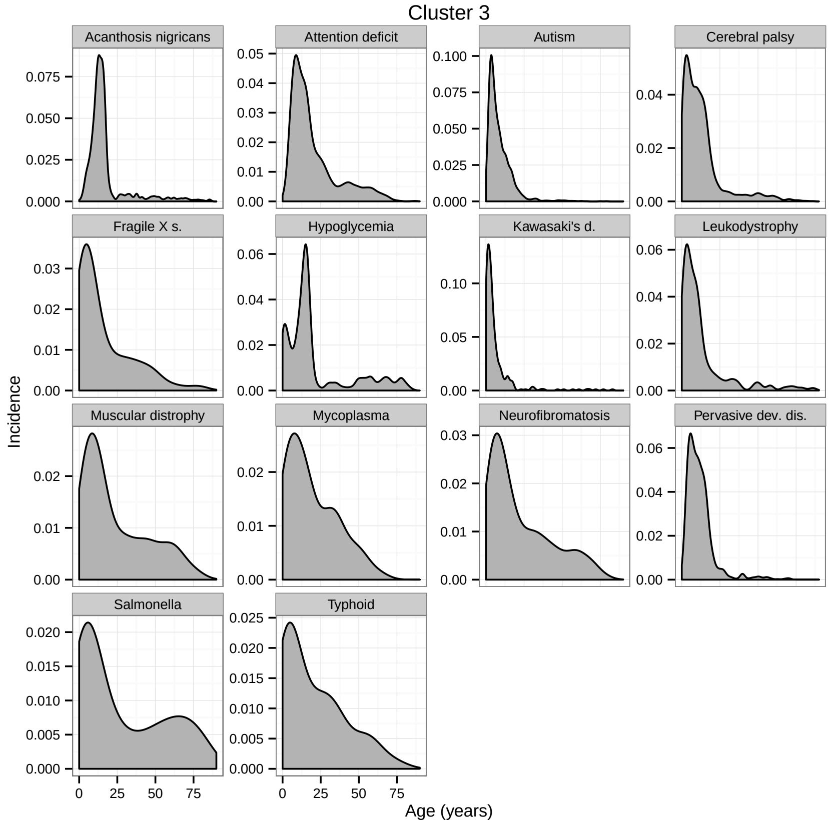
Cluster 3
Acanthosis nigricans
Attention deficit
Autism
Cerebral palsy
Fragile X s.
Hypoglycemia
Kawasaki's d.
Leukodystrophy
Muscular distrophy
Mycoplasma
Neurofibromatosis
Pervasive dev. dis.
Salmonella
Typhoid
Incidence
Age (years)

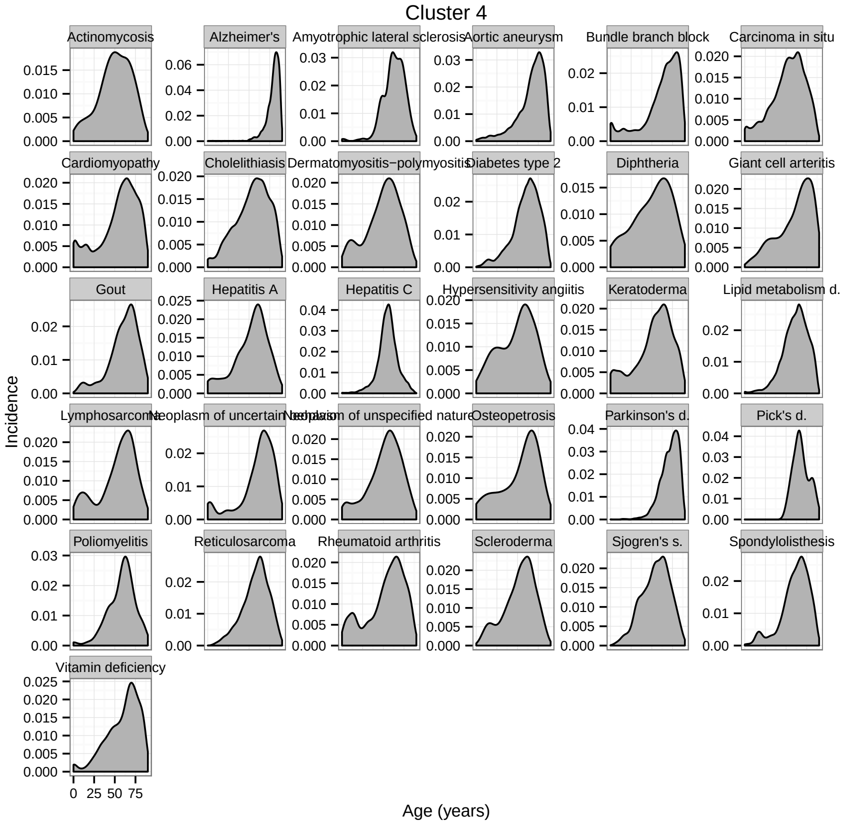

Cluster 4
Actinomycosis
Alzheimer's
Amyotrophic lateral sclerosis
Aortic aneurysm
Bundle branch block
Carcinoma in situ
Cardiomyopathy
Cholelithiasis
Dermatomyositis−polymyositis
Diabetes type 2
Diphtheria
Giant cell arteritis
Gout
Hepatitis A
Hepatitis C
Hypersensitivity angiitis
Keratoderma
Lipid metabolism d.
Lymphosarcoma
Neoplasm of uncertain behav.
Neoplasm of unspecified nature
Osteopetrosis
Parkinson's d.
Pick's d.
Poliomyelitis
Reticulosarcoma
Rheumatoid arthritis
Scleroderma
Sjogren's s.
Spondylolisthesis
Vitamin deficiency
Incidence
Age (years)
0 25 50 75

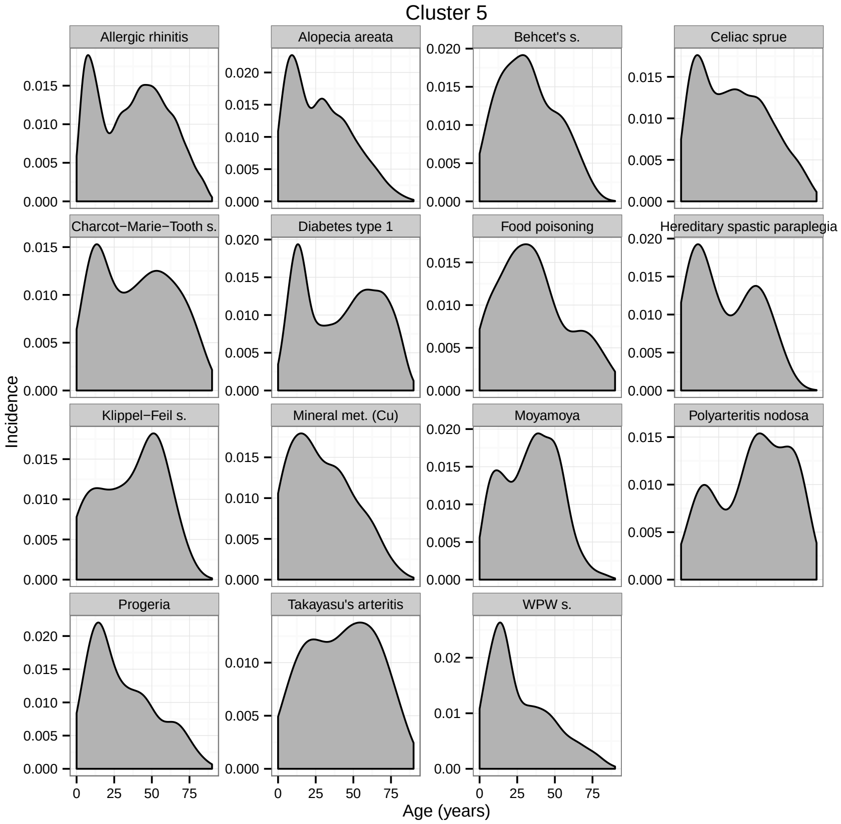
Cluster 5
Allergic rhinitis
Alopecia areata
Behcet's s.
Celiac sprue
Charcot−Marie−Tooth s.
Diabetes type 1
Food poisoning
Hereditary spastic paraplegia
Klippel−Feil s.
Mineral met. (Cu)
Moyamoya
Polyarteritis nodosa
Progeria
Takayasu's arteritis
WPW s.
Incidence
Age (years)
0 25 50 75

5.5 cluster size 6

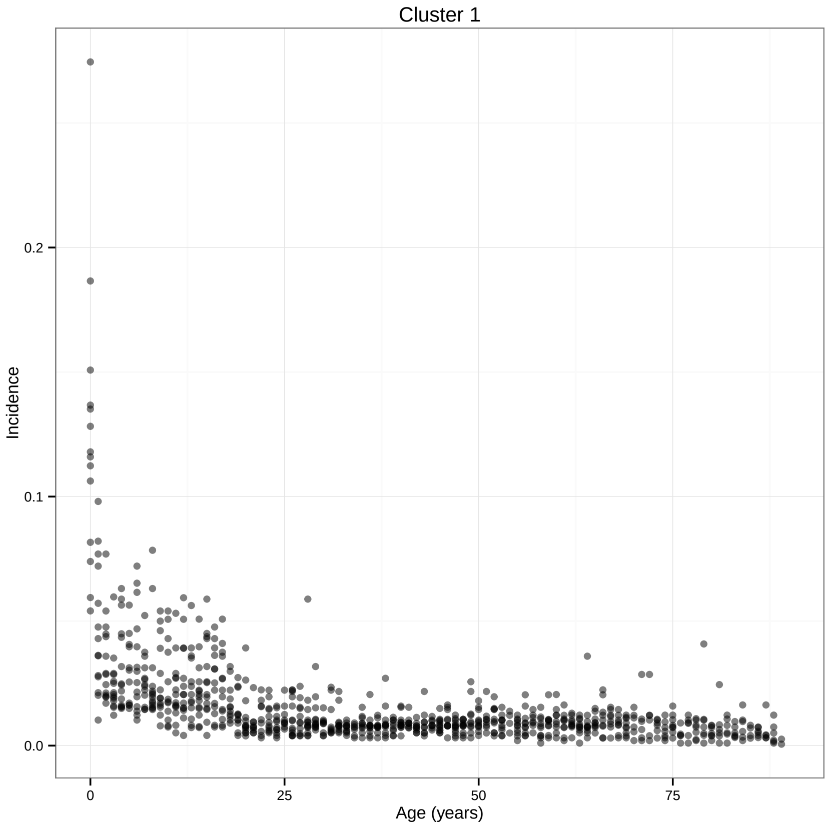

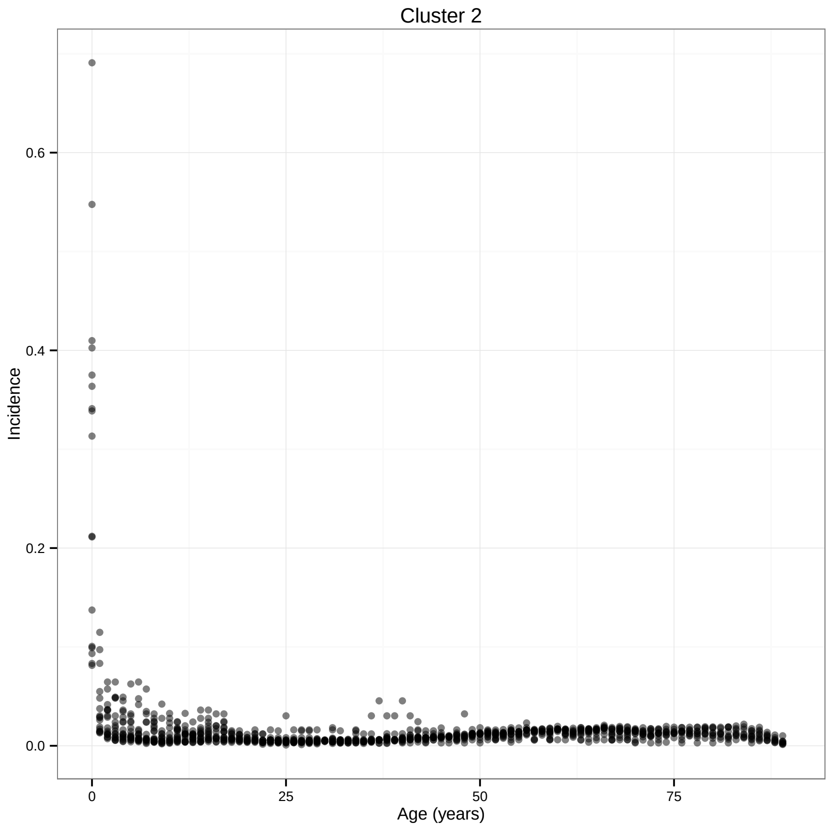

Cluster 2
Incidence
0.0
0.2
0.4
0.6
0
25
50
75
Age (years)

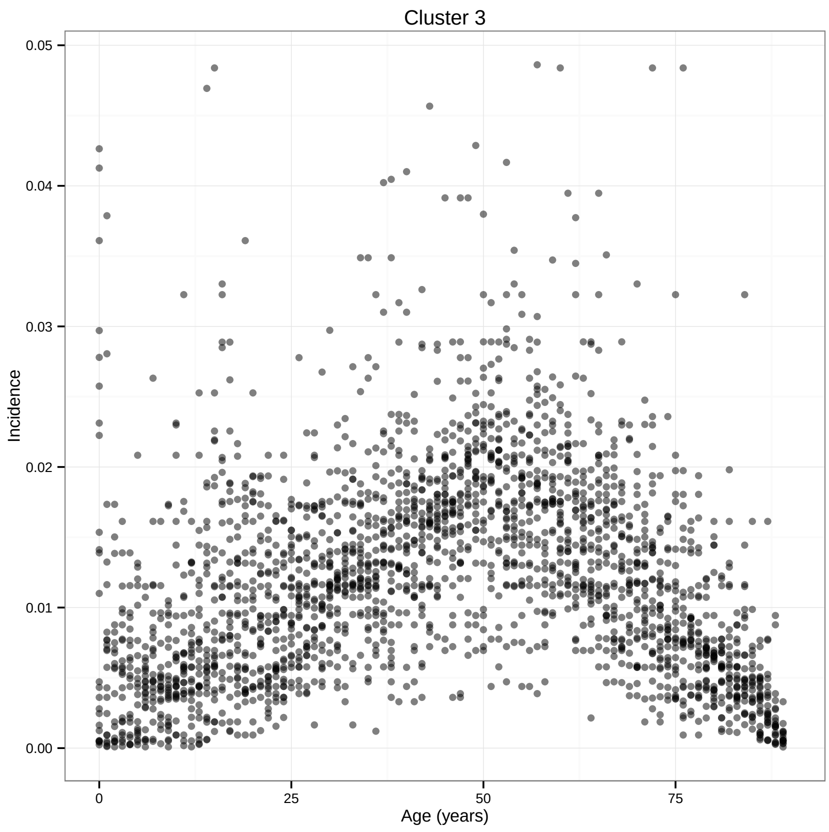
Cluster 3
Incidence
0.05
0.04
0.03
0.02
0.01
0.00
0
25
50
75
Age (years)

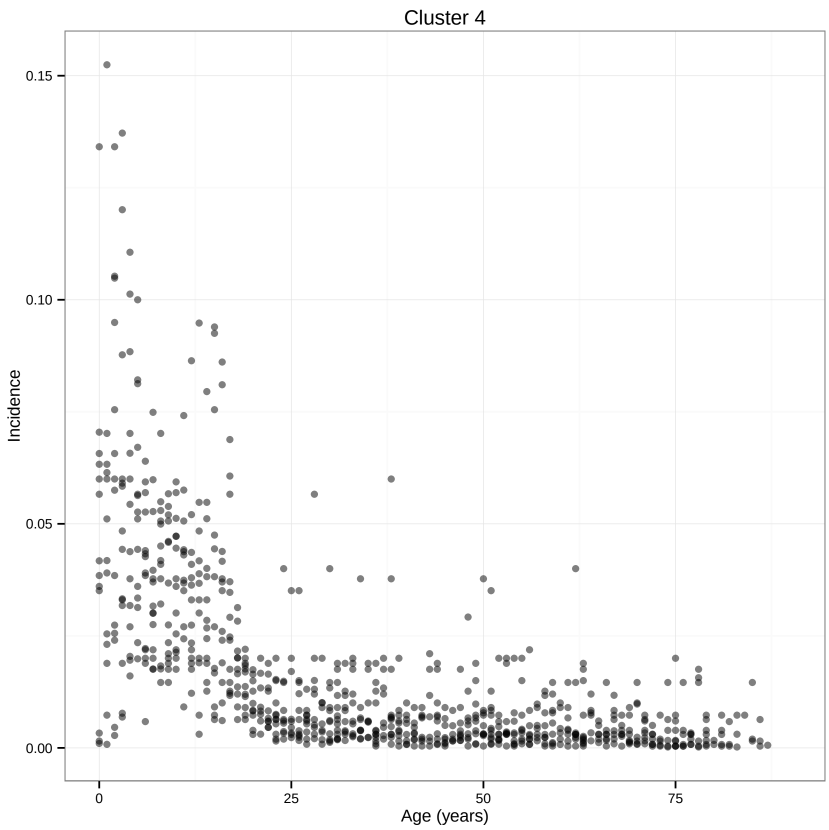
Cluster 4
Incidence
0.15
0.10
0.05
0.00
0
25
50
75
Age (years)

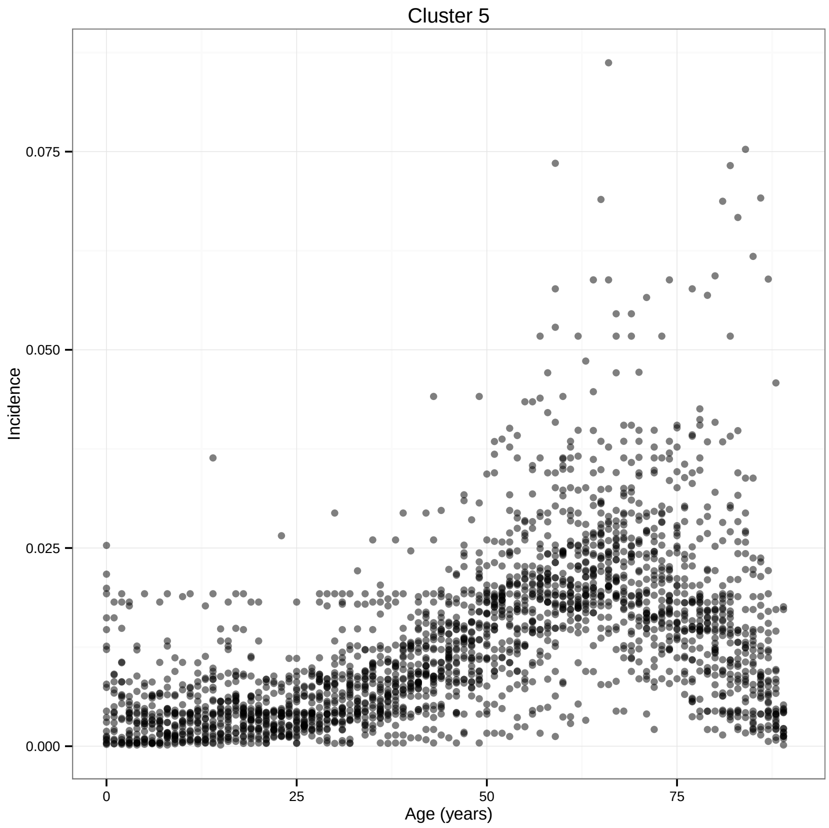

Cluster 5
Incidence
0.075
0.050
0.025
0.000
0
25
50
75
Age (years)

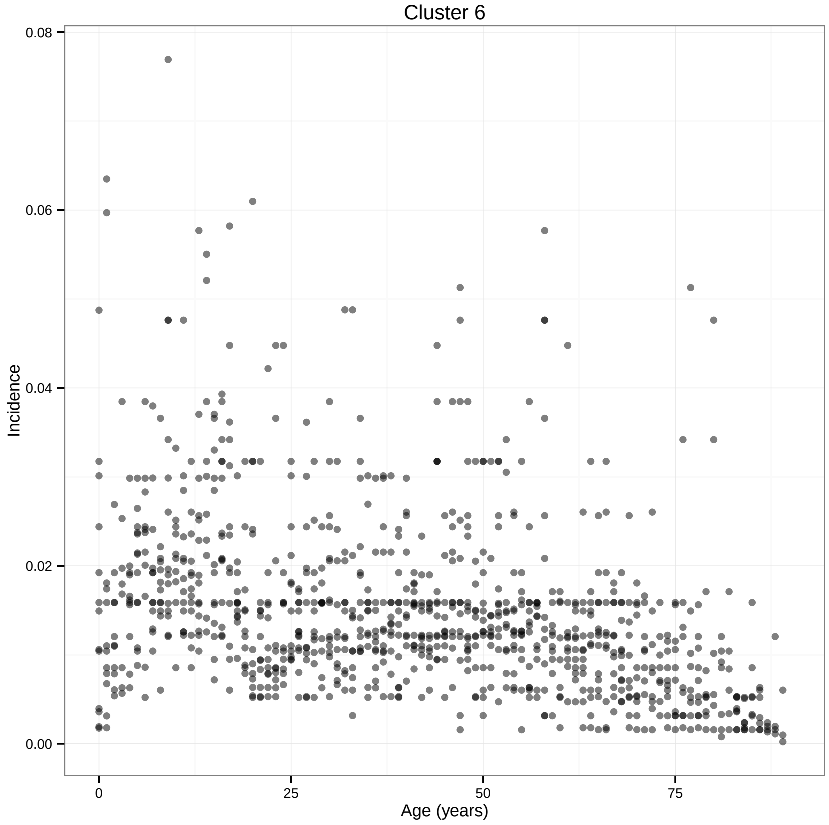

Cluster 6
0.08
0.06
0.04
0.02
0.00
Incidence
0
25
50
75
Age (years)

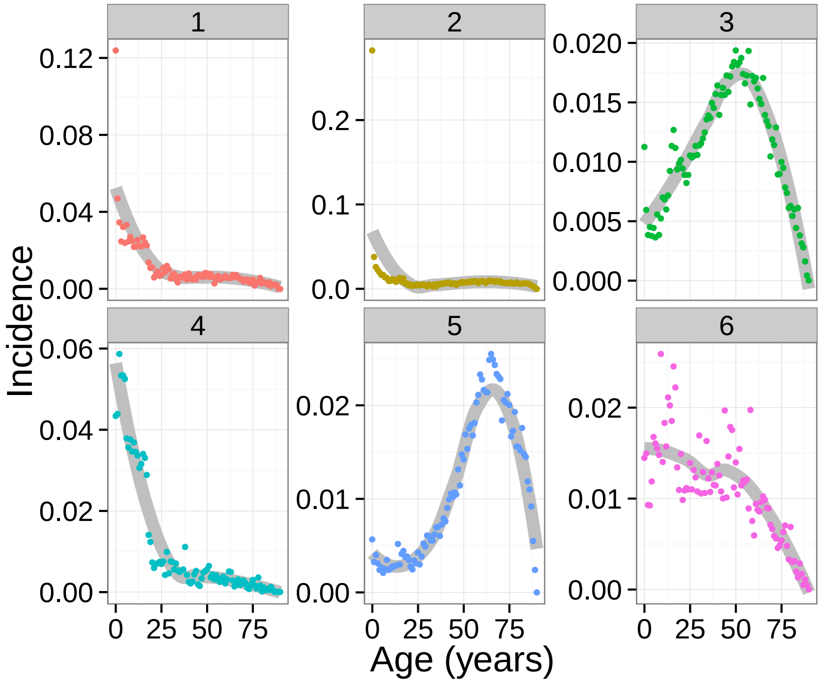
1
2
3
4
5
6
Incidence
Age (years)
0.12
0.08
0.04
0.00
0.2
0.1
0.0
0.020
0.015
0.010
0.005
0.000
0.06
0.04
0.02
0.00
0.02
0.01
0.00
0.02
0.01
0.00
0
25
50
75

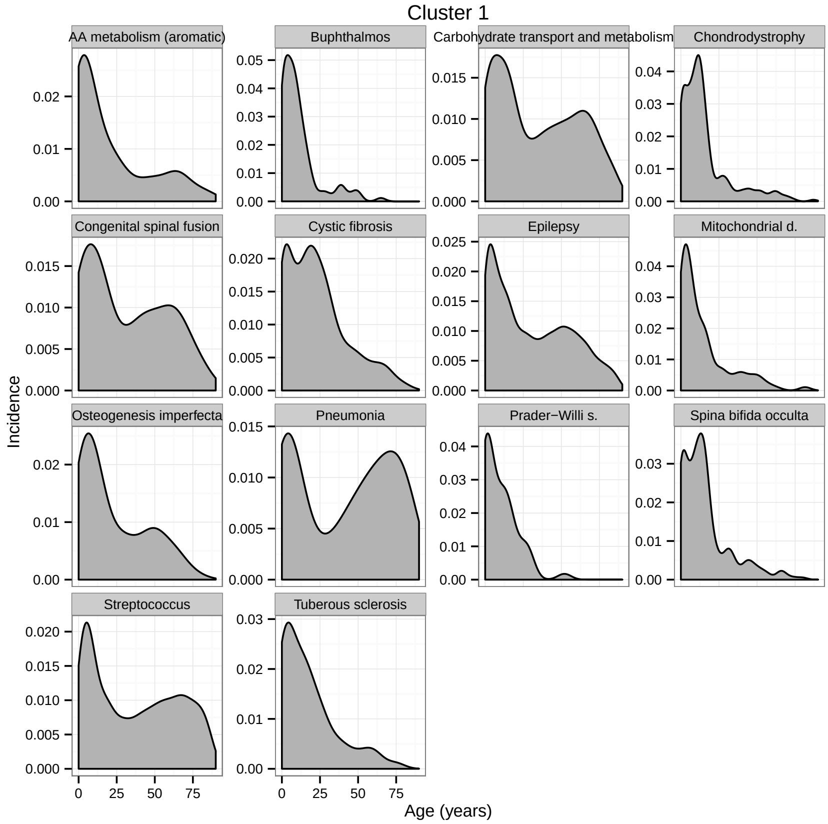
Cluster 1
AA metabolism (aromatic)
Buphthalmos
Carbohydrate transport and metabolism
Chondrodystrophy
Congenital spinal fusion
Cystic fibrosis
Epilepsy
Mitochondrial d.
Osteogenesis imperfecta
Pneumonia
Prader−Willi s.
Spina bifida occulta
Streptococcus
Tuberous sclerosis
Incidence
Age (years)
0
25
50
75

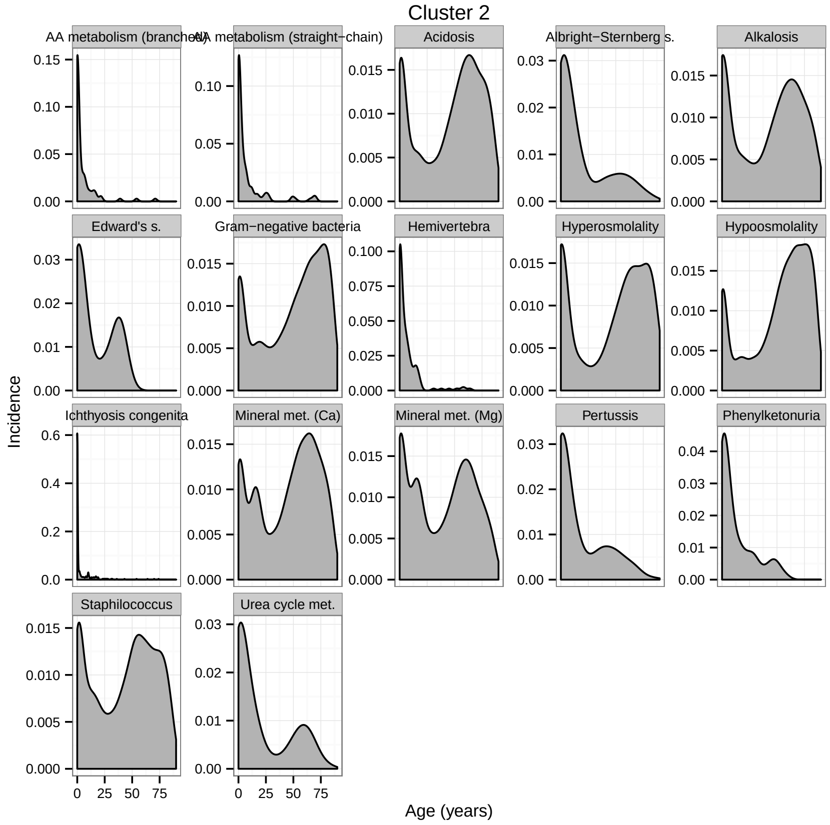
Cluster 2
AA metabolism (branched)
AA metabolism (straight−chain)
Acidosis
Albright−Sternberg s.
Alkalosis
Edward's s.
Gram−negative bacteria
Hemivertebra
Hyperosmolality
Hypoosmolality
Ichthyosis congenita
Mineral met. (Ca)
Mineral met. (Mg)
Pertussis
Phenylketonuria
Staphilococcus
Urea cycle met.
Incidence
Age (years)

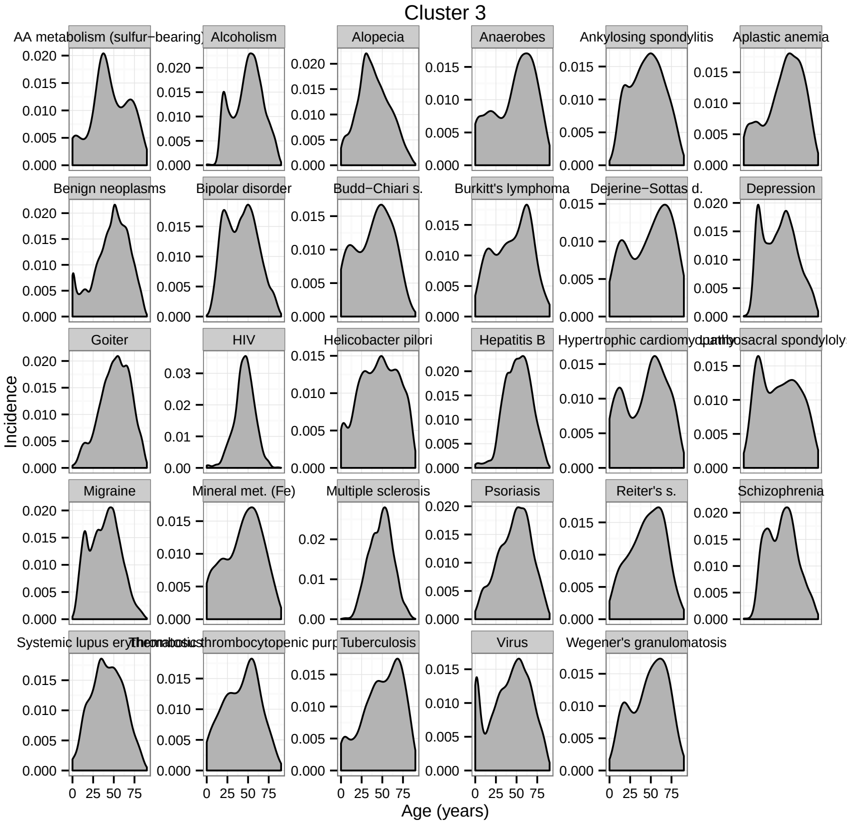
Cluster 3
AA metabolism (sulfur−bearing)
Alcoholism
Alopecia
Anaerobes
Ankylosing spondylitis
Aplastic anemia
Benign neoplasms
Bipolar disorder
Budd−Chiari s.
Burkitt's lymphoma
Dejerine−Sottas d.
Depression
Goiter
HIV
Helicobacter pilori
Hepatitis B
Psoriasis
Reiter's s.
Schizophrenia
Migraine
Mineral met. (Fe)
Multiple sclerosis
Tuberculosis
Virus
Wegener's granulomatosis
Incidence
Age (years)

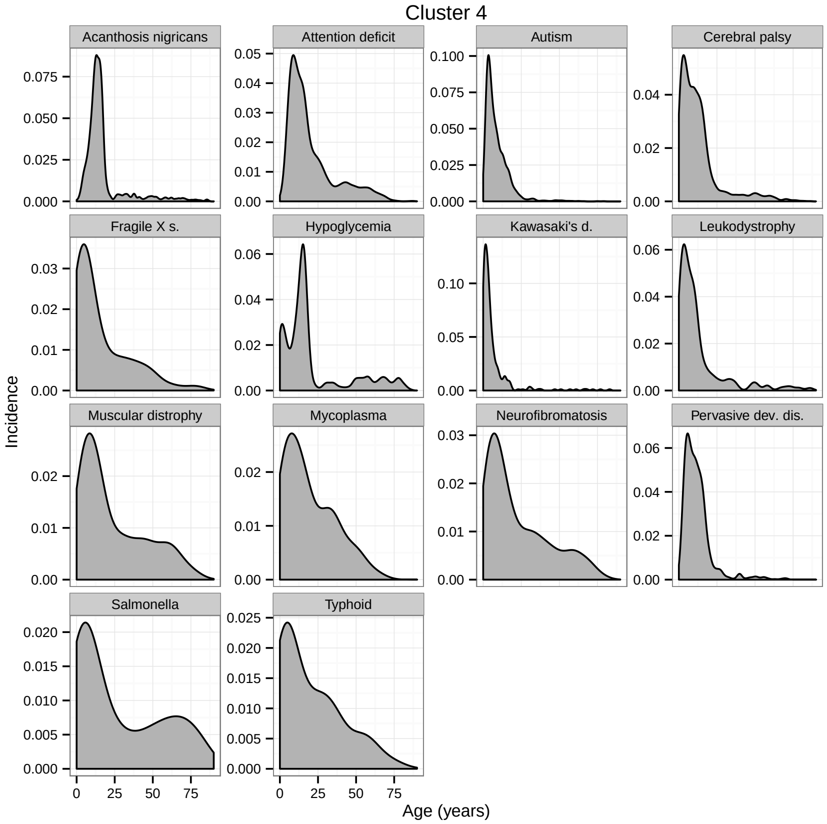
Cluster 4
Acanthosis nigricans
Attention deficit
Autism
Cerebral palsy
Fragile X s.
Hypoglycemia
Kawasaki's d.
Leukodystrophy
Muscular distrophy
Mycoplasma
Neurofibromatosis
Pervasive dev. dis.
Salmonella
Typhoid
Incidence
Age (years)

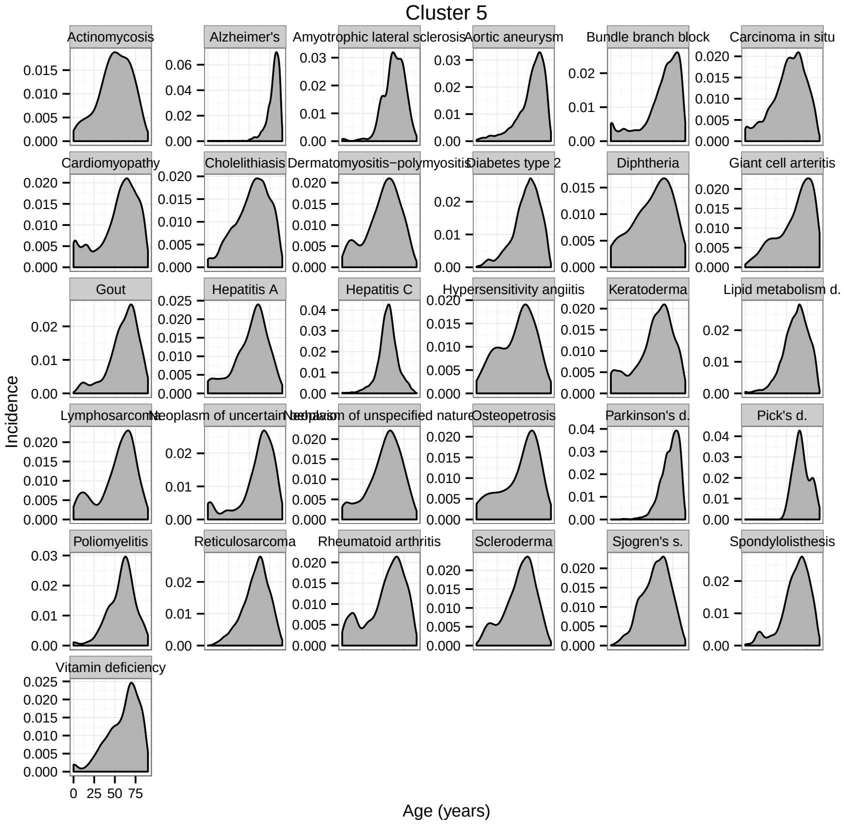
Cluster 5
Actinomycosis
Alzheimer's
Amyotrophic lateral sclerosis
Aortic aneurysm
Bundle branch block
Carcinoma in situ
Cardiomyopathy
Cholelithiasis
Dermatomyositis−polymyositis
Diabetes type 2
Diphtheria
Giant cell arteritis
Gout
Hepatitis A
Hepatitis C
Hypersensitivity angiitis
Keratoderma
Lipid metabolism d.
Lymphosarcoma
Neoplasm of uncertain behavior
Neoplasm of unspecified nature
Osteopetrosis
Parkinson's d.
Pick's d.
Poliomyelitis
Reticulosarcoma
Rheumatoid arthritis
Scleroderma
Sjogren's s.
Spondylolisthesis
Vitamin deficiency
Incidence
Age (years)

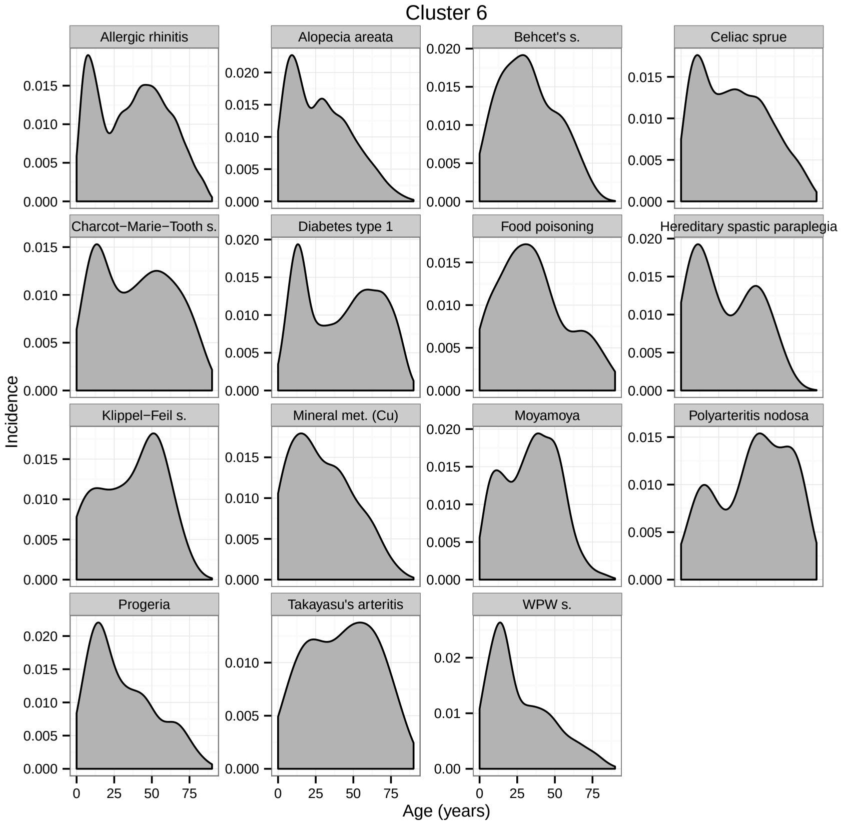
Cluster 6
Allergic rhinitis
Alopecia areata
Behcet's s.
Celiac sprue
Charcot−Marie−Tooth s.
Diabetes type 1
Food poisoning
Hereditary spastic paraplegia
Klippel−Feil s.
Mineral met. (Cu)
Moyamoya
Polyarteritis nodosa
Progeria
Takayasu's arteritis
WPW s.
Incidence
Age (years)

5.6 cluster size 7

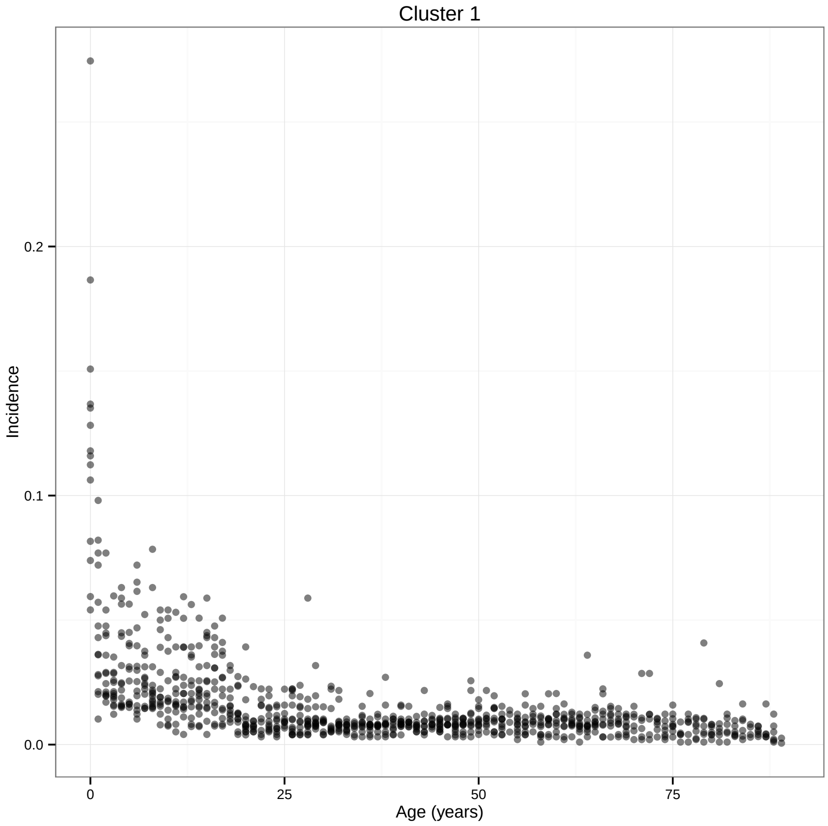

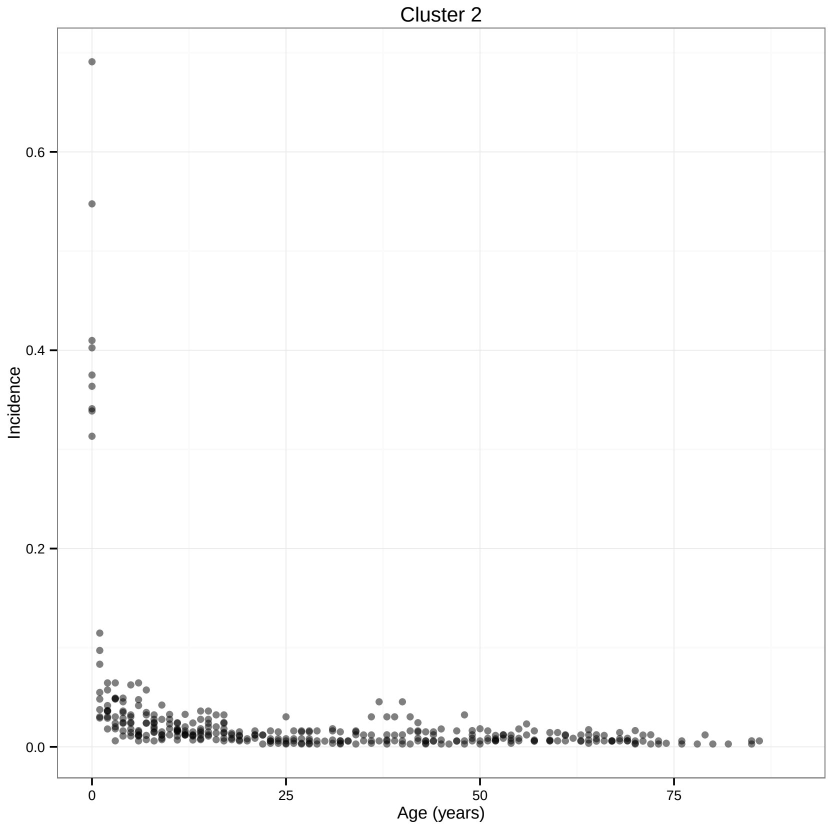
Cluster 2
Incidence
0.6
0.4
0.2
0.0
0
25
50
75
Age (years)

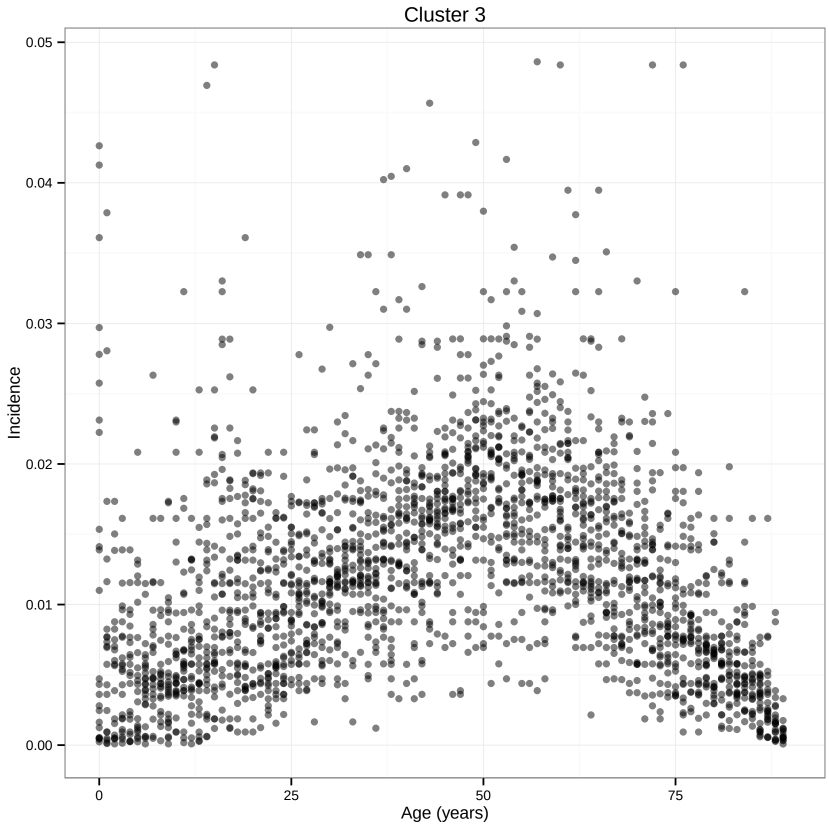
Cluster 3
Incidence
0.05
0.04
0.03
0.02
0.01
0.00
0
25
50
75
Age (years)

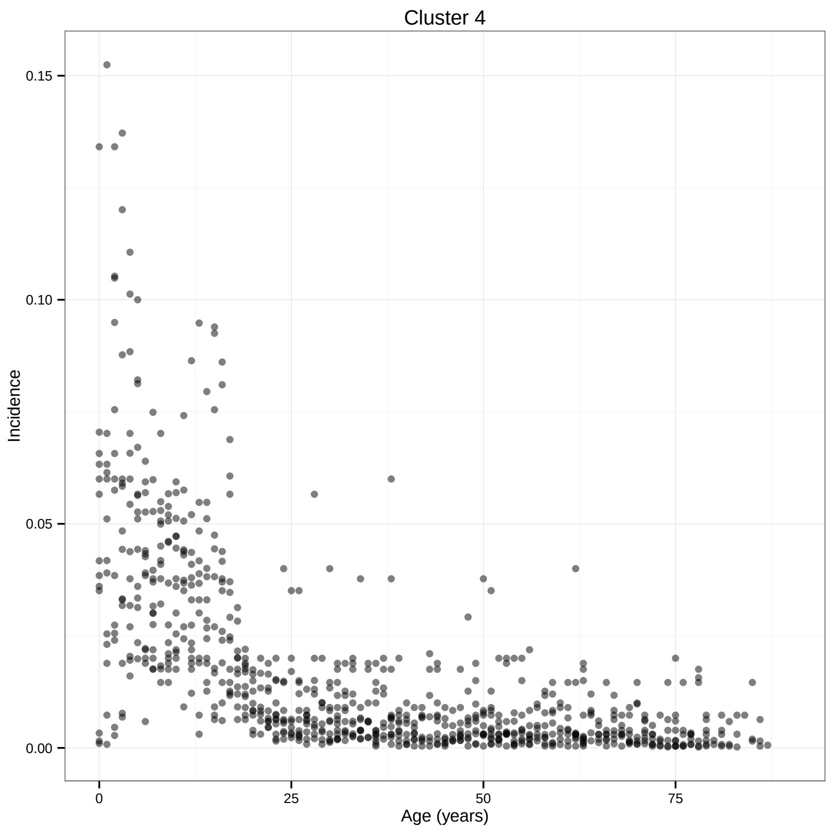
Cluster 4
Incidence
0.15
0.10
0.05
0.00
0
25
50
75
Age (years)

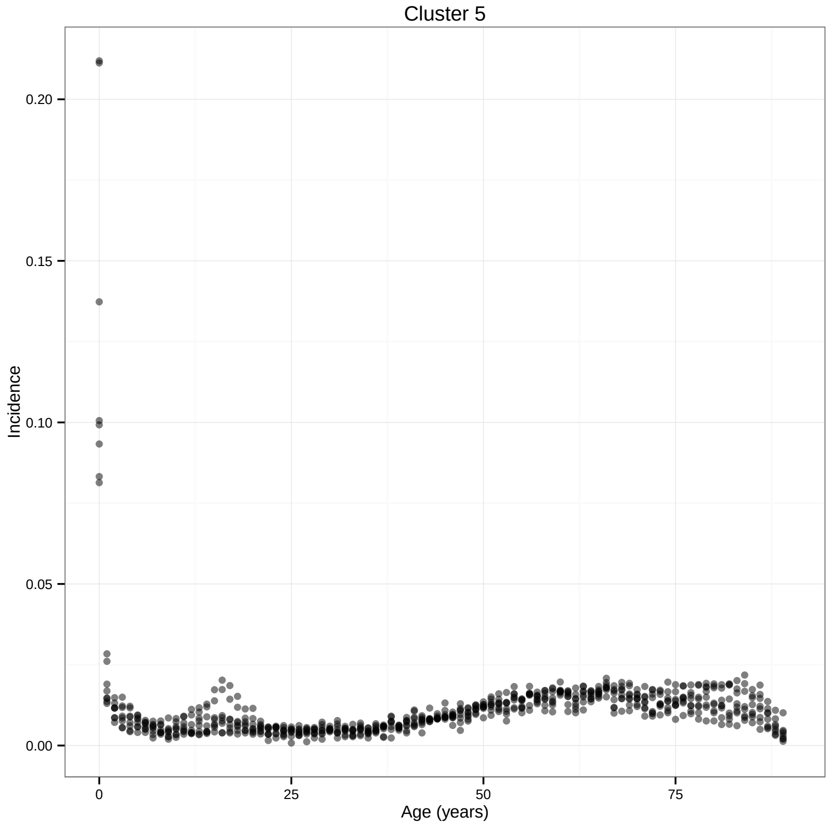

Cluster 5
Incidence
0.20
0.15
0.10
0.05
0.00
0
25
50
75
Age (years)

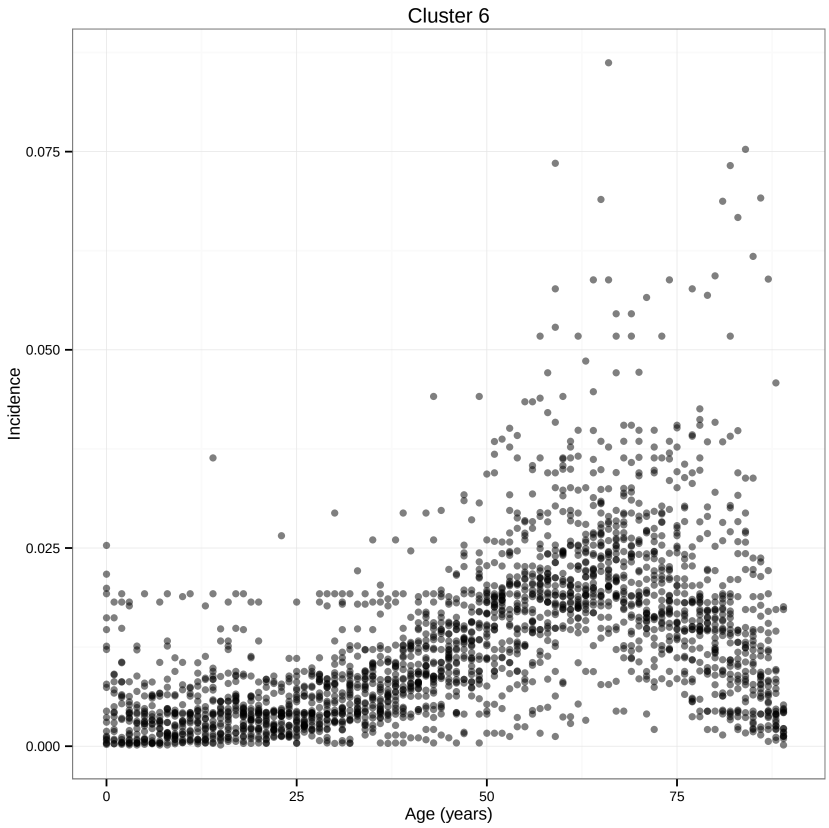
Cluster 6
Incidence
0.075
0.050
0.025
0.000
0
25
50
75
Age (years)

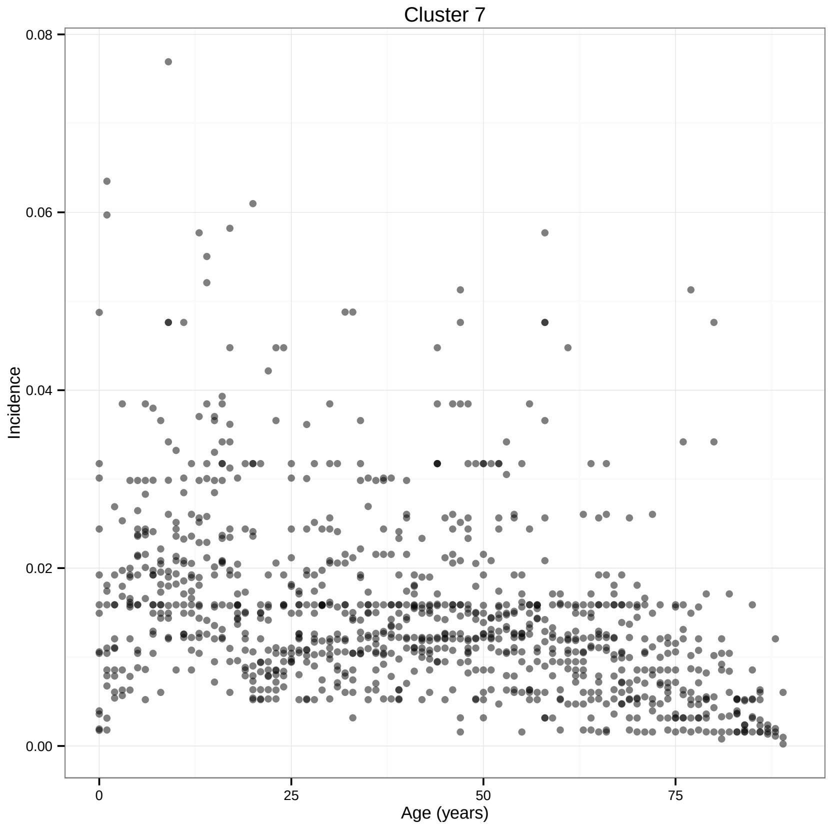
Cluster 7
Incidence
0.08
0.06
0.04
0.02
0.00
0
25
50
75
Age (years)

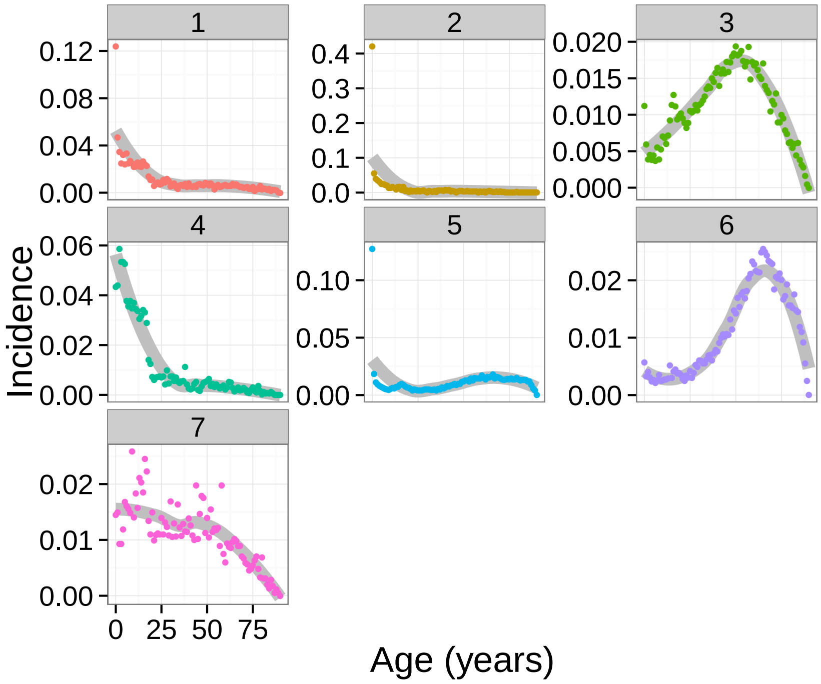
1
2
3
4
5
6
7
0.12
0.08
0.04
0.00
0.4
0.3
0.2
0.1
0.0
0.020
0.015
0.010
0.005
0.000
0.06
0.04
0.02
0.00
0.10
0.05
0.00
0.02
0.01
0.00
0.02
0.01
0.00
0
25
50
75
Incidence
Age (years)

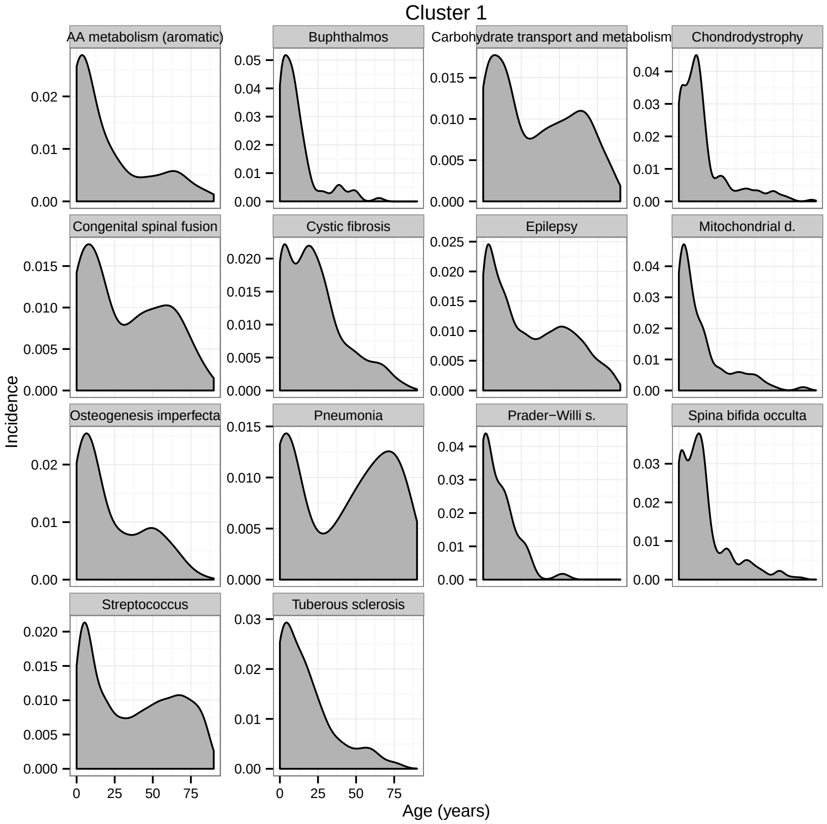
Cluster 1
AA metabolism (aromatic)
Buphthalmos
Carbohydrate transport and metabolism
Chondrodystrophy
Congenital spinal fusion
Cystic fibrosis
Epilepsy
Mitochondrial d.
Osteogenesis imperfecta
Pneumonia
Prader−Willi s.
Spina bifida occulta
Streptococcus
Tuberous sclerosis
Incidence
Age (years)

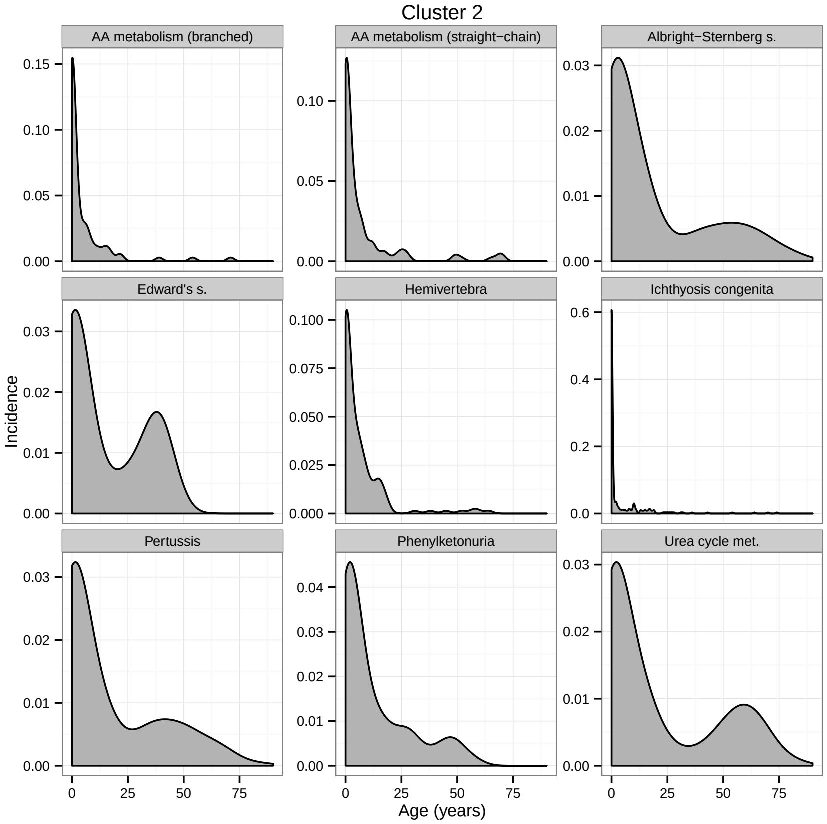
Cluster 2
AA metabolism (branched)
AA metabolism (straight−chain)
Albright−Sternberg s.
Edward's s.
Hemivertebra
Ichthyosis congenita
Pertussis
Phenylketonuria
Urea cycle met.
Incidence
Age (years)
0
25
50
75
0.00
0.05
0.10
0.15
0.01
0.02
0.03
0.000
0.025
0.050
0.075
0.100
0.0
0.2
0.4
0.6
0.04

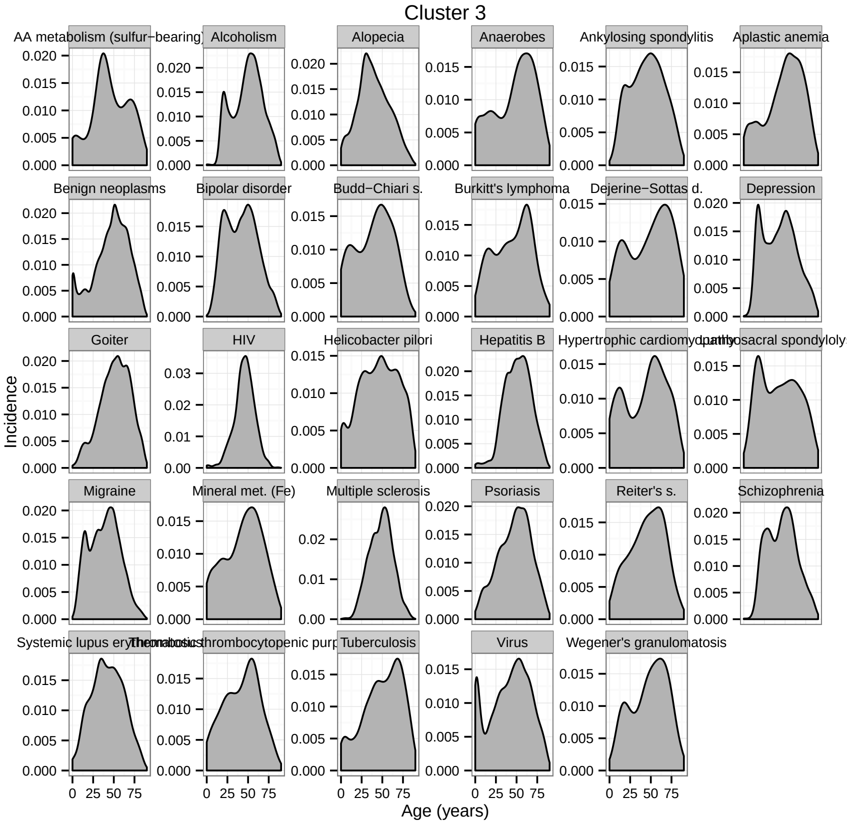
Cluster 3
AA metabolism (sulfur−bearing)
Alcoholism
Alopecia
Anaerobes
Ankylosing spondylitis
Aplastic anemia
Benign neoplasms
Bipolar disorder
Budd−Chiari s.
Burkitt's lymphoma
Dejerine−Sottas d.
Depression
Goiter
HIV
Helicobacter pilori
Hepatitis B
Migraine
Mineral met. (Fe)
Multiple sclerosis
Psoriasis
Reiter's s.
Schizophrenia
Tuberculosis
Virus
Wegener's granulomatosis
Incidence
Age (years)

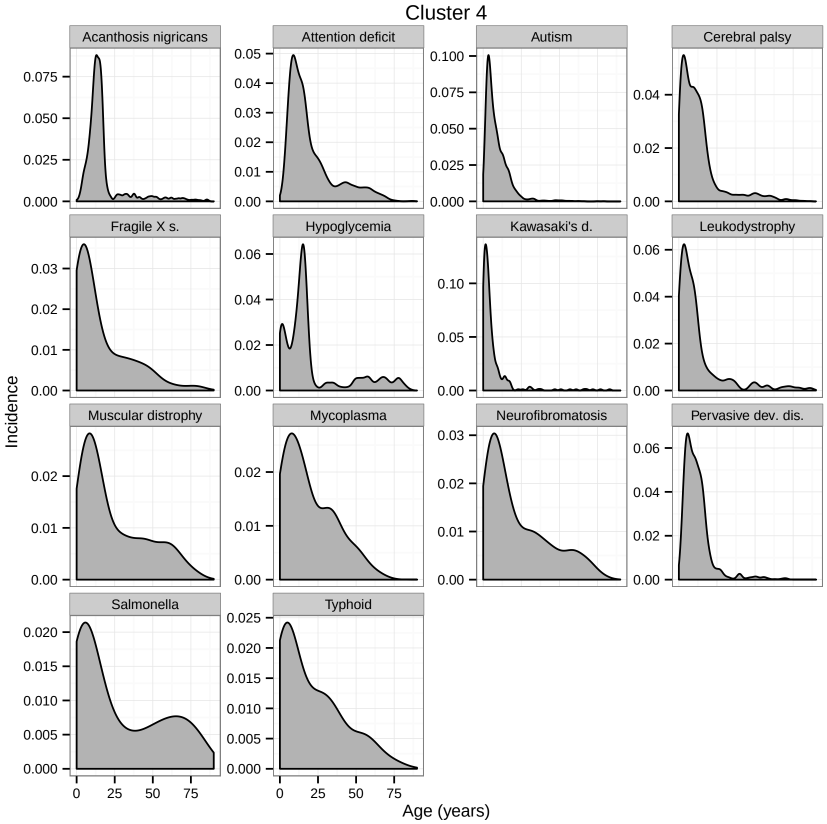
Cluster 4
Acanthosis nigricans
Attention deficit
Autism
Cerebral palsy
Fragile X s.
Hypoglycemia
Kawasaki's d.
Leukodystrophy
Muscular distrophy
Mycoplasma
Neurofibromatosis
Pervasive dev. dis.
Salmonella
Typhoid
Incidence
Age (years)

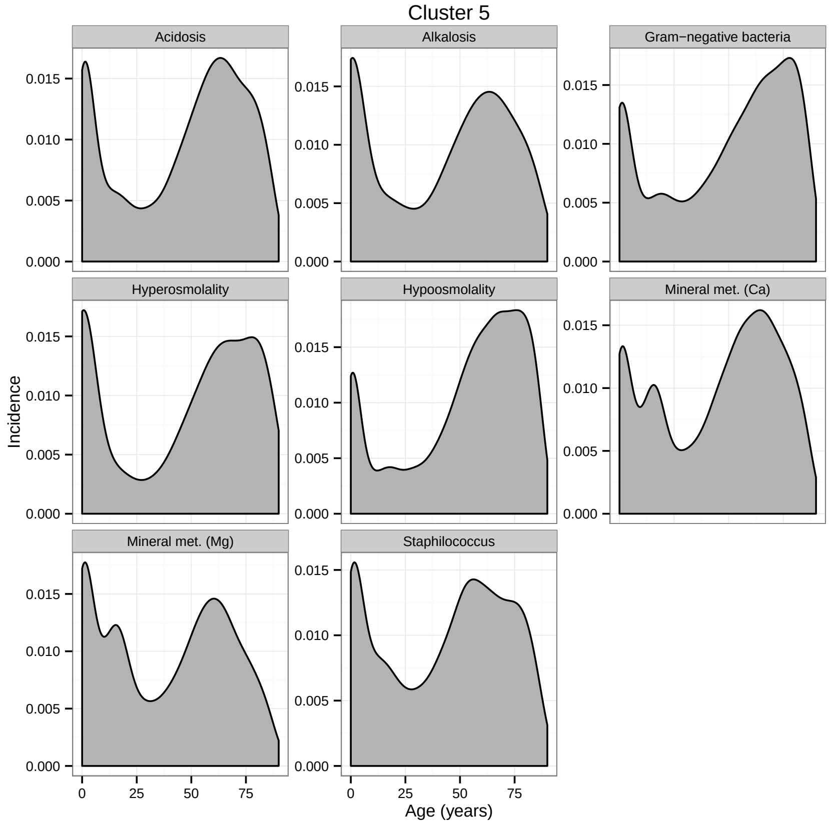

Cluster 5
Acidosis
Alkalosis
Gram−negative bacteria
Hyperosmolality
Hypoosmolality
Mineral met. (Ca)
Mineral met. (Mg)
Staphilococcus
Incidence
0.000
0.005
0.010
0.015
0
25
50
75
Age (years)

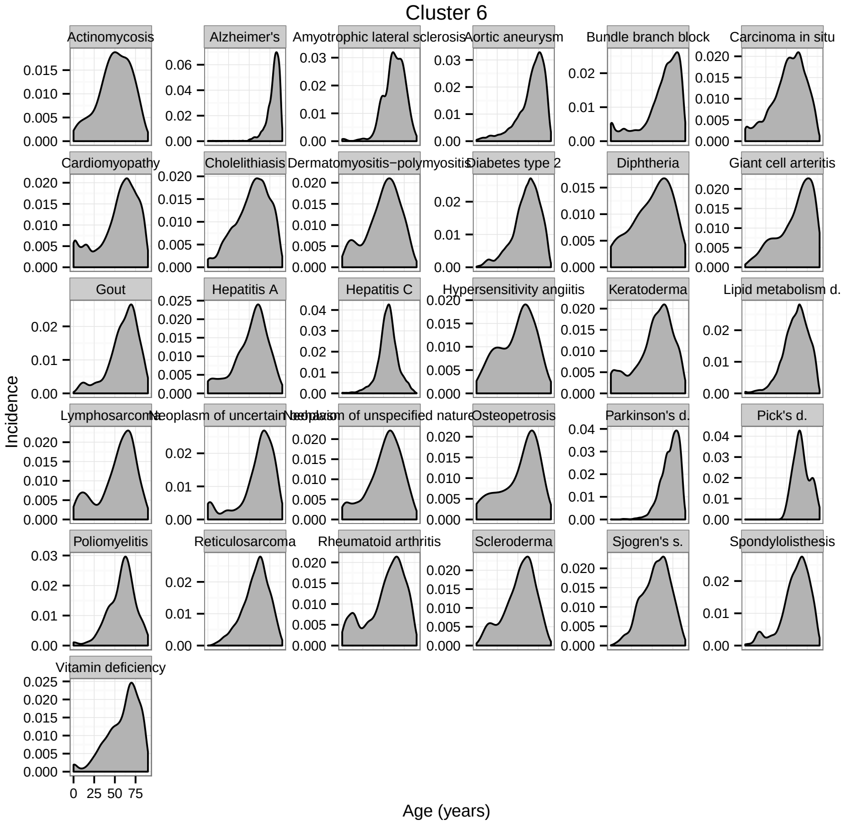
Cluster 6
Actinomycosis
Alzheimer's
Amyotrophic lateral sclerosis
Aortic aneurysm
Bundle branch block
Carcinoma in situ
Cardiomyopathy
Cholelithiasis
Dermatomyositis−polymyositis
Diabetes type 2
Diphtheria
Giant cell arteritis
Gout
Hepatitis A
Hepatitis C
Hypersensitivity angiitis
Keratoderma
Lipid metabolism d.
Lymphosarcoma
Neoplasm of uncertain behav.
Neoplasm of unspecified nature
Osteopetrosis
Parkinson's d.
Pick's d.
Poliomyelitis
Reticulosarcoma
Rheumatoid arthritis
Scleroderma
Sjogren's s.
Spondylolisthesis
Vitamin deficiency
Incidence
Age (years)
0 25 50 75

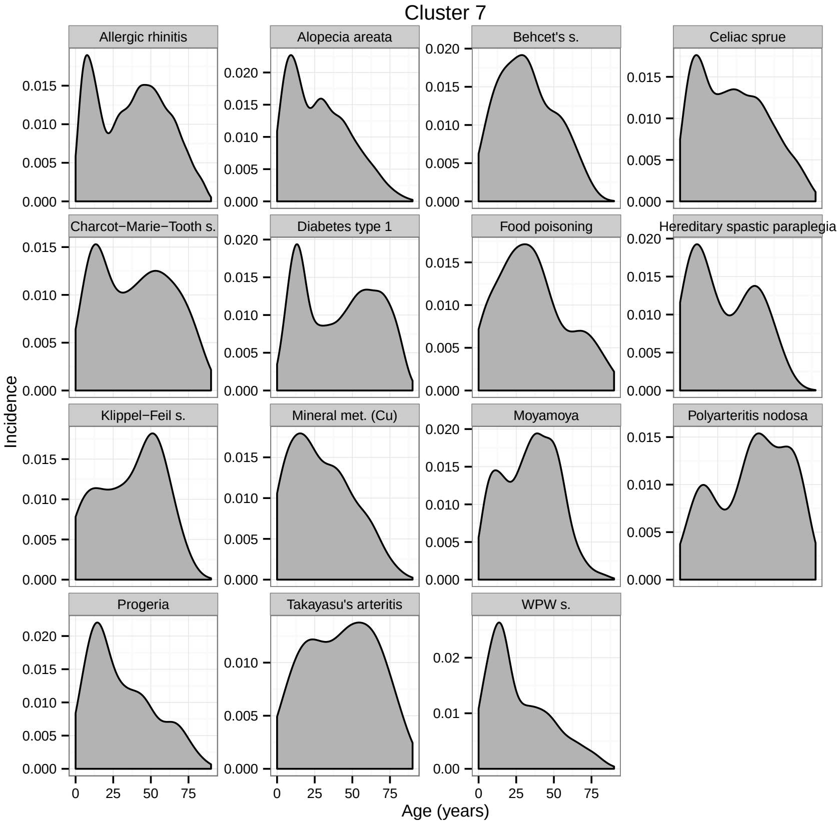
Cluster 7
Allergic rhinitis
Alopecia areata
Behcet's s.
Celiac sprue
Charcot−Marie−Tooth s.
Diabetes type 1
Food poisoning
Hereditary spastic paraplegia
Klippel−Feil s.
Mineral met. (Cu)
Moyamoya
Polyarteritis nodosa
Progeria
Takayasu's arteritis
WPW s.
Incidence
Age (years)
0
25
50
75

5.7 cluster size 8

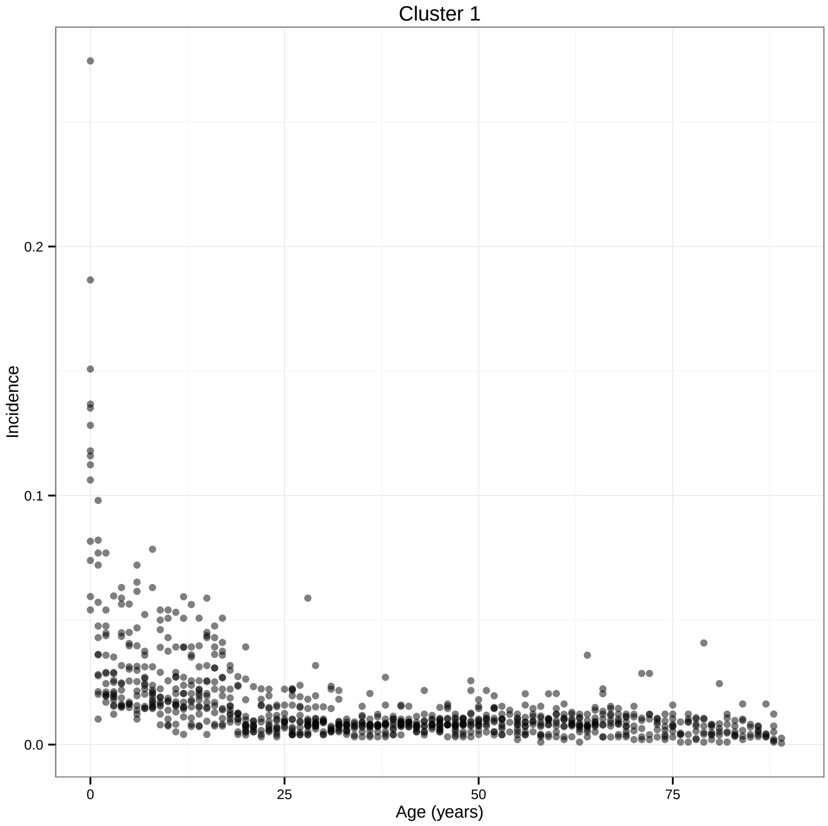

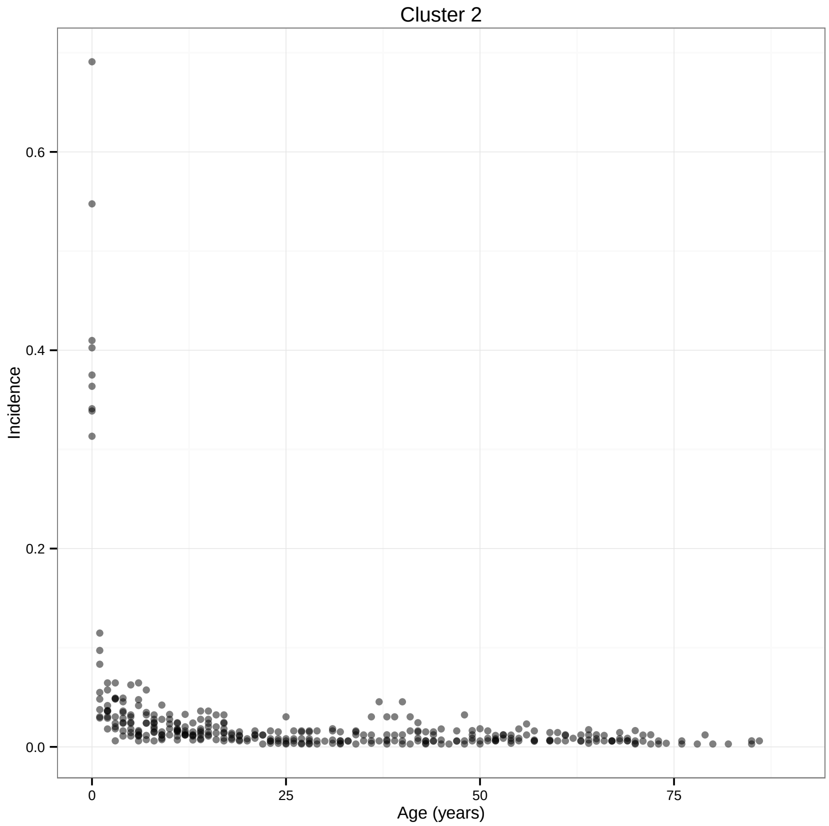
Cluster 2
Incidence
0.0
0.2
0.4
0.6
0
25
50
75
Age (years)

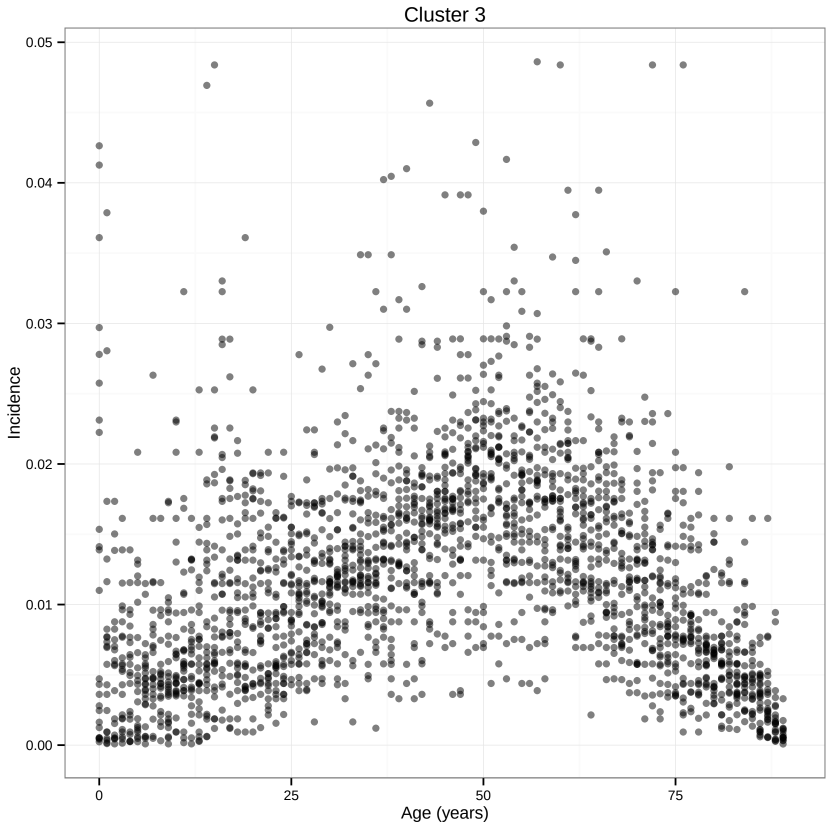
Cluster 3
Incidence
0.05
0.04
0.03
0.02
0.01
0.00
0
25
50
75
Age (years)

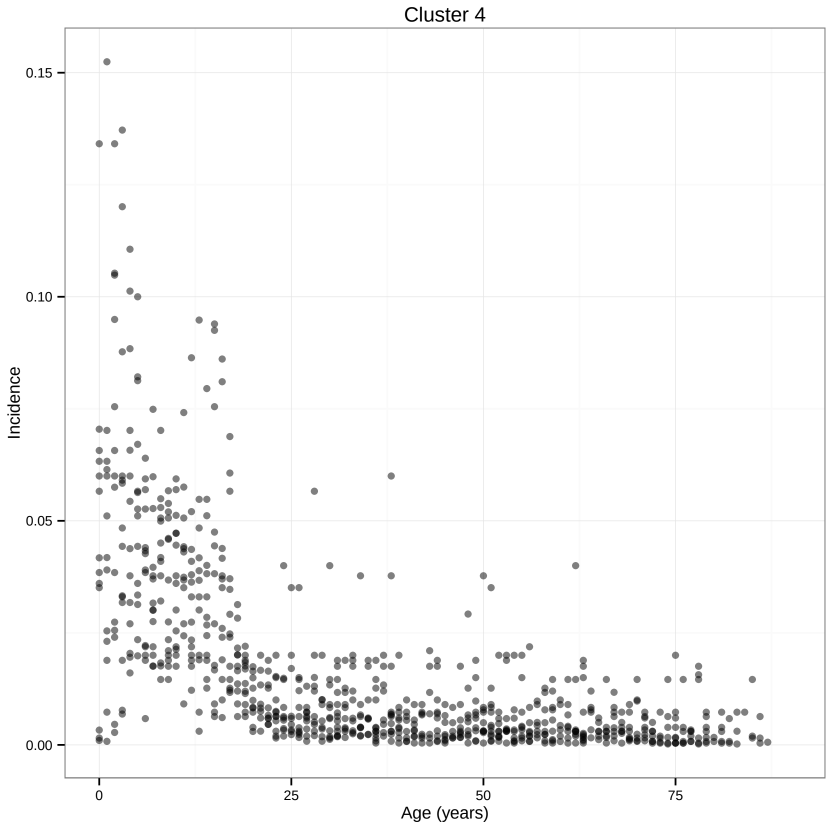
Cluster 4
Incidence
0.15
0.10
0.05
0.00
0
25
50
75
Age (years)

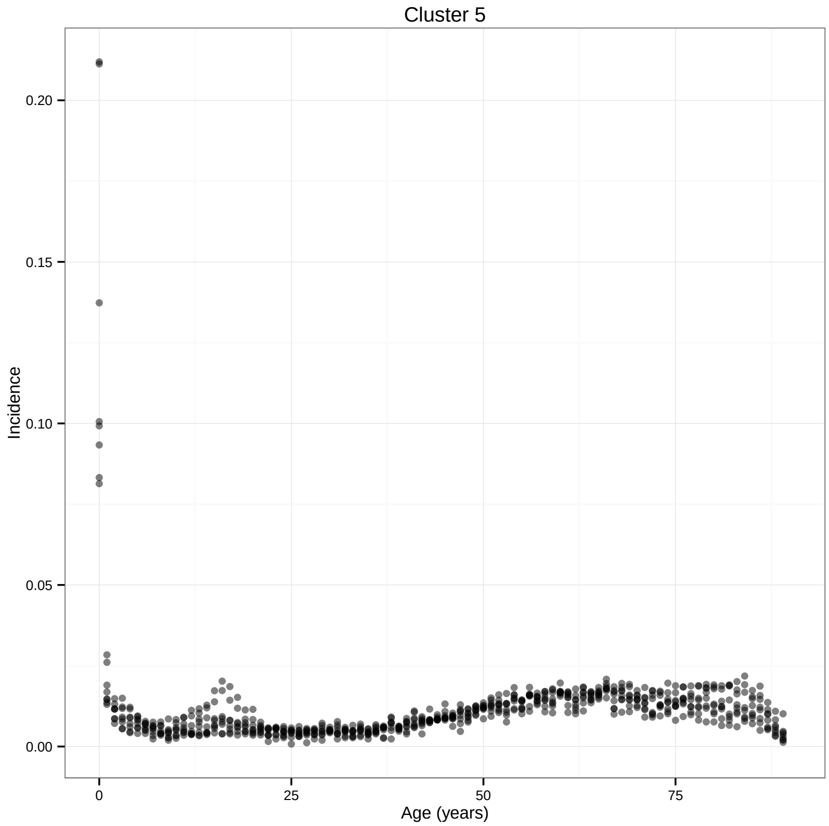
Cluster 5
0.20
0.15
0.10
0.05
0.00
Incidence
0
25
50
75
Age (years)

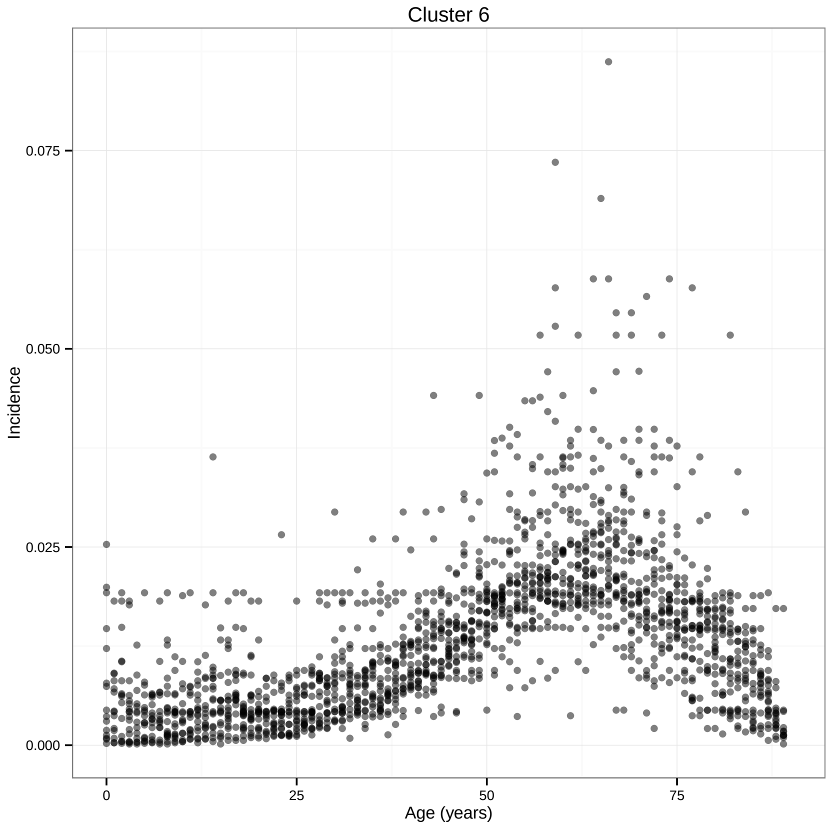
Cluster 6
Incidence
0.075
0.050
0.025
0.000
0
25
50
75
Age (years)

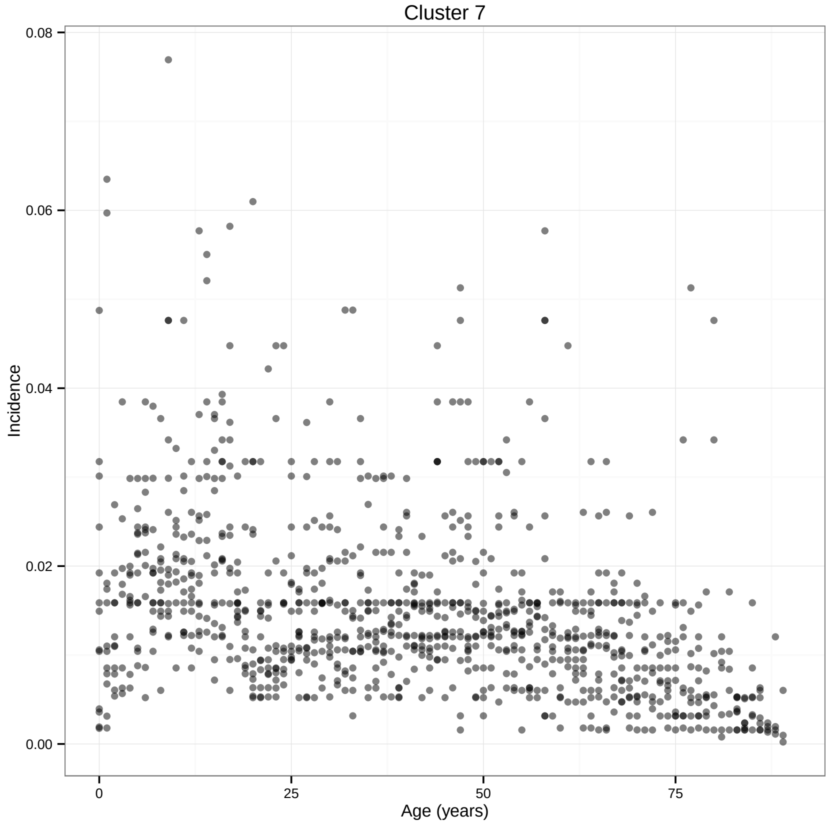
Cluster 7
Incidence
0.08
0.06
0.04
0.02
0.00
0
25
50
75
Age (years)

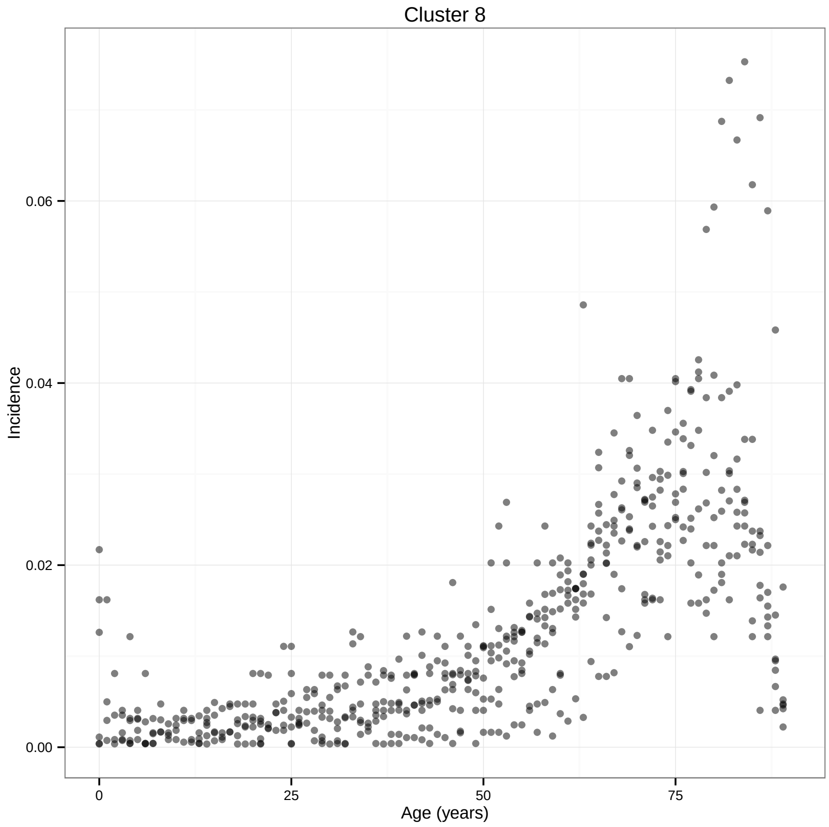

Cluster 8
Incidence
0.00
0.02
0.04
0.06
0
25
50
75
Age (years)

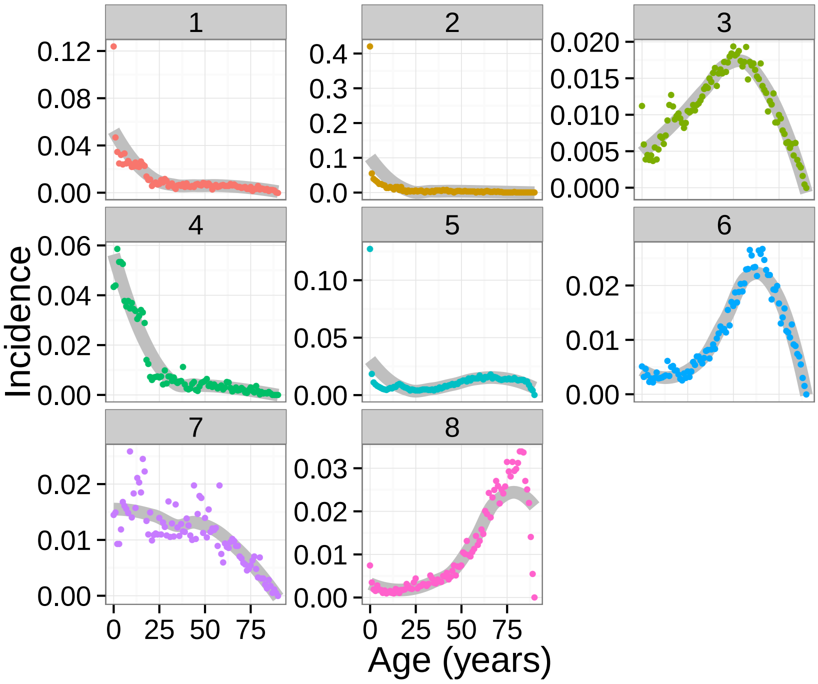

1
2
3
4
5
6
7
8
Incidence
Age (years)
0.12
0.08
0.04
0.00
0.4
0.3
0.2
0.1
0.0
0.020
0.015
0.010
0.005
0.000
0.06
0.04
0.02
0.00
0.10
0.05
0.00
0.02
0.01
0.00
0.02
0.01
0.00
0.03
0.02
0.01
0.00
0 25 50 75
0 25 50 75

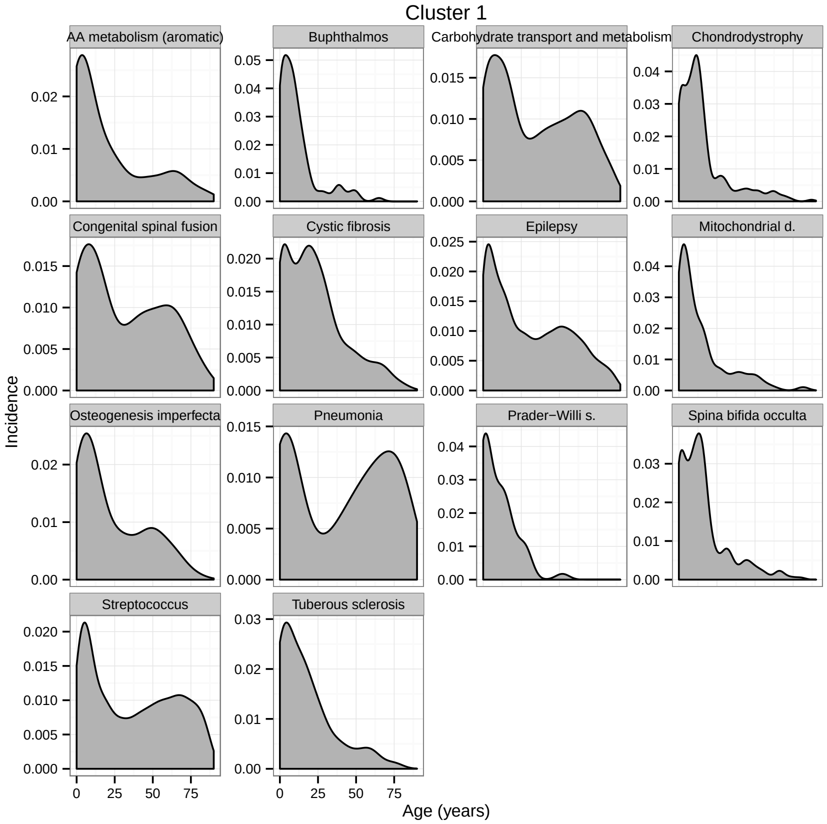
Cluster 1
AA metabolism (aromatic)
Buphthalmos
Carbohydrate transport and metabolism
Chondrodystrophy
Congenital spinal fusion
Cystic fibrosis
Epilepsy
Mitochondrial d.
Osteogenesis imperfecta
Pneumonia
Prader−Willi s.
Spina bifida occulta
Streptococcus
Tuberous sclerosis
Incidence
Age (years)

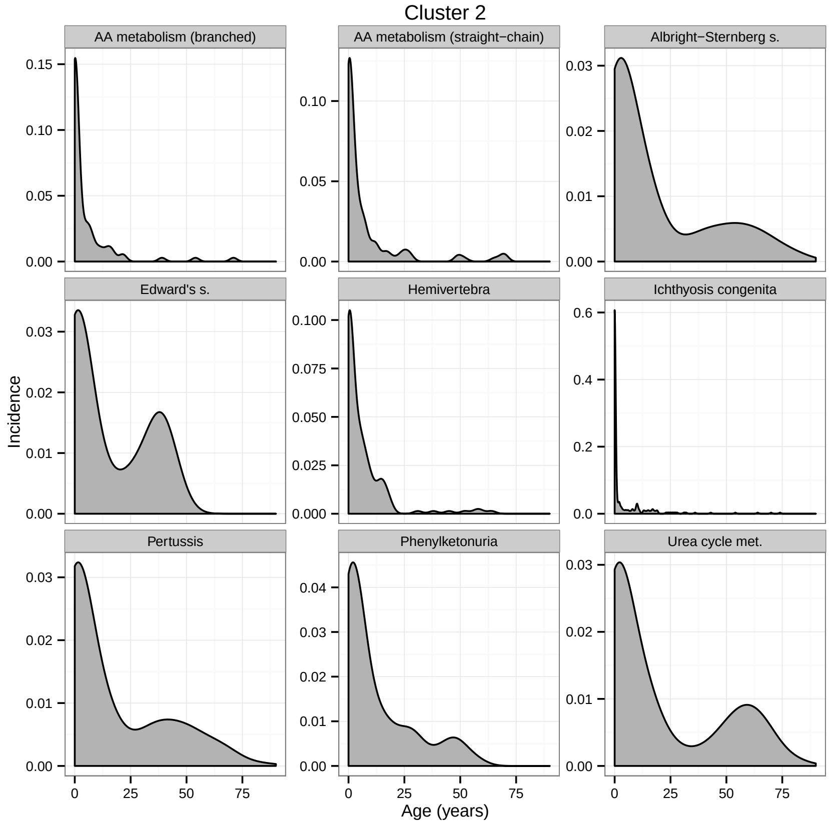
Cluster 2
AA metabolism (branched)
AA metabolism (straight−chain)
Albright−Sternberg s.
Edward's s.
Hemivertebra
Ichthyosis congenita
Pertussis
Phenylketonuria
Urea cycle met.
Incidence
Age (years)
0
25
50
75

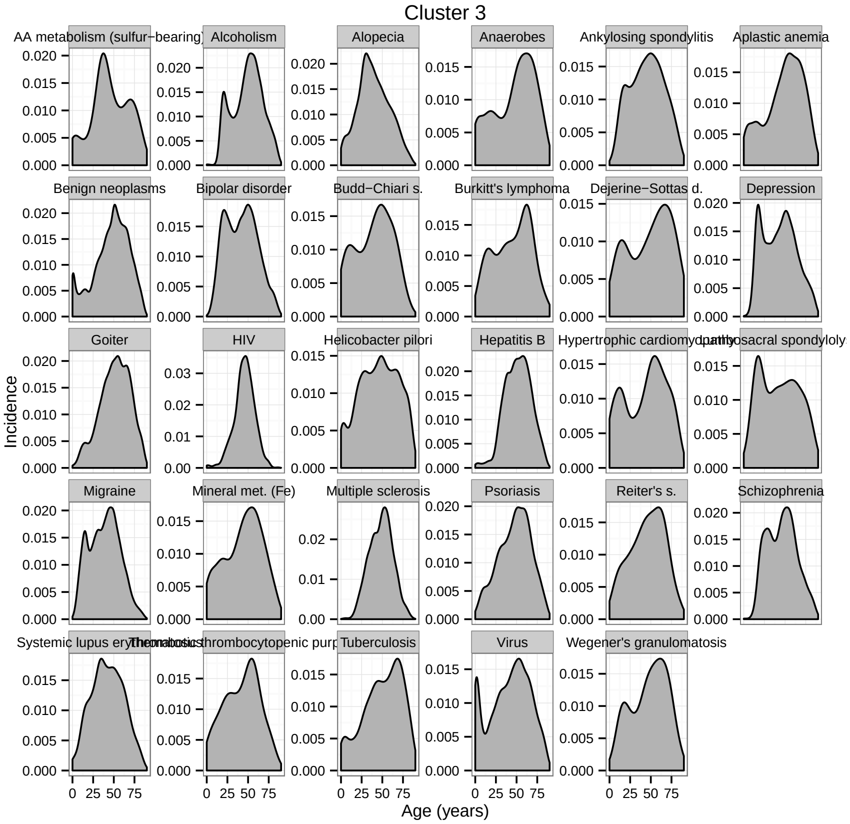
Cluster 3
AA metabolism (sulfur−bearing)
Alcoholism
Alopecia
Anaerobes
Ankylosing spondylitis
Aplastic anemia
Benign neoplasms
Bipolar disorder
Budd−Chiari s.
Burkitt's lymphoma
Dejerine−Sottas d.
Depression
Goiter
HIV
Helicobacter pilori
Hepatitis B
Hypertrophic cardiomyopathy
Lumbosacral spondyloly
Migraine
Mineral met. (Fe)
Multiple sclerosis
Psoriasis
Reiter's s.
Schizophrenia
Systemic lupus erythematosus
Thrombotic thrombocytopenic purp
Tuberculosis
Virus
Wegener's granulomatosis
Incidence
Age (years)

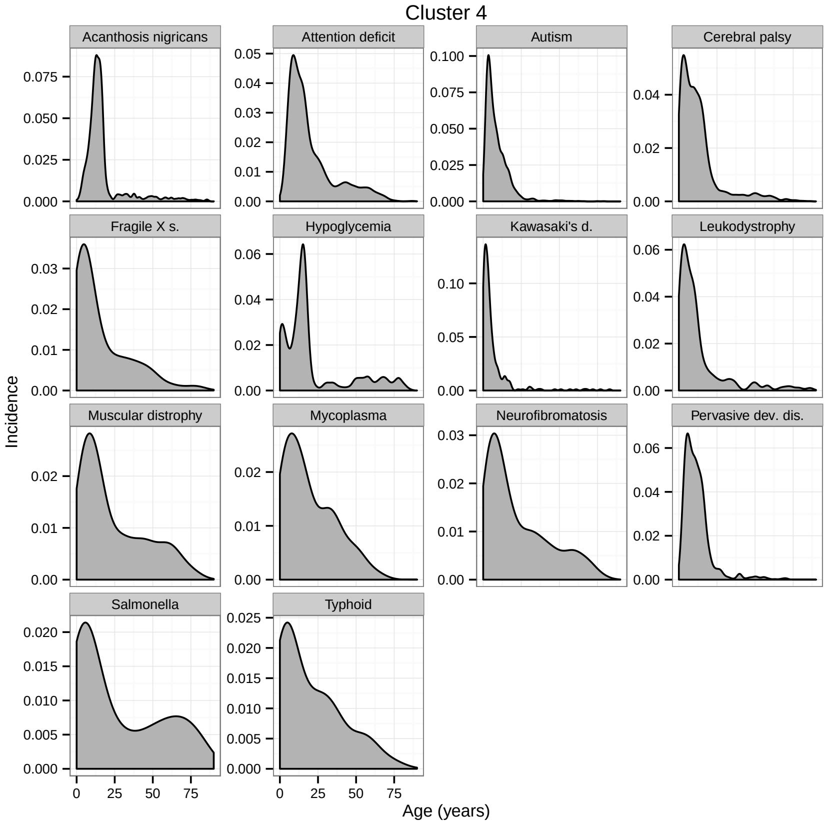
Cluster 4
Acanthosis nigricans
Attention deficit
Autism
Cerebral palsy
Fragile X s.
Hypoglycemia
Kawasaki's d.
Leukodystrophy
Muscular distrophy
Mycoplasma
Neurofibromatosis
Pervasive dev. dis.
Salmonella
Typhoid
Incidence
Age (years)

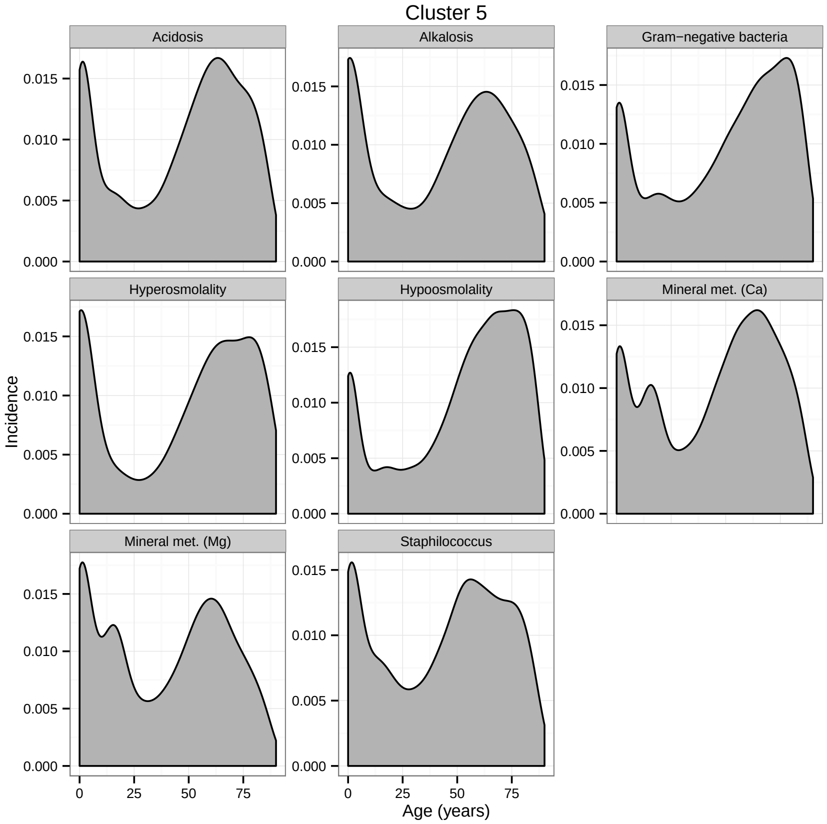

Cluster 5
Acidosis
Alkalosis
Gram−negative bacteria
Hyperosmolality
Hypoosmolality
Mineral met. (Ca)
Mineral met. (Mg)
Staphilococcus
Incidence
Age (years)
0.000
0.005
0.010
0.015
0
25
50
75

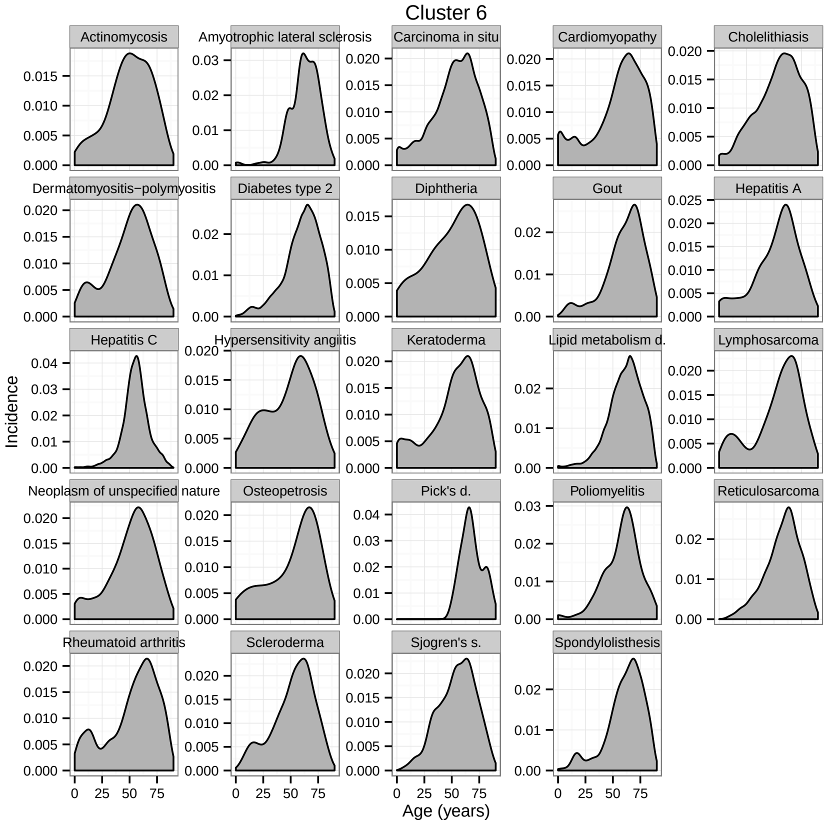
Cluster 6
Actinomycosis
Amyotrophic lateral sclerosis
Carcinoma in situ
Cardiomyopathy
Cholelithiasis
Dermatomyositis−polymyositis
Diabetes type 2
Diphtheria
Gout
Hepatitis A
Hepatitis C
Hypersensitivity angiitis
Keratoderma
Lipid metabolism d.
Lymphosarcoma
Neoplasm of unspecified nature
Osteopetrosis
Pick's d.
Poliomyelitis
Reticulosarcoma
Rheumatoid arthritis
Scleroderma
Sjogren's s.
Spondylolisthesis
Incidence
Age (years)

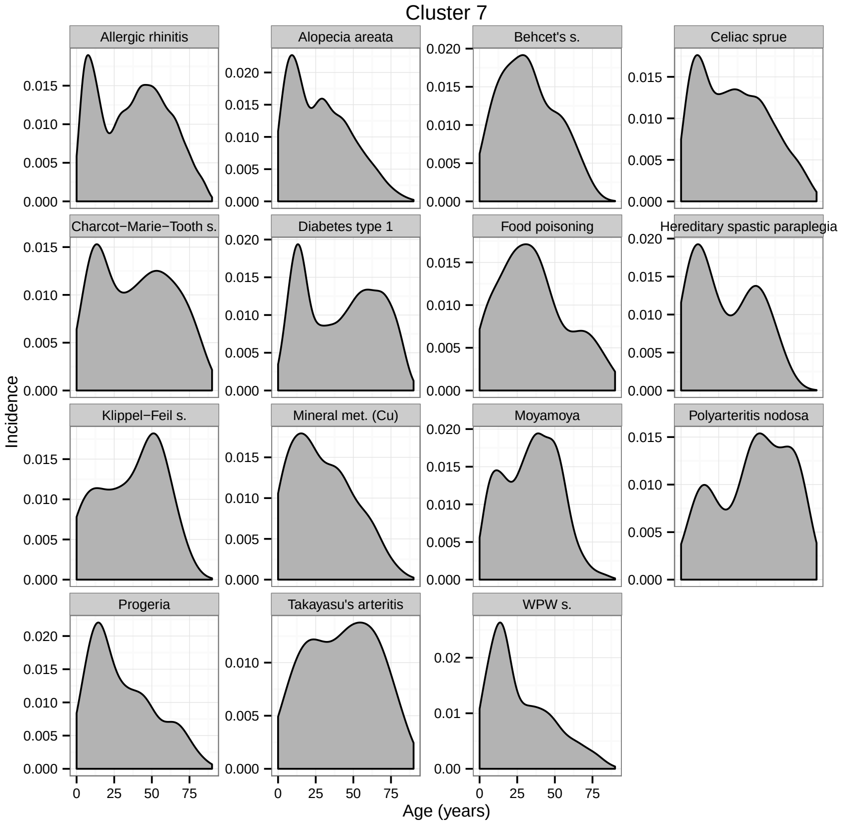
Cluster 7
Allergic rhinitis
Alopecia areata
Behcet's s.
Celiac sprue
Charcot−Marie−Tooth s.
Diabetes type 1
Food poisoning
Hereditary spastic paraplegia
Klippel−Feil s.
Mineral met. (Cu)
Moyamoya
Polyarteritis nodosa
Progeria
Takayasu's arteritis
WPW s.
Incidence
Age (years)

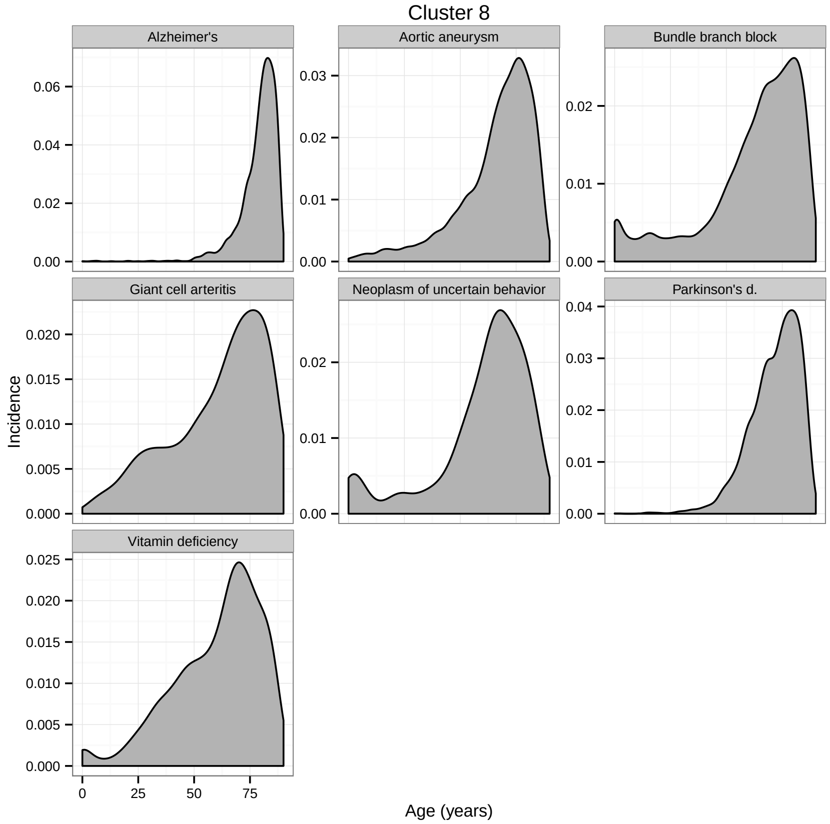
Cluster 8
Alzheimer's
Aortic aneurysm
Bundle branch block
Giant cell arteritis
Neoplasm of uncertain behavior
Parkinson's d.
Vitamin deficiency
Incidence
Age (years)
0.06
0.04
0.02
0.00
0.03
0.01
0.020
0.015
0.010
0.005
0.000
0.025
0.04
0
25
50
75

5.8 cluster size 9

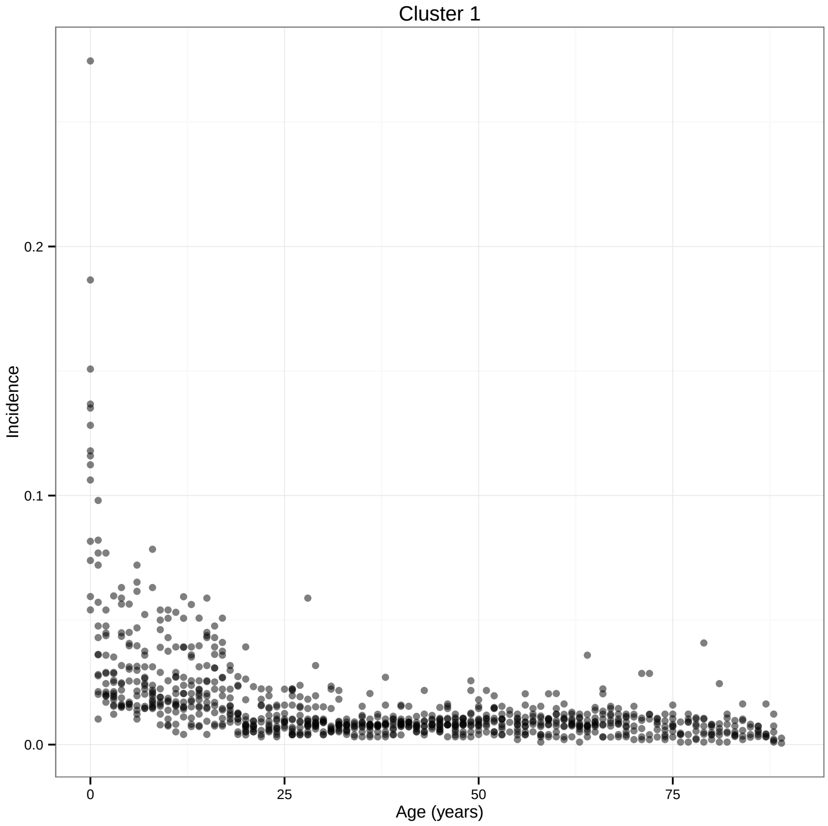

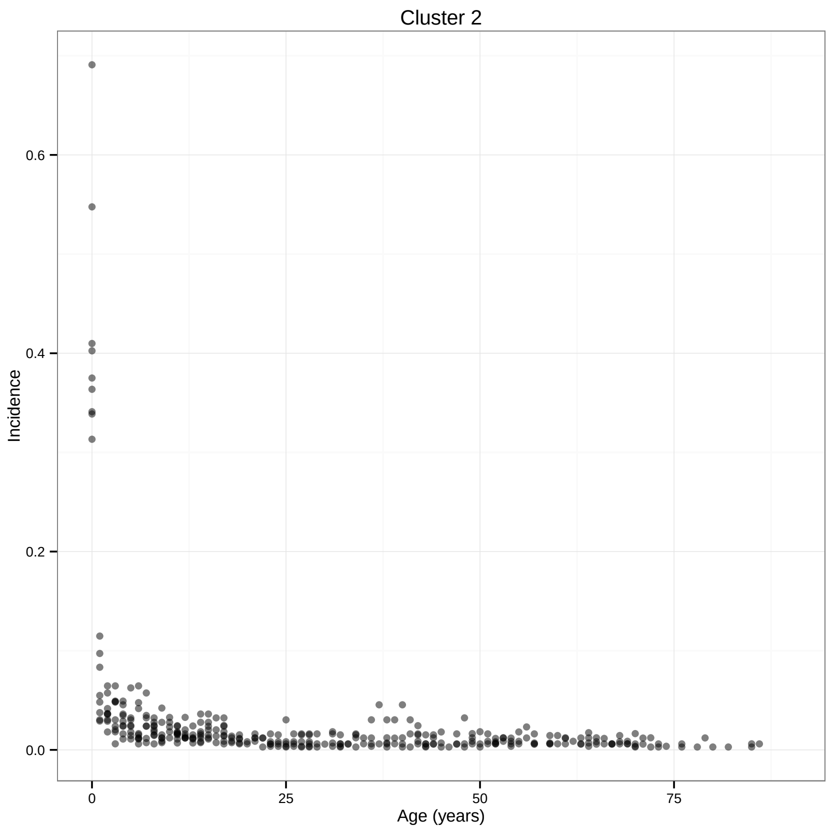
Cluster 2
Incidence
0.6
0.4
0.2
0.0
0
25
50
75
Age (years)

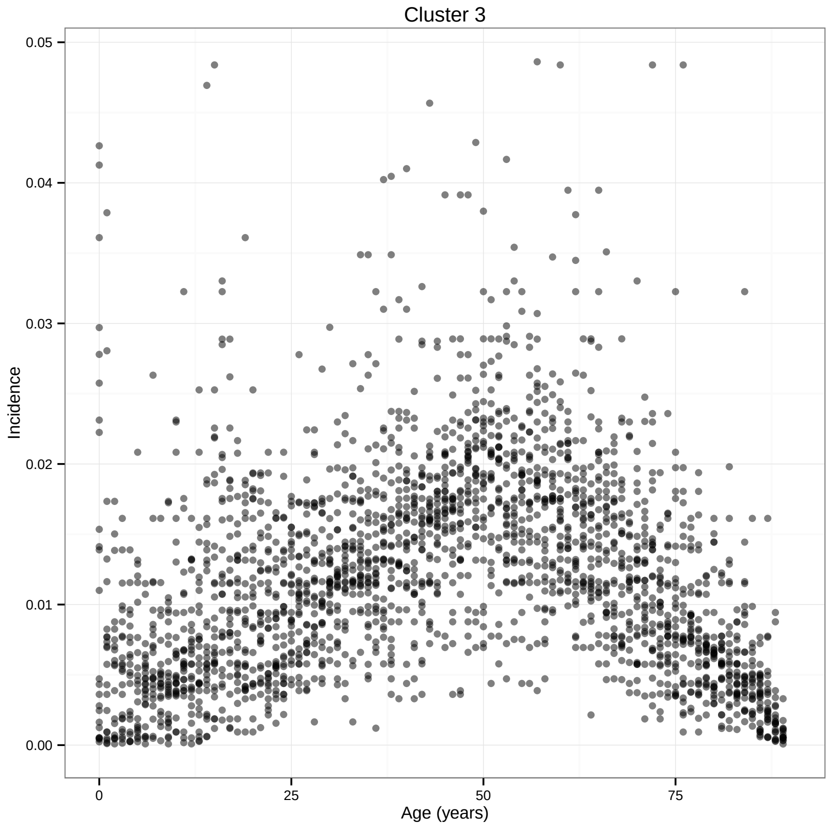
Cluster 3
Incidence
0.05
0.04
0.03
0.02
0.01
0.00
0
25
50
75
Age (years)

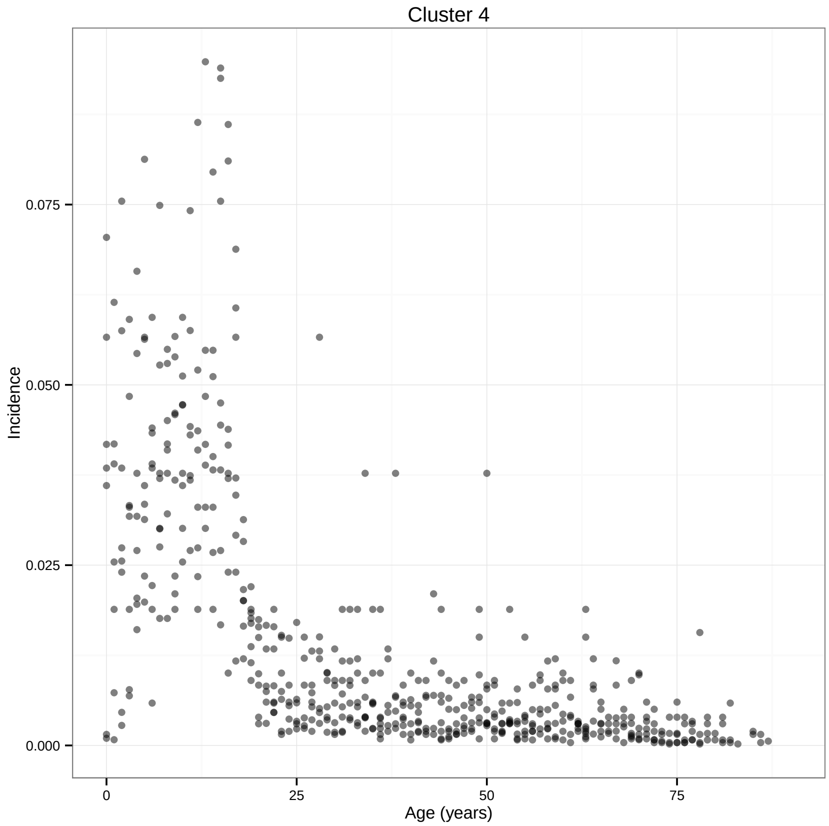

Cluster 4
Incidence
0.075
0.050
0.025
0.000
0
25
50
75
Age (years)

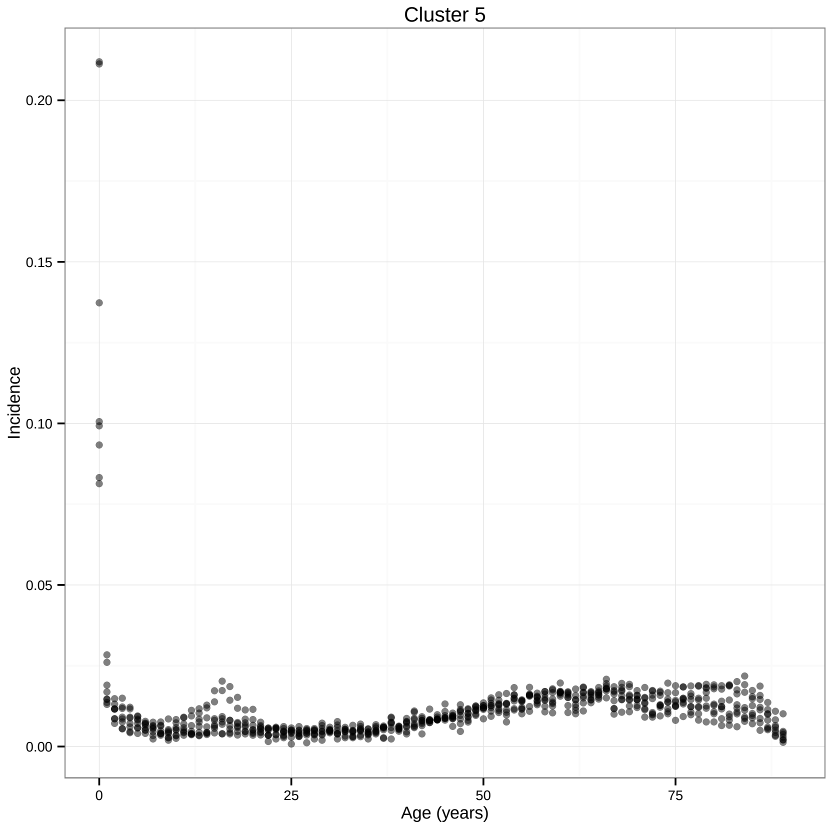

Cluster 5
Incidence
0.20
0.15
0.10
0.05
0.00
0
25
50
75
Age (years)

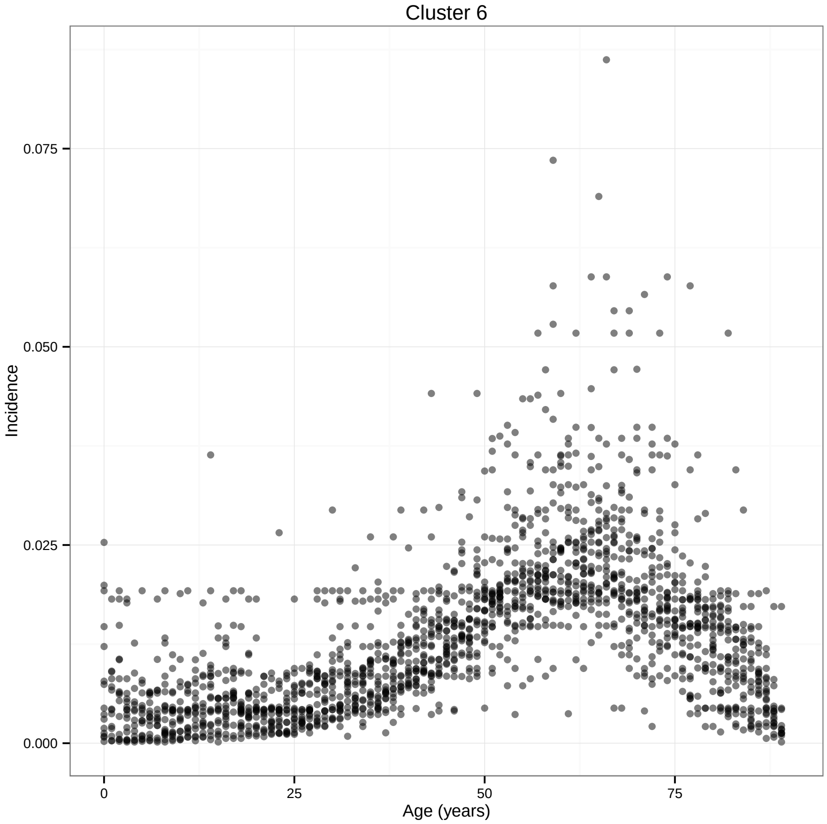
Cluster 6
Incidence
0.075
0.050
0.025
0.000
0
25
50
75
Age (years)

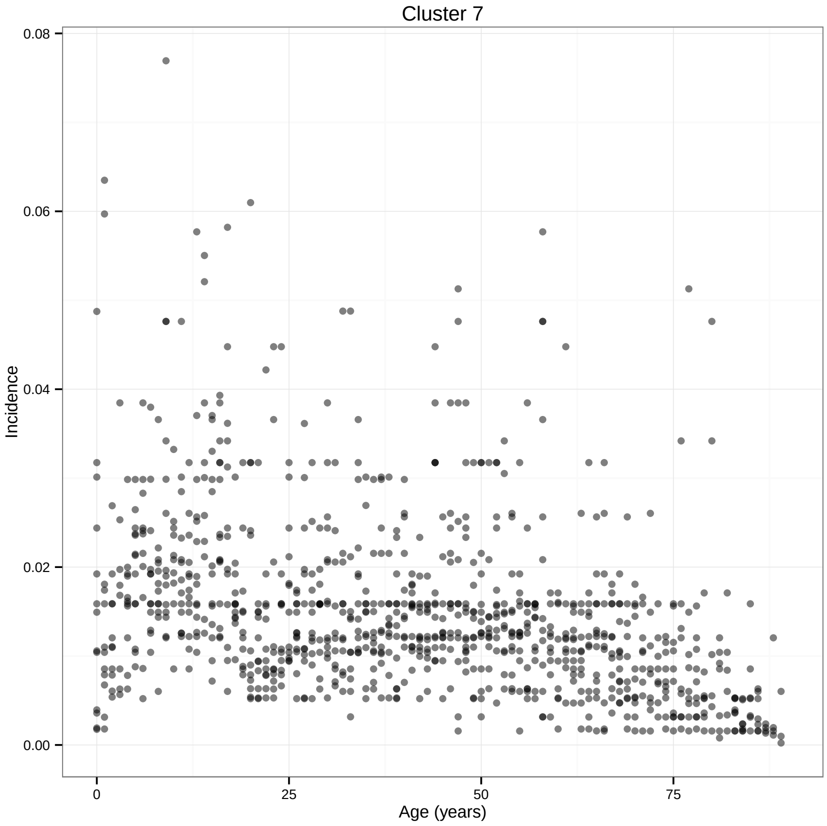
Cluster 7
0.08
0.06
0.04
0.02
0.00
Incidence
0
25
50
75
Age (years)

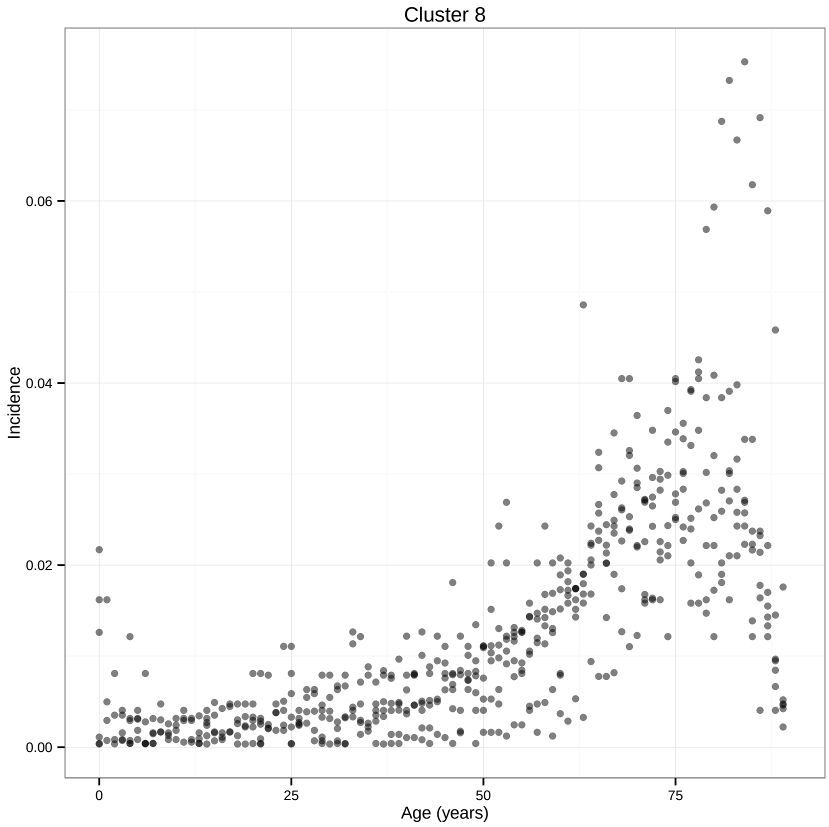
Cluster 8
Incidence
0.06
0.04
0.02
0.00
0
25
50
75
Age (years)

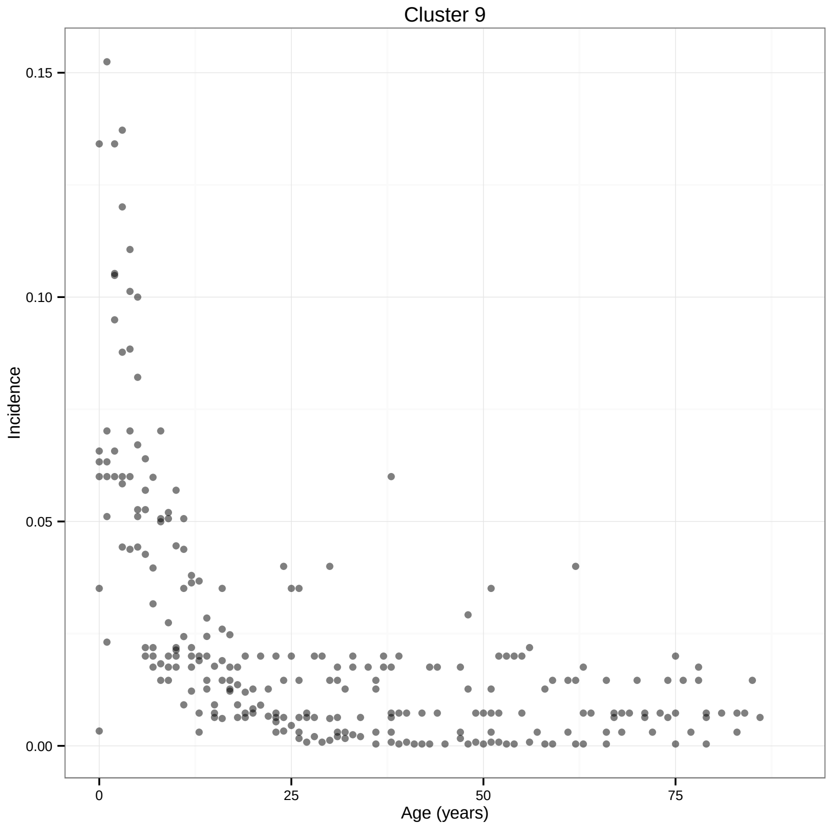
Cluster 9
Incidence
0.15
0.10
0.05
0.00
0
25
50
75
Age (years)

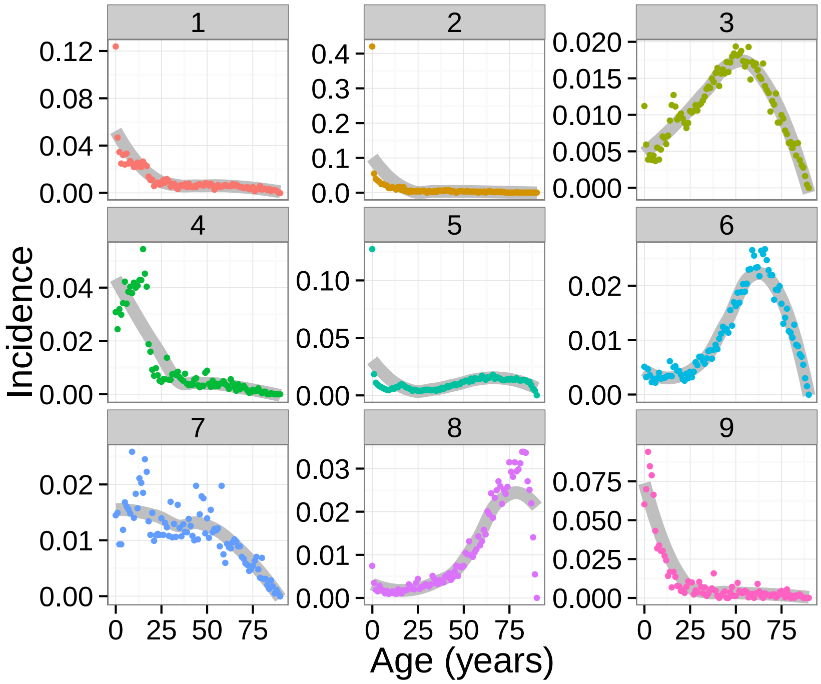
1
2
3
4
5
6
7
8
9
0.12
0.08
0.04
0.00
0.4
0.3
0.2
0.1
0.0
0.020
0.015
0.010
0.005
0.000
0.04
0.02
0.00
0.10
0.05
0.00
0.02
0.01
0.00
0.02
0.01
0.00
0.03
0.02
0.01
0.00
0.075
0.050
0.025
0.000
Incidence
0 25 50 75
Age (years)

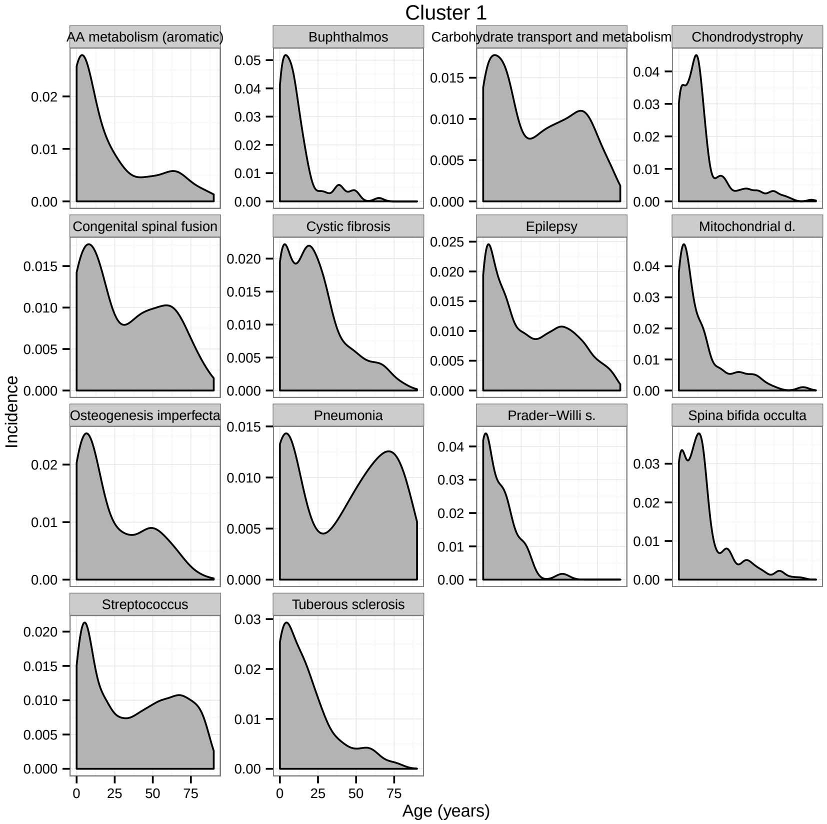
Cluster 1
AA metabolism (aromatic)
Buphthalmos
Carbohydrate transport and metabolism
Chondrodystrophy
Congenital spinal fusion
Cystic fibrosis
Epilepsy
Mitochondrial d.
Osteogenesis imperfecta
Pneumonia
Prader−Willi s.
Spina bifida occulta
Streptococcus
Tuberous sclerosis
Incidence
Age (years)

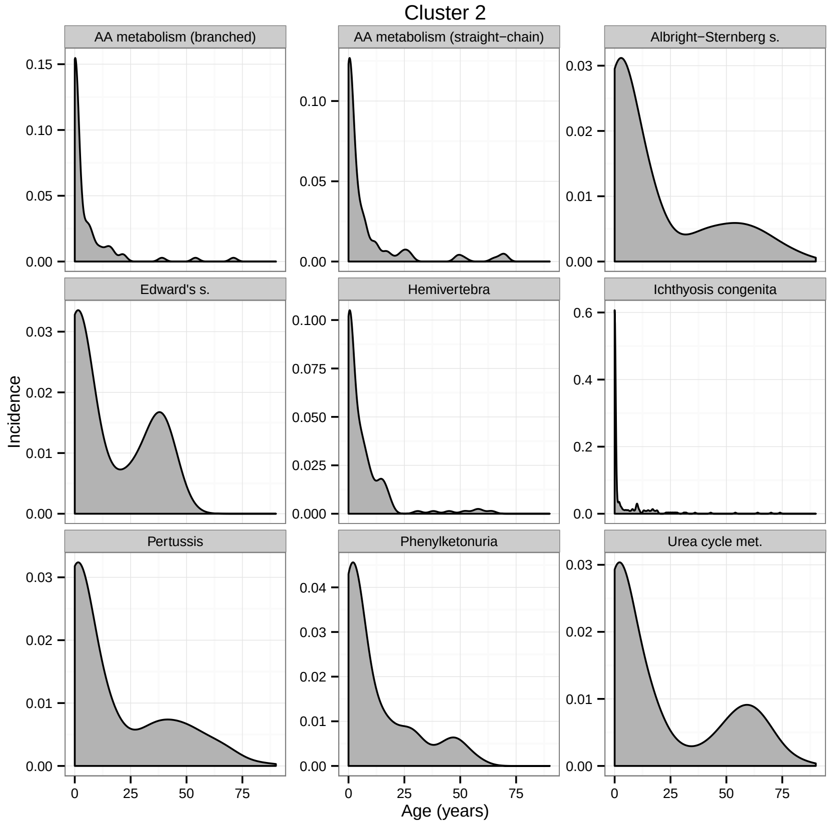
Cluster 2
AA metabolism (branched)
AA metabolism (straight−chain)
Albright−Sternberg s.
Edward's s.
Hemivertebra
Ichthyosis congenita
Pertussis
Phenylketonuria
Urea cycle met.
Incidence
Age (years)

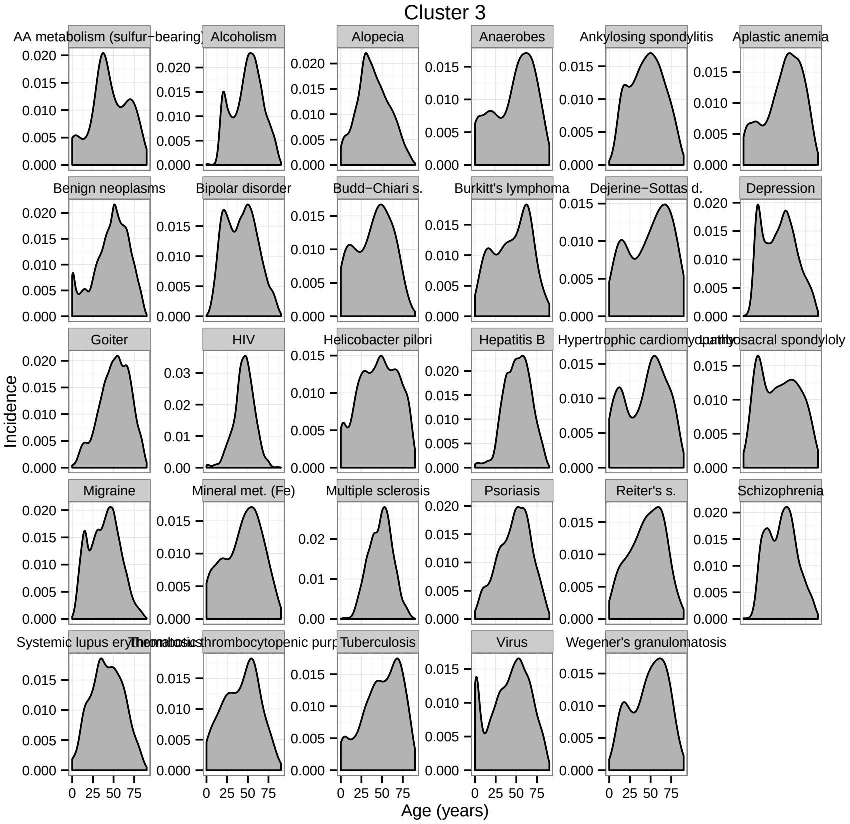
Cluster 3
AA metabolism (sulfur−bearing)
Alcoholism
Alopecia
Anaerobes
Ankylosing spondylitis
Aplastic anemia
Benign neoplasms
Bipolar disorder
Budd−Chiari s.
Burkitt's lymphoma
Dejerine−Sottas d.
Depression
Goiter
HIV
Helicobacter pilori
Hepatitis B
Migraine
Mineral met. (Fe)
Multiple sclerosis
Psoriasis
Reiter's s.
Schizophrenia
Tuberculosis
Virus
Wegener's granulomatosis
Incidence
Age (years)

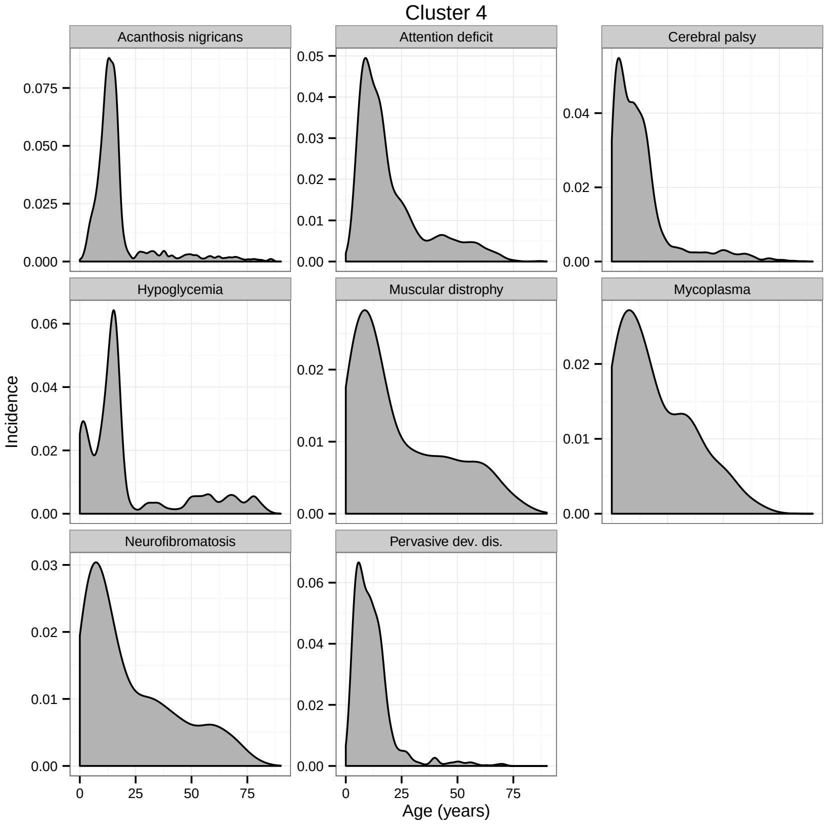

Cluster 4
Acanthosis nigricans
Attention deficit
Cerebral palsy
Hypoglycemia
Muscular distrophy
Mycoplasma
Neurofibromatosis
Pervasive dev. dis.
Incidence
Age (years)

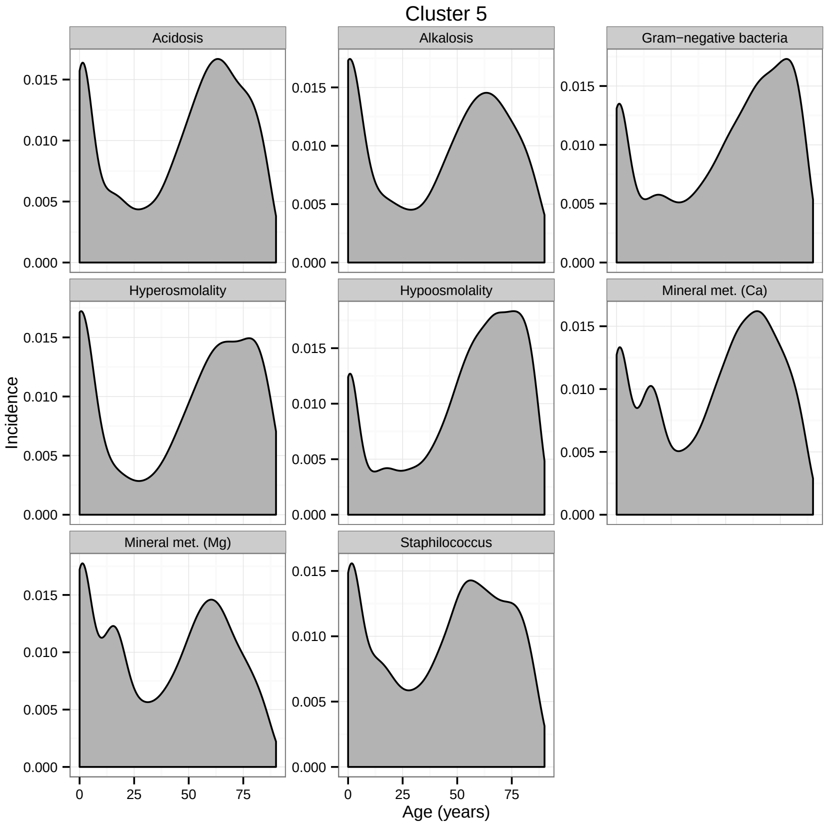

Cluster 5
Acidosis
Alkalosis
Gram−negative bacteria
Hyperosmolality
Hypoosmolality
Mineral met. (Ca)
Mineral met. (Mg)
Staphilococcus
Incidence
0.000
0.005
0.010
0.015
0
25
50
75
Age (years)

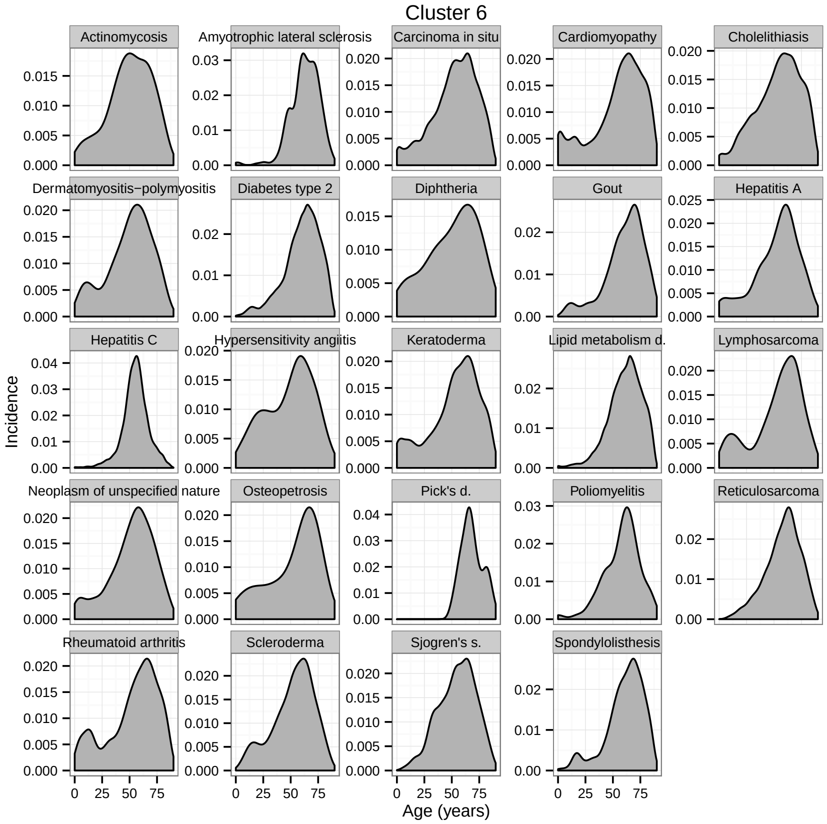
Cluster 6
Actinomycosis
Amyotrophic lateral sclerosis
Carcinoma in situ
Cardiomyopathy
Cholelithiasis
Dermatomyositis−polymyositis
Diabetes type 2
Diphtheria
Gout
Hepatitis A
Hepatitis C
Hypersensitivity angiitis
Keratoderma
Lipid metabolism d.
Lymphosarcoma
Neoplasm of unspecified nature
Osteopetrosis
Pick's d.
Poliomyelitis
Reticulosarcoma
Rheumatoid arthritis
Scleroderma
Sjogren's s.
Spondylolisthesis
Incidence
Age (years)

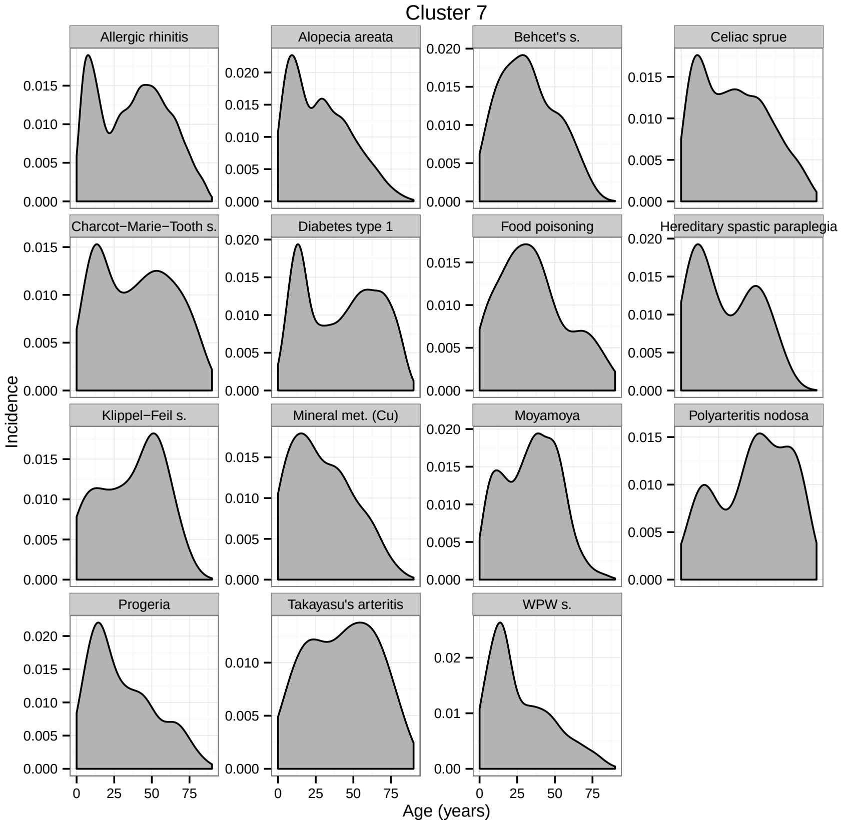
Cluster 7
Allergic rhinitis
Alopecia areata
Behcet's s.
Celiac sprue
Charcot−Marie−Tooth s.
Diabetes type 1
Food poisoning
Hereditary spastic paraplegia
Klippel−Feil s.
Mineral met. (Cu)
Moyamoya
Polyarteritis nodosa
Progeria
Takayasu's arteritis
WPW s.
Incidence
Age (years)

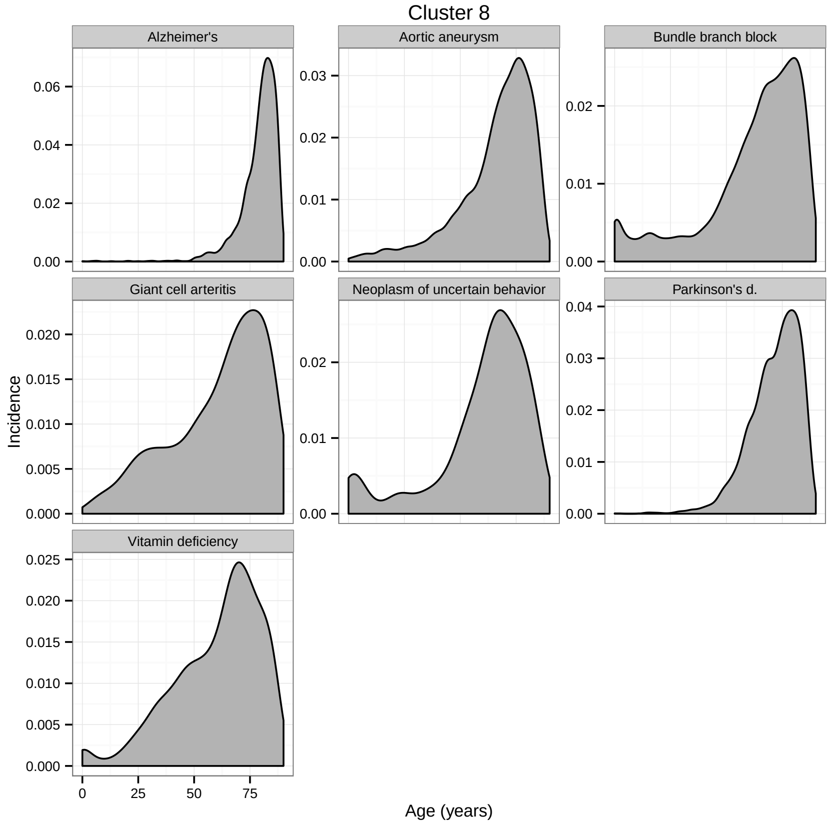
Cluster 8
Alzheimer's
Aortic aneurysm
Bundle branch block
Giant cell arteritis
Neoplasm of uncertain behavior
Parkinson's d.
Vitamin deficiency
Incidence
Age (years)
0
25
50
75
0.00
0.02
0.04
0.06
0.00
0.01
0.02
0.03
0.000
0.005
0.010
0.015
0.020
0.025
0.04

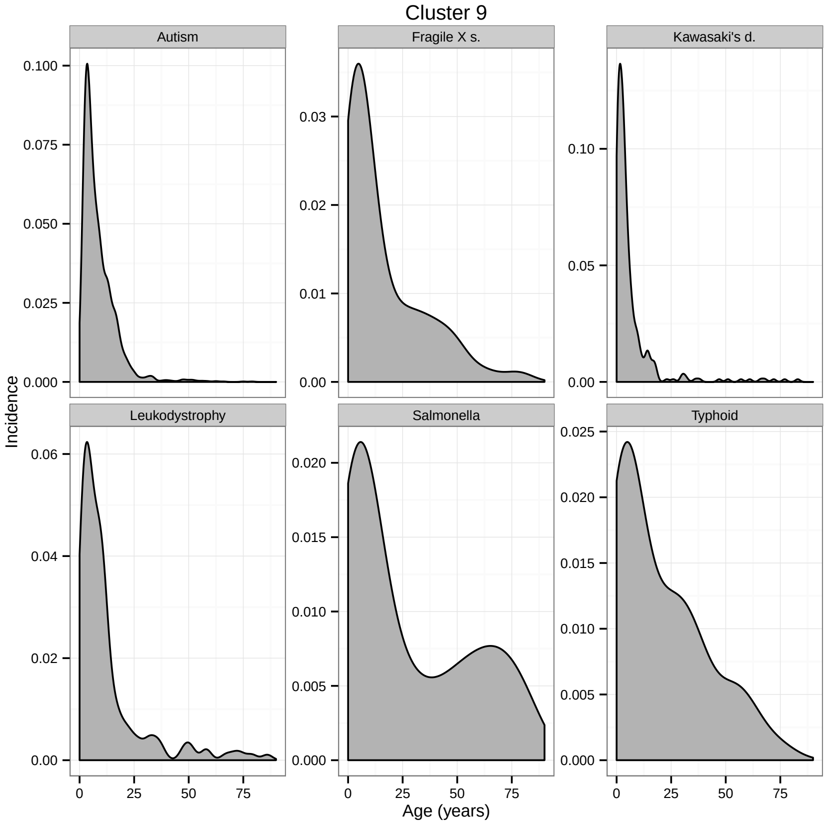

Cluster 9
Autism
Fragile X s.
Kawasaki's d.
Leukodystrophy
Salmonella
Typhoid
Incidence
Age (years)
0.100
0.075
0.050
0.025
0.000
0.03
0.02
0.01
0.00
0.10
0.05
0.00
0.06
0.04
0.02
0.00
0.020
0.015
0.010
0.005
0.000
0.025
0.020
0.015
0.010
0.005
0.000
0
25
50
75
